# Supplementary material for: High Yield Synthesis of Curcumin and Symmetric Curcuminoids: A “Click” and “Unclick” Chemistry Approach
Source: Molecules. 2022 Dec 30;28(1):289. doi: 10.3390/molecules28010289 (PMC9822029; doi:10.3390/molecules28010289)
Supplement: Supplementary file 1 [file molecules-28-00289-s001.zip › molecules-2114552-supplementary.pdf]

molecules

Supplementary Material

High Yield Synthesis of Curcumin and Symmetric Curcuminoids: A "Click" and "Unclick" Chemistry Approach.

Curcumin synthesis is an art.

Marco A. Obregón-Mendoza[1], William Meza-Morales[1], Yair Alvarez-Ricardo[1], M. Mirian Estévez Carmona[2], and Raúl G. Enríquez[1*‡]

‡"In fond memory of our colleague Dr. Xavier Lozoya-Legorreta, d. Nov. 9, 2022".

[1]Instituto de Química, Universidad Nacional Autónoma de México, Circuito Exterior, Ciudad Universitaria, Mexico City CDMX 04510, Mexico.

[2]Departamento de Farmacia, Escuela Nacional de Ciencias Biológicas, Instituto Politécnico Nacional, Wilfrido Massieu SN, U. A. Zacatenco, Ciudad de México, C. P. 07738, México

* Correspondence: enriquezhabib@gmail.com

*Author to whom correspondence should be addressed.

Table of Contents

Figure S1. ^{1}H NMR spectrum of compound **1** (DMSO-*d6*-400MHz). 7

Figure S2. ^{1}H NMR spectrum of compound 1 (DMSO-d6-400MHz, expansion) 7

Figure S3. ^{13}C NMR spectrum of compound **1** (DMSO-*d6*-100MHz). 8

Figure S4. HSQC NMR spectrum of compound **1** (DMSO-*d6*-400MHz). 9

Figure S5. HMBC NMR spectrum of compound **1** (DMSO-*d6*-400MHz). 10

Figure S6. IR spectrum of compound **1**. 11

Figure S7. SM of compound **1** 12

Figure S8. ^{1}H NMR spectrum of compound **2** (Acetone-*d6*-400MHz) 13

Figure S9. ^{1}H NMR spectrum of compound **2** (Acetone-*d6*-400MHz, expansion) 14

Figure S10. ^{13}C NMR spectrum of compound **2** (Acetone-*d6*-100MHz). 15

Figure S11. HSQC NMR spectrum of compound **2** (Acetone-*d6*-400MHz) 16

Figure S12. HMBC NMR spectrum of compound **2** (Acetone-*d6*-400MHz). 17

Figure S13. IR spectrum of compound **2**. 18

Figure S14. SM of compound **2**. 19

Figure S15. ^{1}H NMR spectrum of compound **3** ($CDCl_3$-400MHz). 20

Figure S16. ^{1}H NMR spectrum of compound **3** ($CDCl_3$-400MHz, expansion). 21

Figure S17. ^{13}C NMR spectrum of compound **3** ($CDCl_3$-100MHz). 22

Figure S18. HSQC NMR spectrum of compound **3** ($CDCl_3$-400MHz). 23

Figure S19. HMBC NMR spectrum of compound **3** ($CDCl_3$-400MHz). 24

Figure S20. IR spectrum of compound **3.** 25
Figure S21. SM of compound **3**. 26
Figure S22. ^{1}H NMR spectrum of compound **4** (Acetone-*d6*-400MHz). 27
Figure S23. ^{1}H NMR spectrum of compound **4** (Acetone-*d6*-400MHz, expansion). 28
Figure S24. ^{13}C NMR spectrum of compound **4** (Acetone-*d6*-400MHz -100MHz). 29
Figure S25. HSQC NMR spectrum of compound **4** (Acetone-*d6*-400MHz 400MHz). 30
Figure S26. HMBC NMR spectrum of compound **4** (Acetone-*d6*-400MHz -400MHz). 31
Figure S27. IR spectrum of compound **4.** 32
Figure S28. SM of compound **4.** 33
Figure 29. ^{1}H NMR spectrum of compound **5** (DMSO-*d6*-400MHz). 34
Figure S30. ^{1}H NMR spectrum of compound **5** (DMSO-*d6*-400MHz, expansion). 35
Figure S31. ^{13}C NMR spectrum of compound **5** (DMSO-*d6*-100MHz). 36
Figure S32. DEPT spectrum of compound **5** (DMSO-*d6*-100MHz). 37
Figure S33. HSQC NMR spectrum of compound **5** (DMSO-*d6*-400MHz). 38
Figure S34. HMBC NMR spectrum of compound **5** (DMSO-*d6*-400MHz). 40
Figure S35. IR spectrum of compound **5**. 41
Figure S36. SM of compound **5**. 42
Figure S37. UV-spectrum of compound **5**. 43
Figure S38. HPLC of compound **5** (425nm). 44
Figure 39. ^{1}H NMR spectrum of compound **6** (Acetone-*d6*-400MHz). 45
Figure S40. ^{1}H NMR spectrum of compound **6** (Acetone-*d6*-400MHz, expansion). 46

Figure S41. ^{13}C NMR spectrum of compound **6** (Acetone-*d6*-100MHz). 47
Figure S42. HSQC NMR spectrum of compound **6** (Acetone-*d6*-400MHz). 48
Figure S43. HMBC NMR spectrum of compound **6** (Acetone-*d6*-400MHz). 49
Figure S44. IR spectrum of compound **6**. 50
Figure S45. SM of compound **6**. 51
Figure S46. UV-spectrum of compound **6**. 52
Figure S47. HPLC of compound **6** (425nm). 53
Figure 48. ^{1}H NMR spectrum of compound **7** (DMSO-*d6*-400MHz). 54
Figure S49. ^{1}H NMR spectrum of compound **7** (DMSO-*d6*-400MHz, expansion). 55
Figure S50. ^{13}C NMR spectrum of compound **7** (DMSO-*d6*-100MHz). 56
Figure S51. HSQC NMR spectrum of compound **7** (DMSO-*d6*-400MHz). 57
Figure S52. HMBC NMR spectrum of compound **7** (DMSO-*d6*-400MHz). 58
Figure S53. IR spectrum of compound **7**. 59
Figure S54. SM of compound **7**. 60
Figure S55. UV-spectrum of compound **7**. 61
Figure S56. HPLC of compound **7** (425nm). 62
Figure 57. ^{1}H NMR spectrum of compound **8** (DMSO-*d6*-400MHz). 63
Figure S58. ^{1}H NMR spectrum of compound **8** (DMSO-*d6*-400MHz, expansion). 64
Figure S59. ^{13}C NMR spectrum of compound **8** (DMSO-*d6*-100MHz). 65
Figure S60. HSQC NMR spectrum of compound **8** (DMSO-*d6*-400MHz). 66
Figure S61. HMBC NMR spectrum of compound **8** (DMSO-*d6*-400MHz). 67

Figure S62. IR spectrum of compound **8**. 68
Figure S63. SM of compound **8**. 69
Figure S64. UV-spectrum of compound **8**. 70
Figure S65. HPLC of compound **8** (425nm). 71
Figure 66. ^{1}H NMR spectrum of *synthon I* ($CDCl_3$-400MHz). 72
Figure S67. ^{13}C NMR spectrum of *synthon I* ($CDCl_3$-100MHz). 73
Figure S68. HSQC NMR spectrum of *synthon I* ($CDCl_3$-400MHz). 74
Figure S69. HMBC NMR spectrum of *synthon I* ($CDCl_3$-400MHz). 75
Figure S70. IR spectrum of *synthon I*. 76
Figure S71. SM of *synthon I*. 77

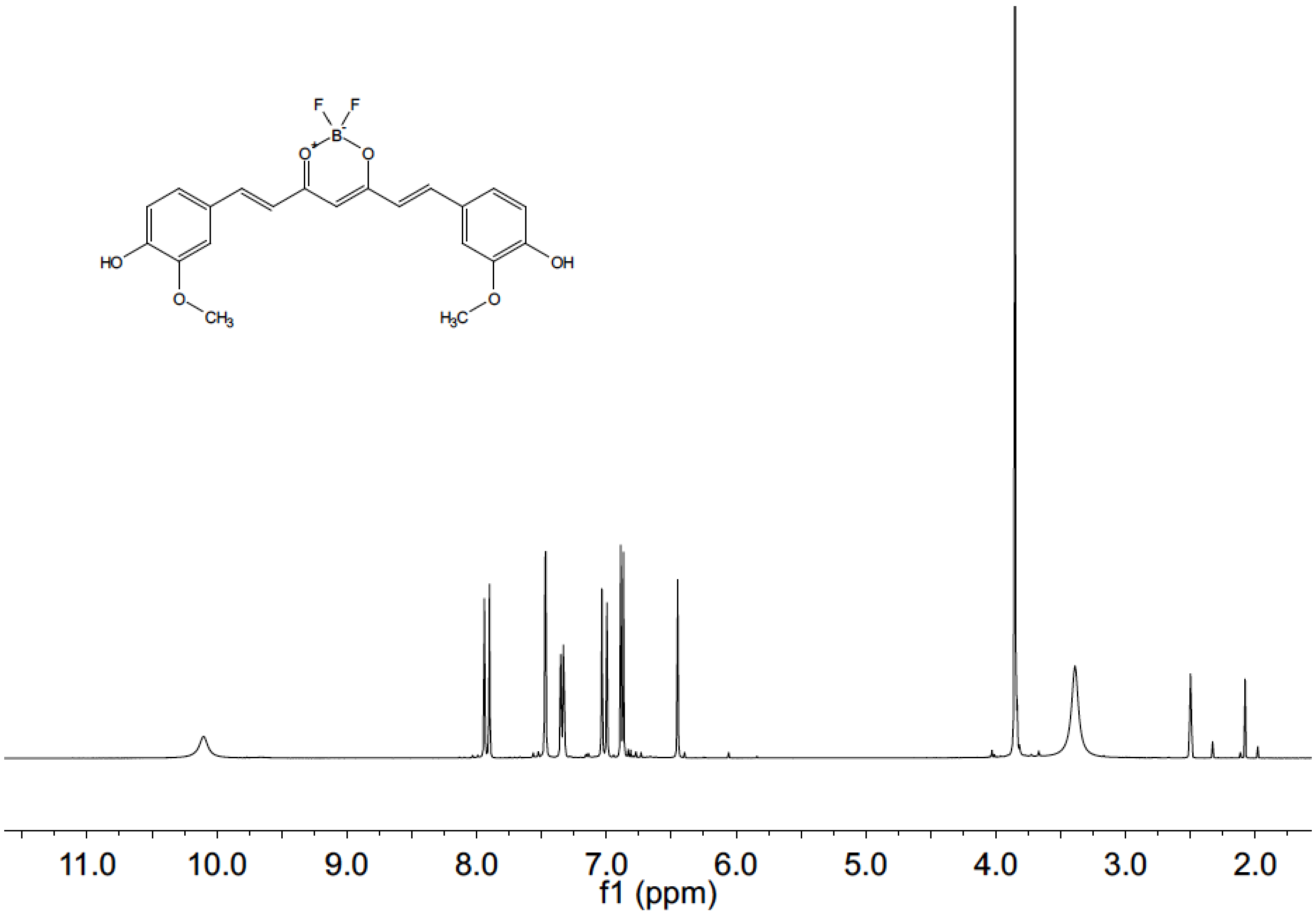
F
F
B
O
O
HO
OH
O
CH3
H3C
O
11.0
10.0
9.0
8.0
7.0
6.0
5.0
4.0
3.0
2.0
f1 (ppm)

Figure S1. ¹H NMR spectrum of compound **1** (DMSO-*d6*-400MHz).

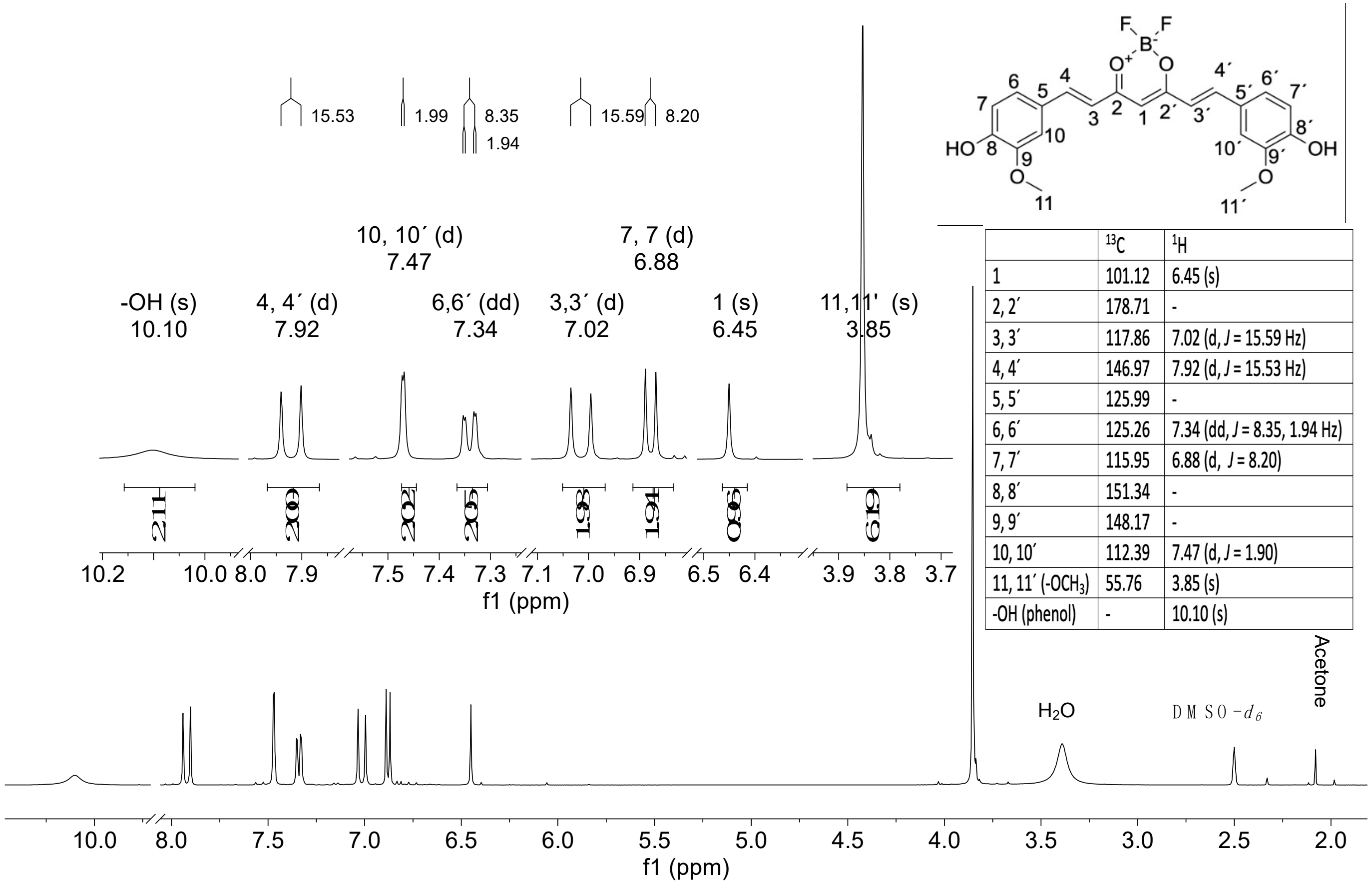

	^{13}C	^1H
1	101.12	6.45 (s)
2, 2´	178.71	-
3, 3´	117.86	7.02 (d, *J* = 15.59 Hz)
4, 4´	146.97	7.92 (d, *J* = 15.53 Hz)
5, 5´	125.99	-
6, 6´	125.26	7.34 (dd, *J* = 8.35, 1.94 Hz)
7, 7´	115.95	6.88 (d, *J* = 8.20)
8, 8´	151.34	-
9, 9´	148.17	-
10, 10´	112.39	7.47 (d, *J* = 1.90)
11, 11´ (-OCH_3)	55.76	3.85 (s)
-OH (phenol)	-	10.10 (s)

Figure S2. ¹H NMR spectrum of compound 1 (DMSO-d6-400MHz, expansion).

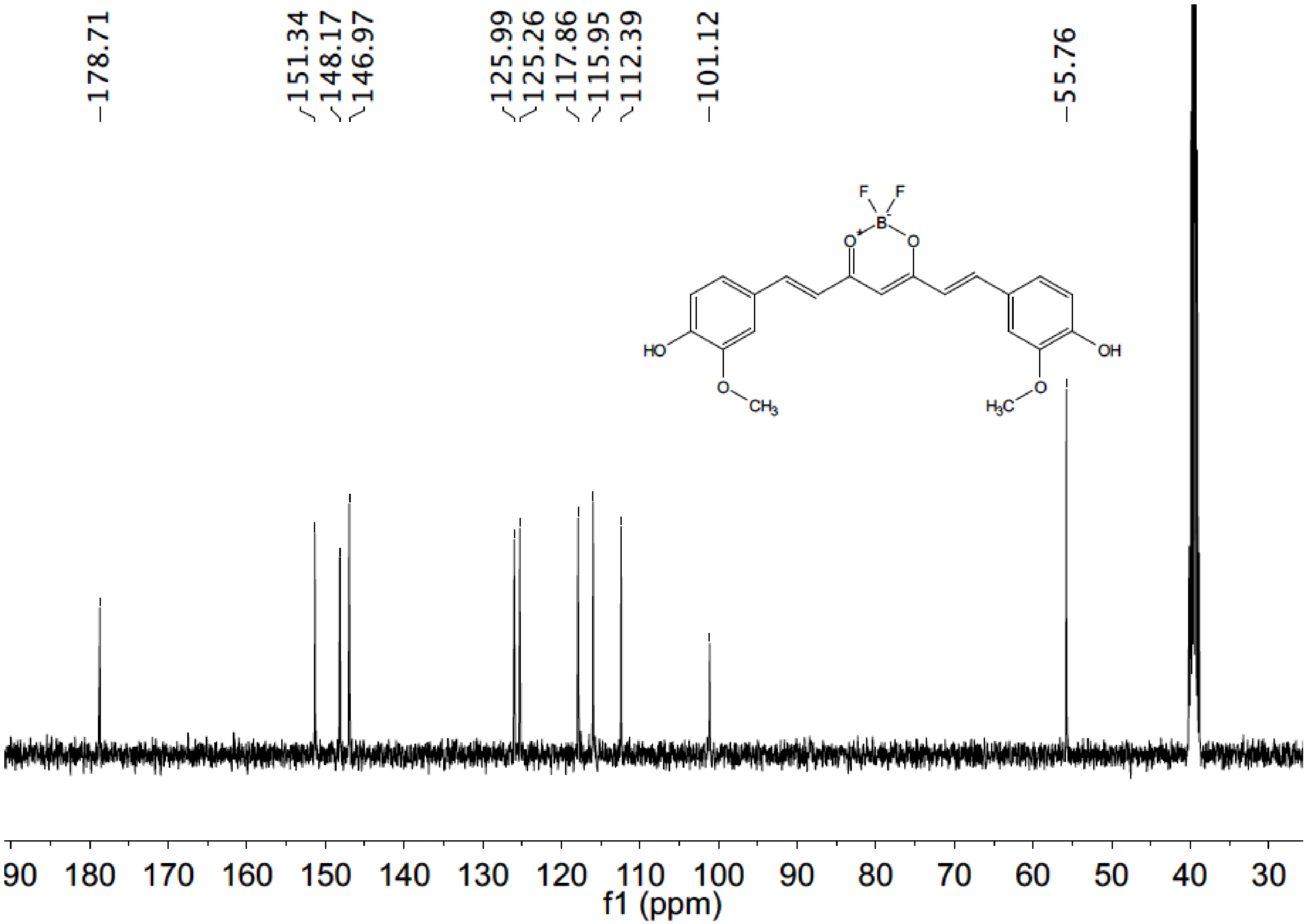

Figure S3. ^{13}C NMR spectrum of compound **1** (DMSO-*d6*-100MHz).

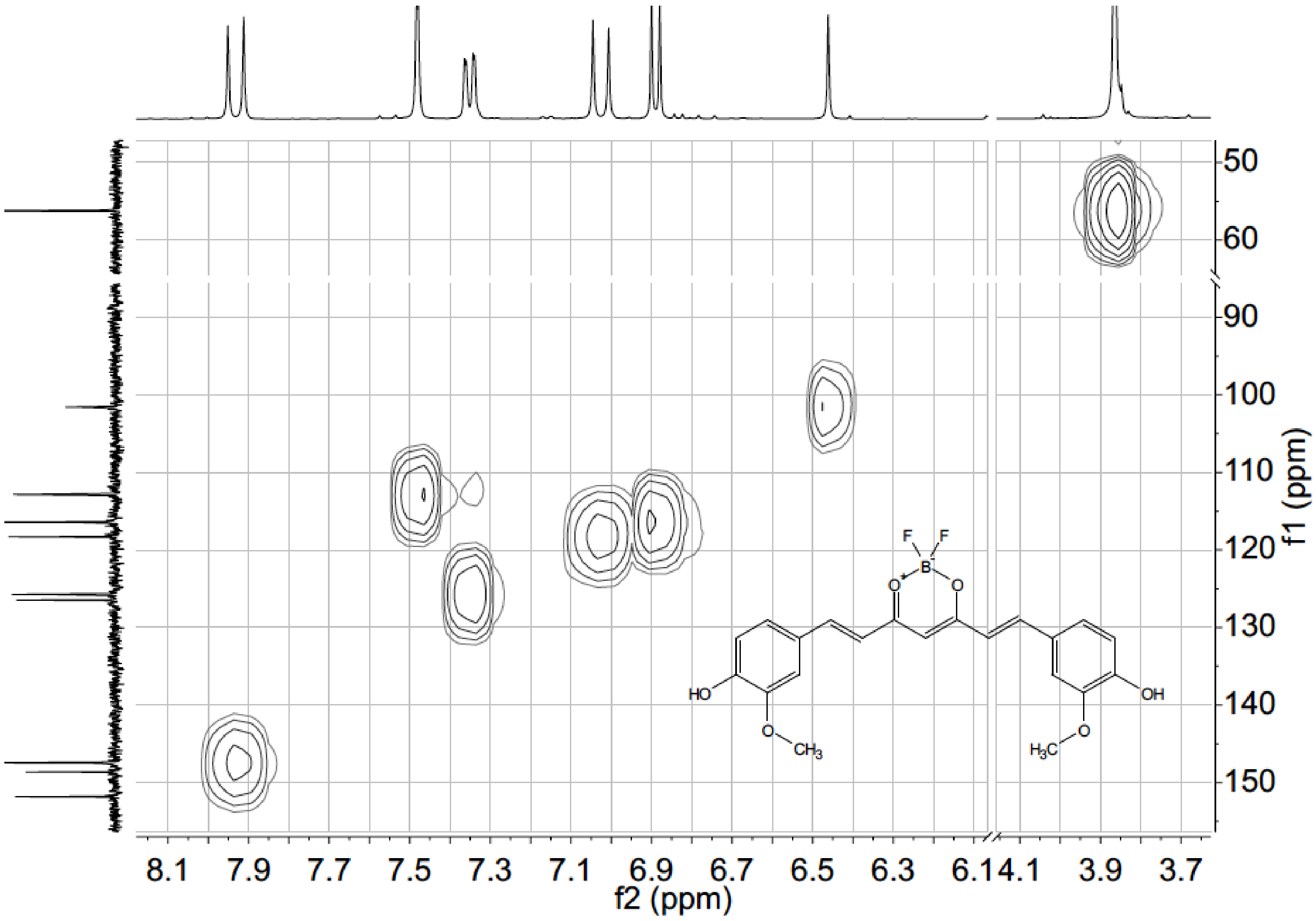

Figure S4. HSQC NMR spectrum of compound **1** (DMSO-*d6*-400MHz).

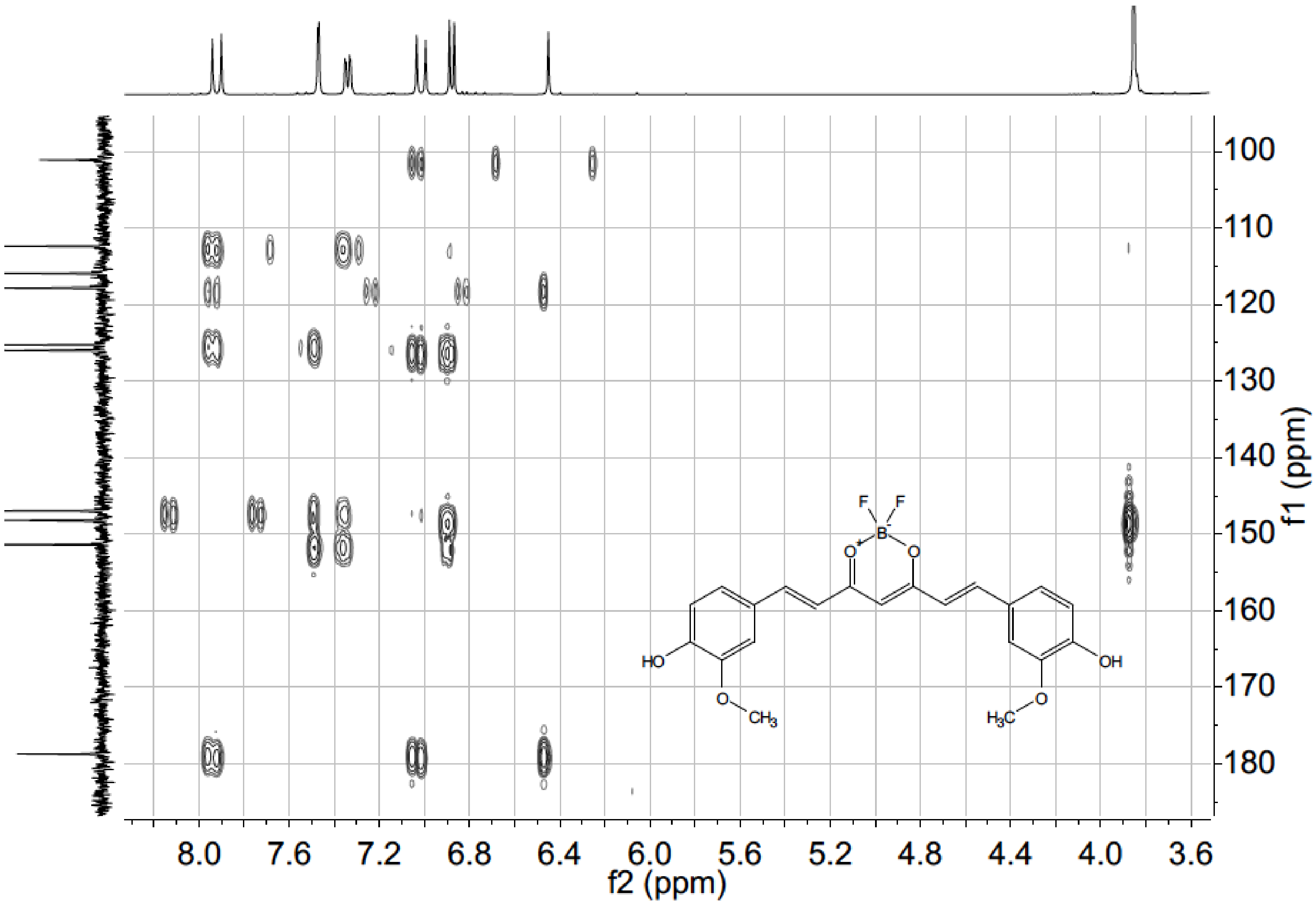

Figure S5. HMBC NMR spectrum of compound **1** (DMSO-*d6*-400MHz).

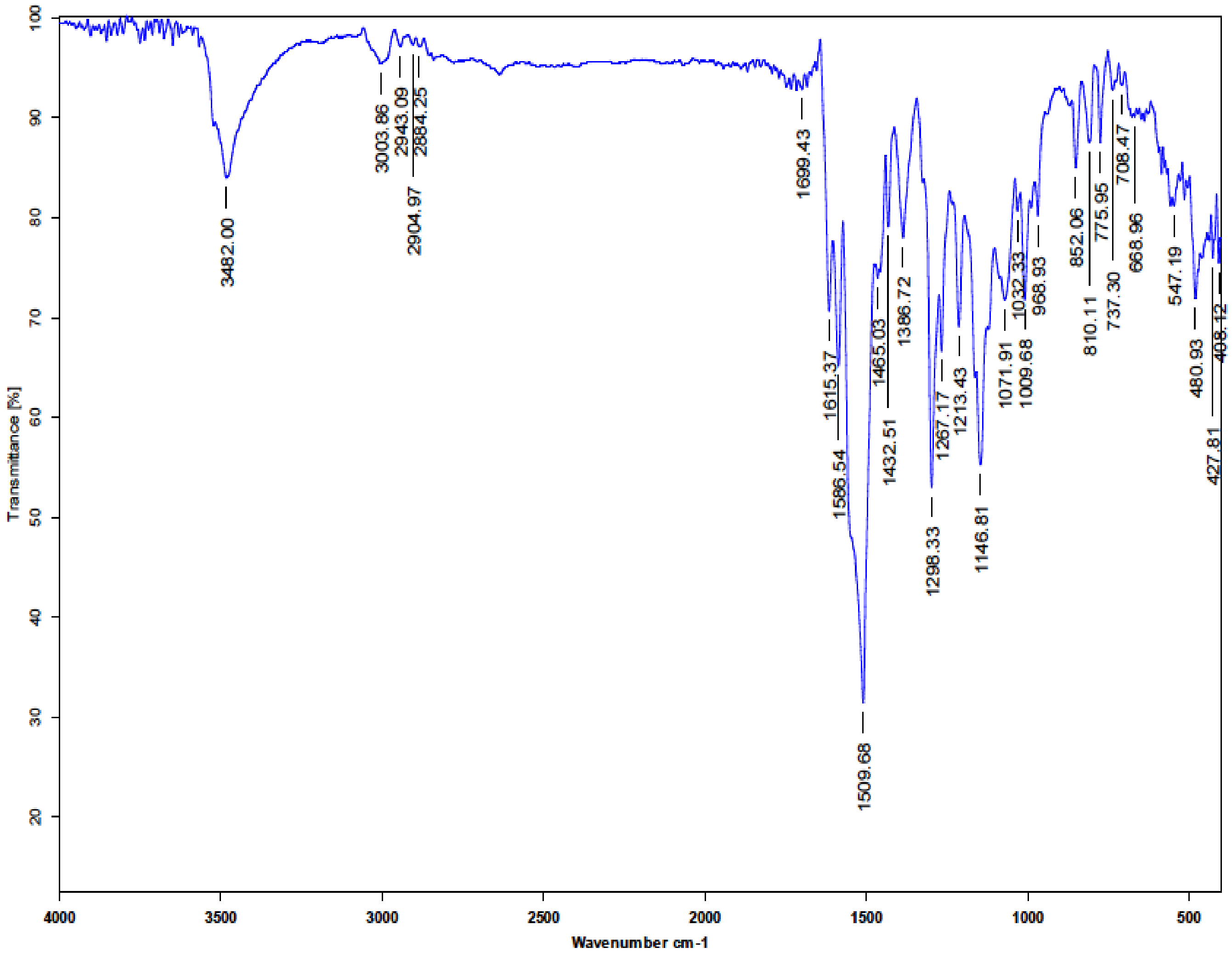

Figure S6. IR spectrum of compound **1**.

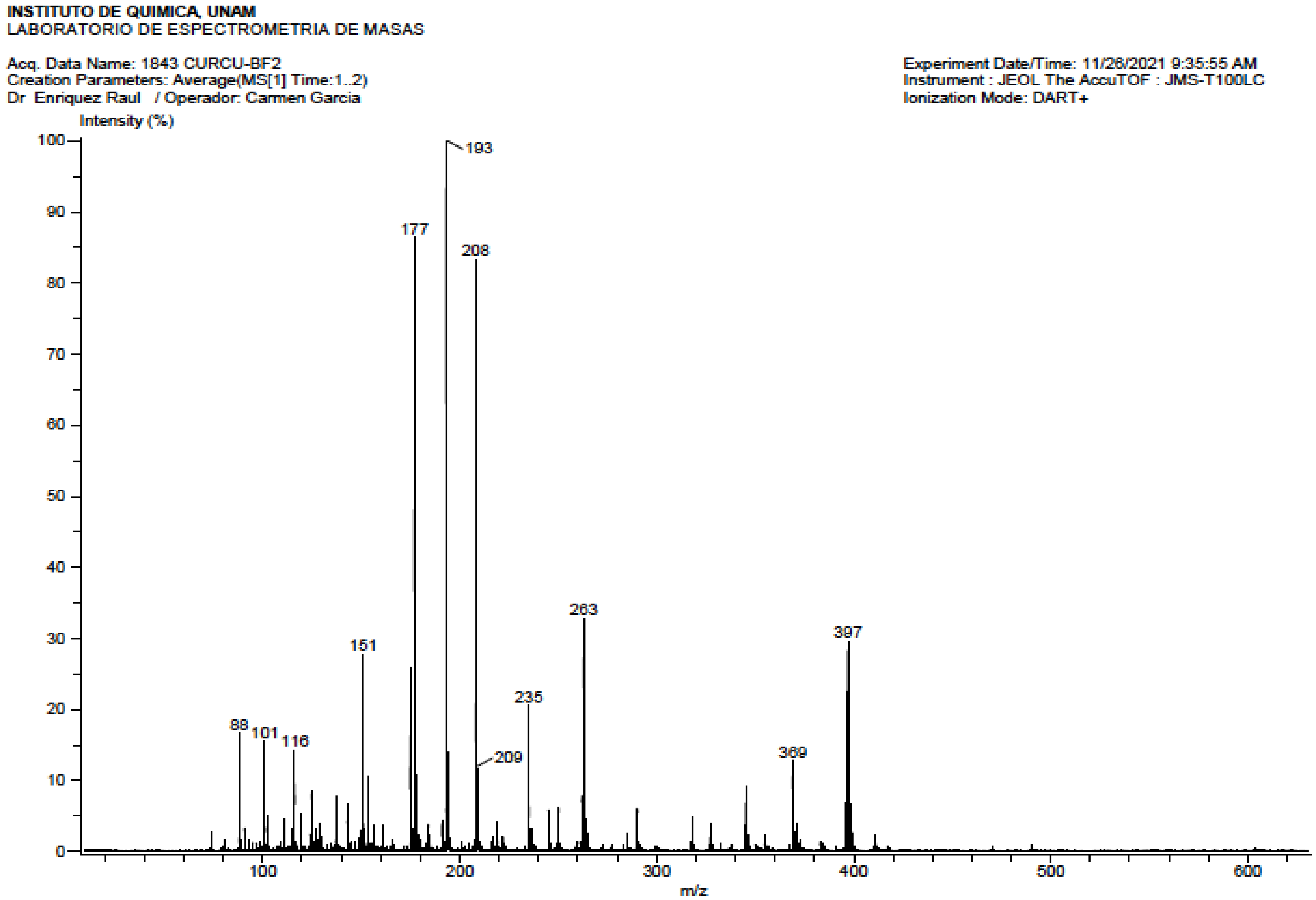

Figure S7. SM of compound **1**.

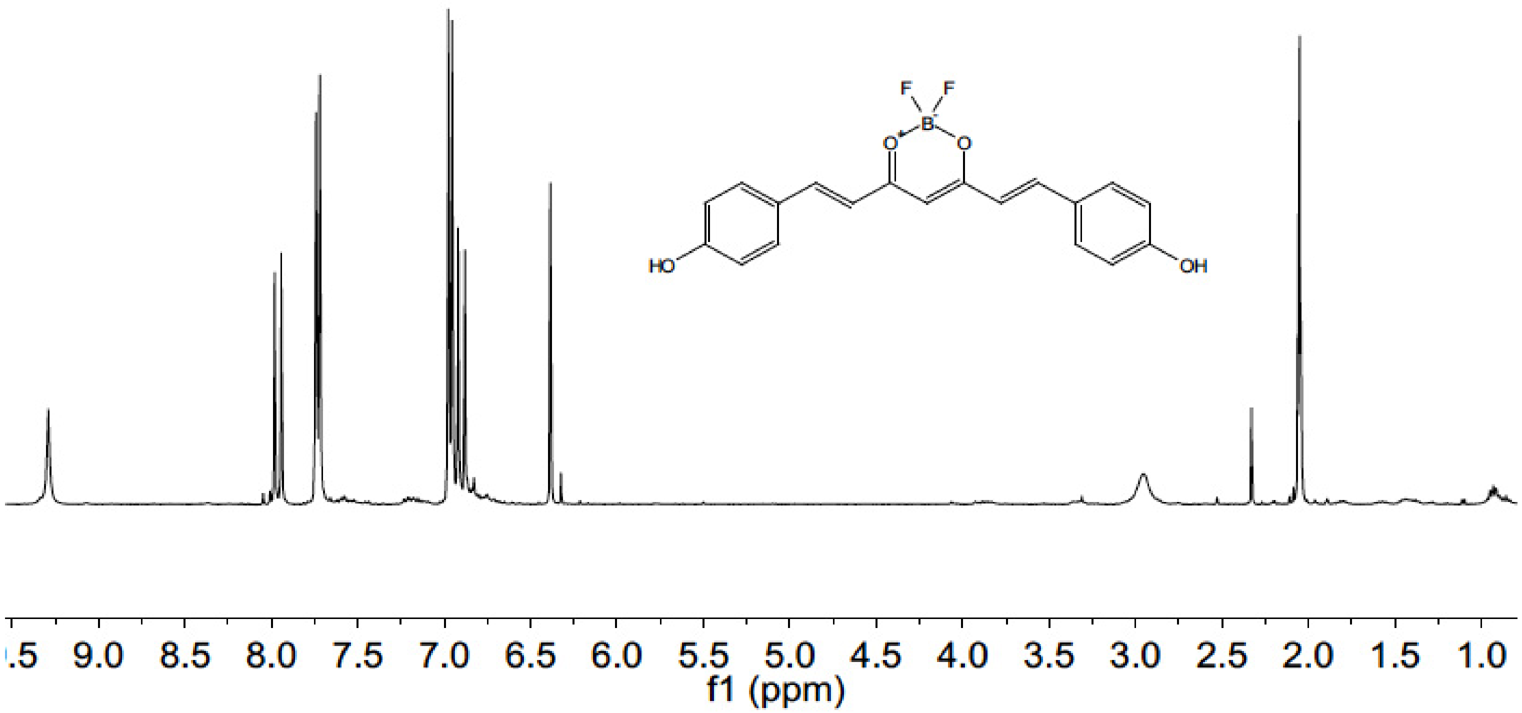

Figure S8. ^{1}H NMR spectrum of compound **2** (Acetone-*d6*-400MHz).

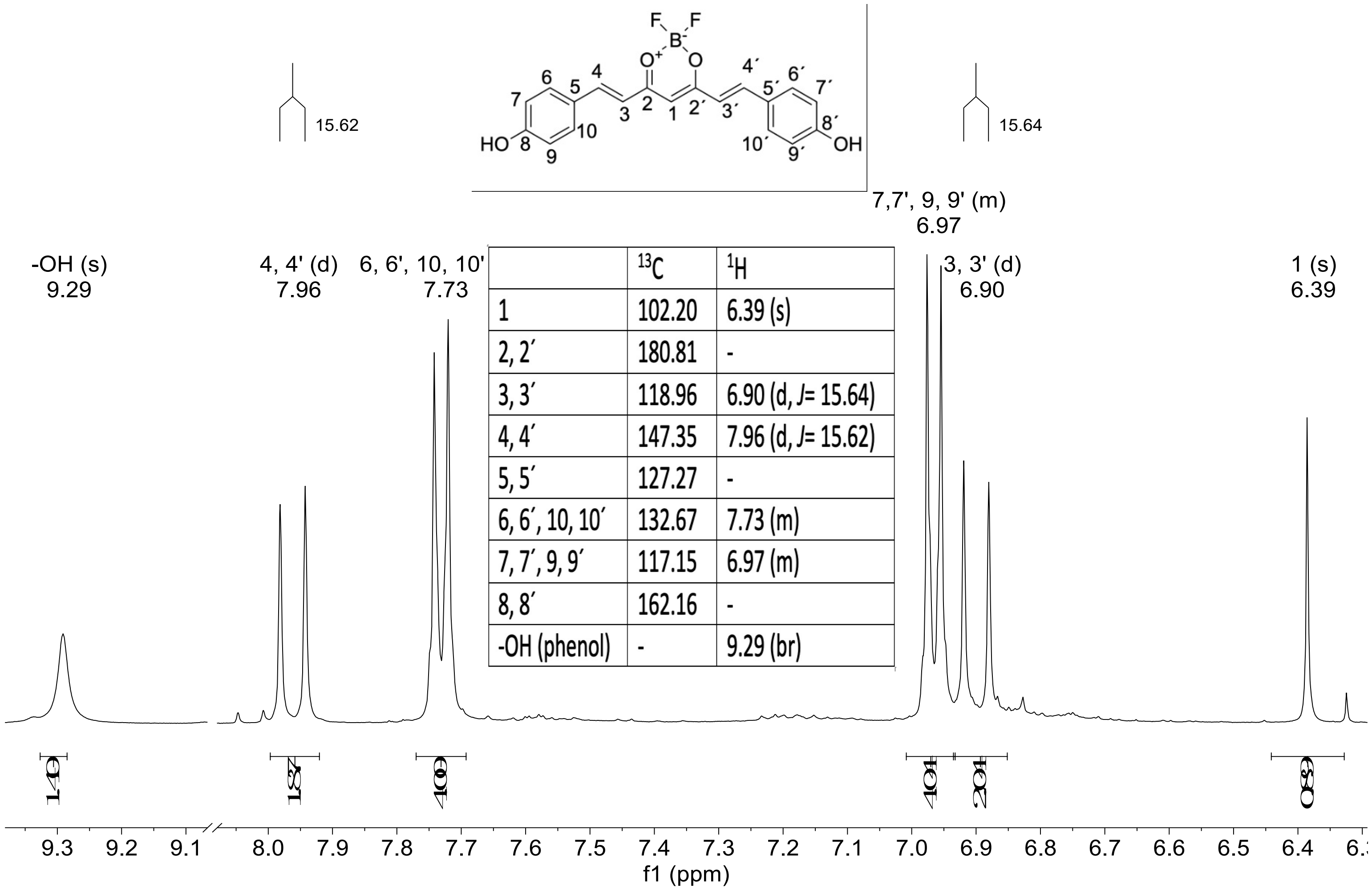

	^{13}C	^{1}H
1	102.20	6.39 (s)
2, 2′	180.81	-
3, 3′	118.96	6.90 (d, *J*= 15.64)
4, 4′	147.35	7.96 (d, *J*= 15.62)
5, 5′	127.27	-
6, 6′, 10, 10′	132.67	7.73 (m)
7, 7′, 9, 9′	117.15	6.97 (m)
8, 8′	162.16	-
-OH (phenol)	-	9.29 (br)

Figure S9. ^{1}H NMR spectrum of compound **2** (Acetone-*d6*-400MHz, expansion).

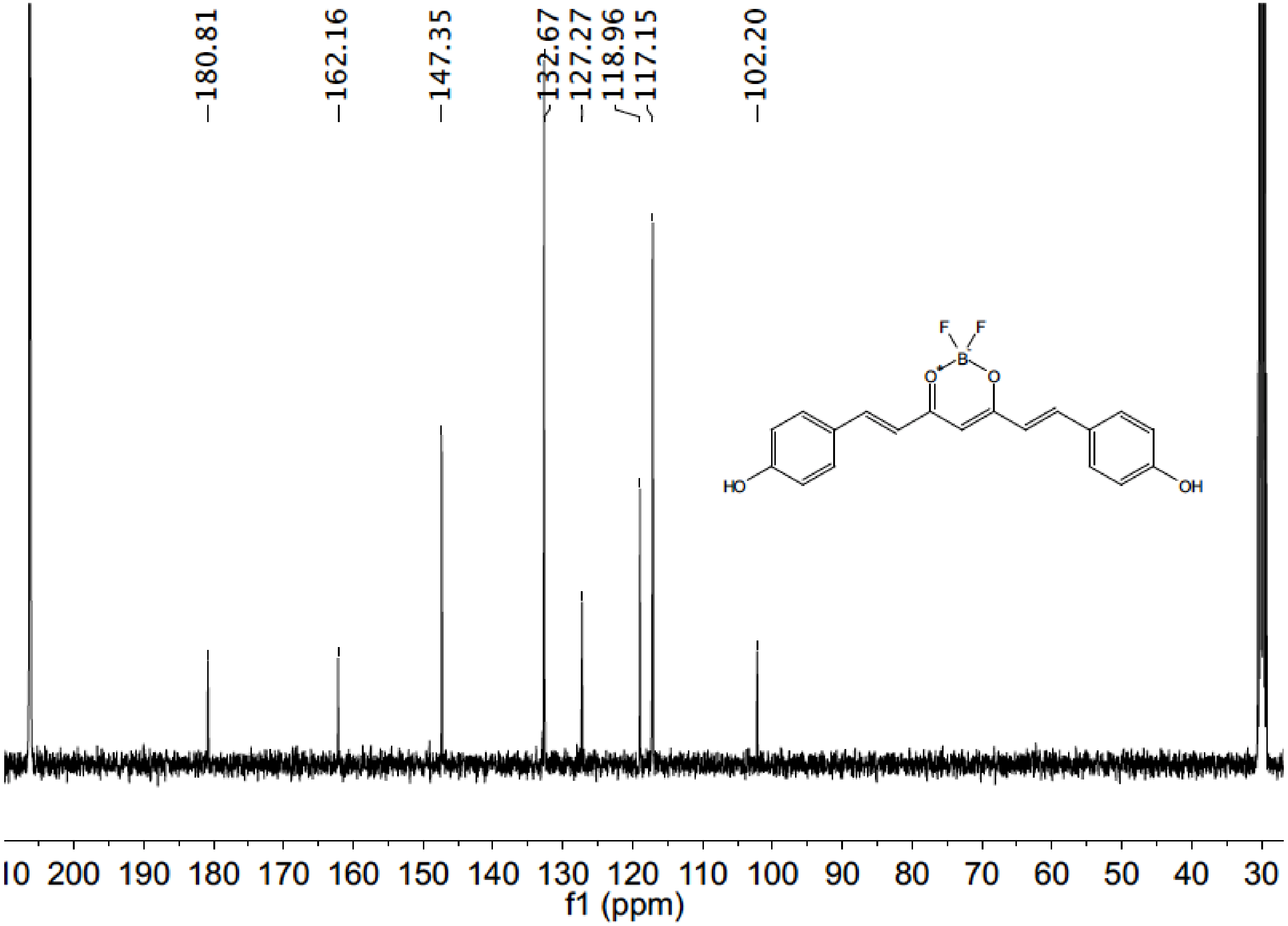

Figure S10. ^{13}C NMR spectrum of compound **2** (Acetone-*d6*-100MHz).

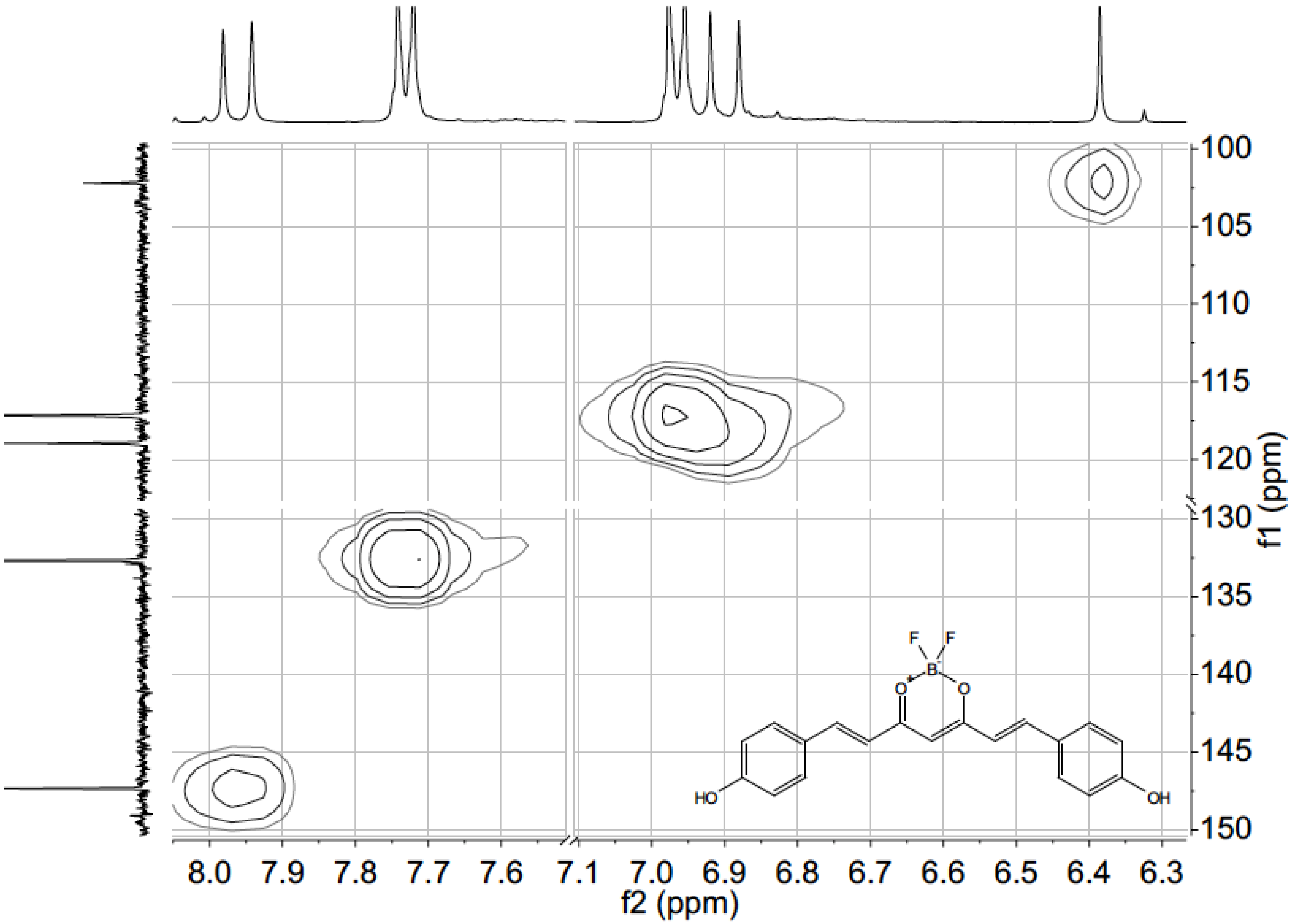

Figure S11. HSQC NMR spectrum of compound **2** (Acetone-*d6*-400MHz).

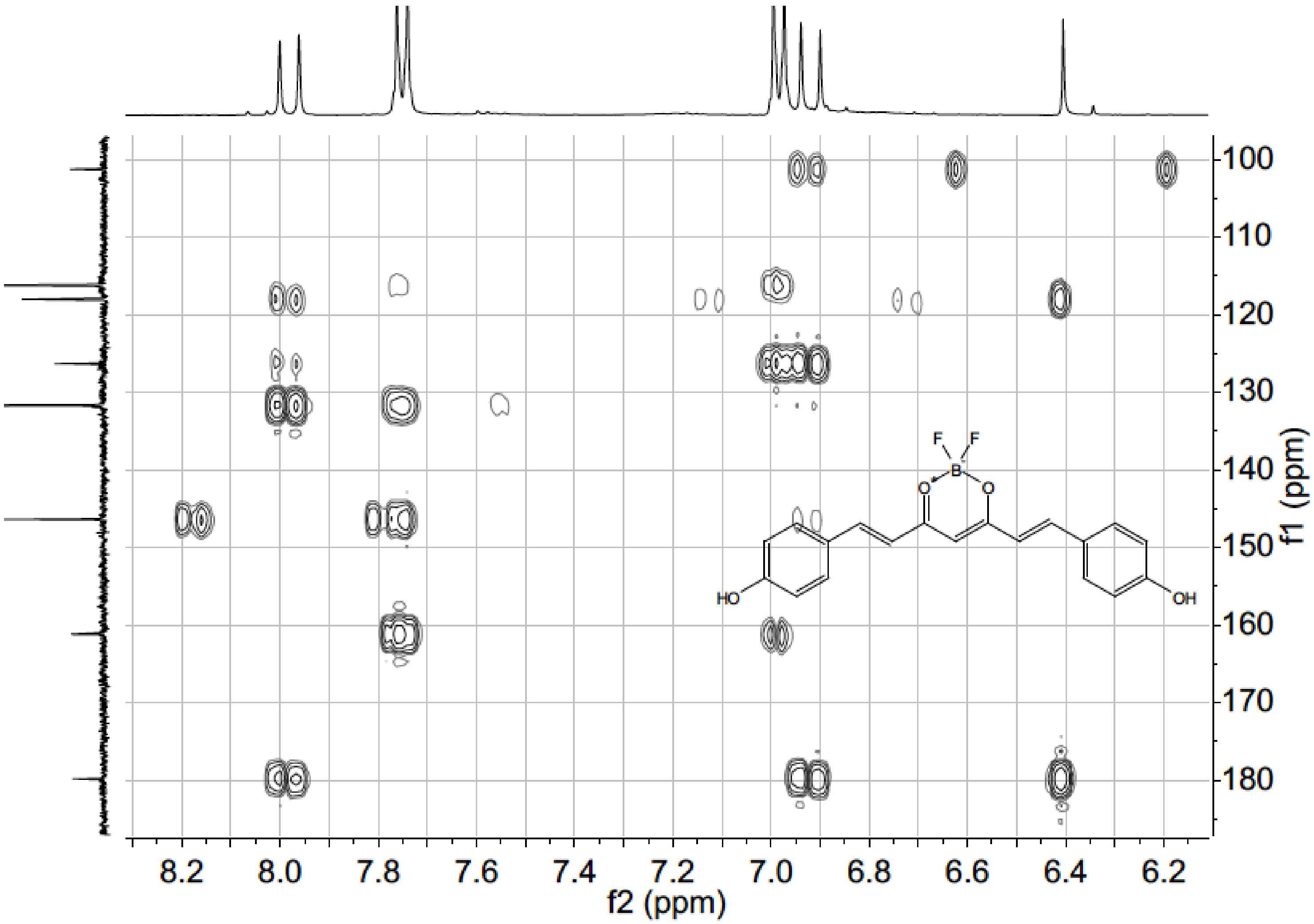

Figure S12. HMBC NMR spectrum of compound **2** (Acetone-*d6*-400MHz).

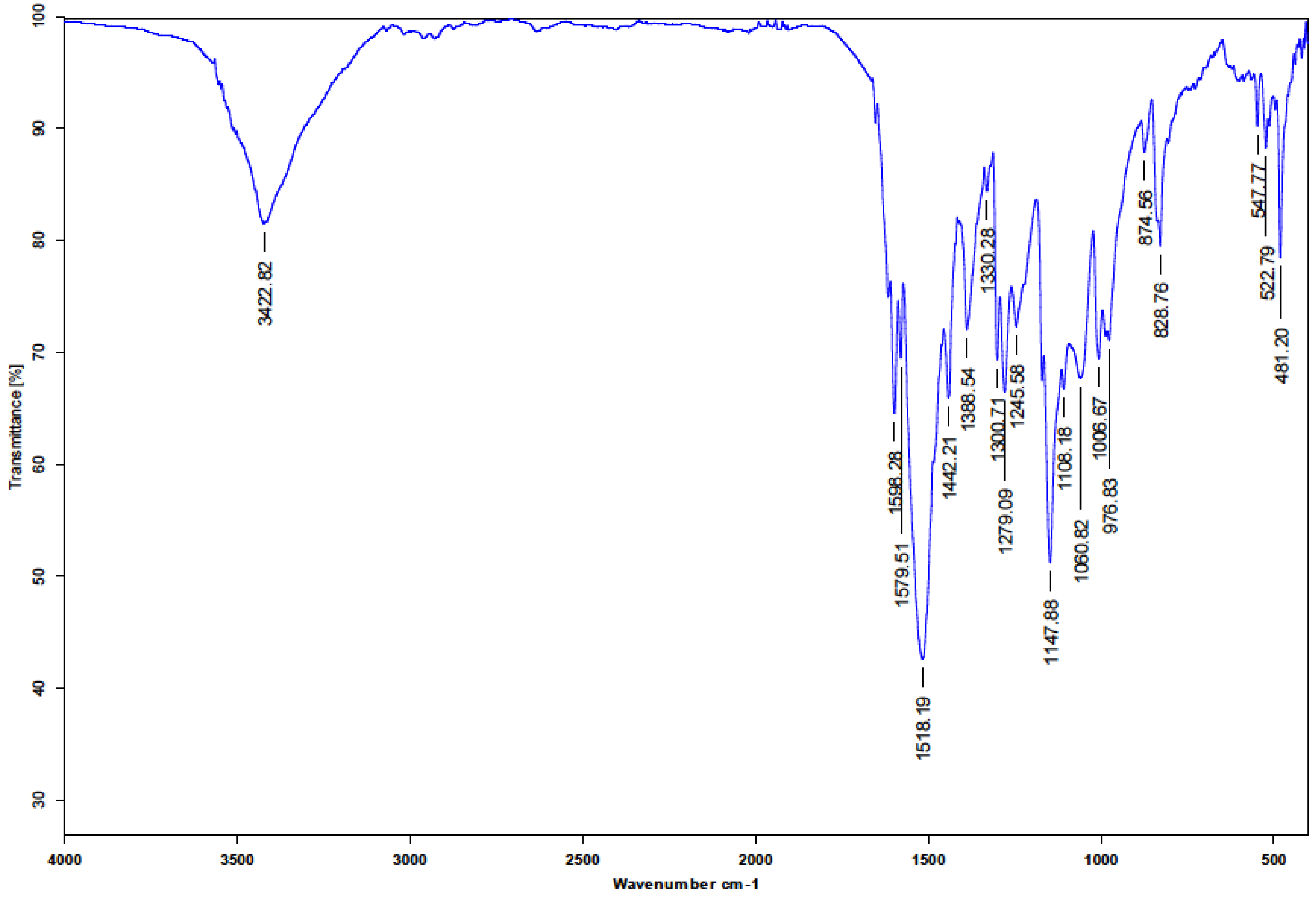

Figure S13. IR spectrum of compound **2**.

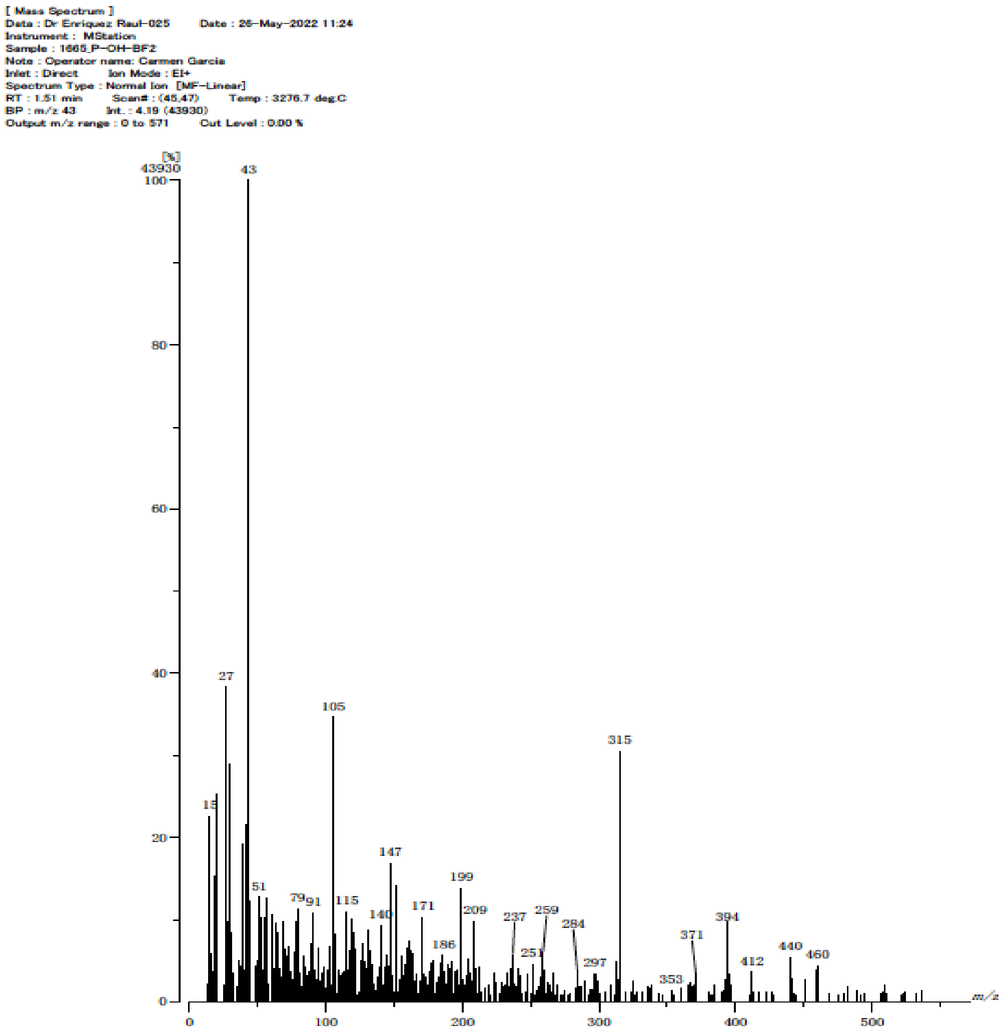

Figure S14. SM of compound **2**.

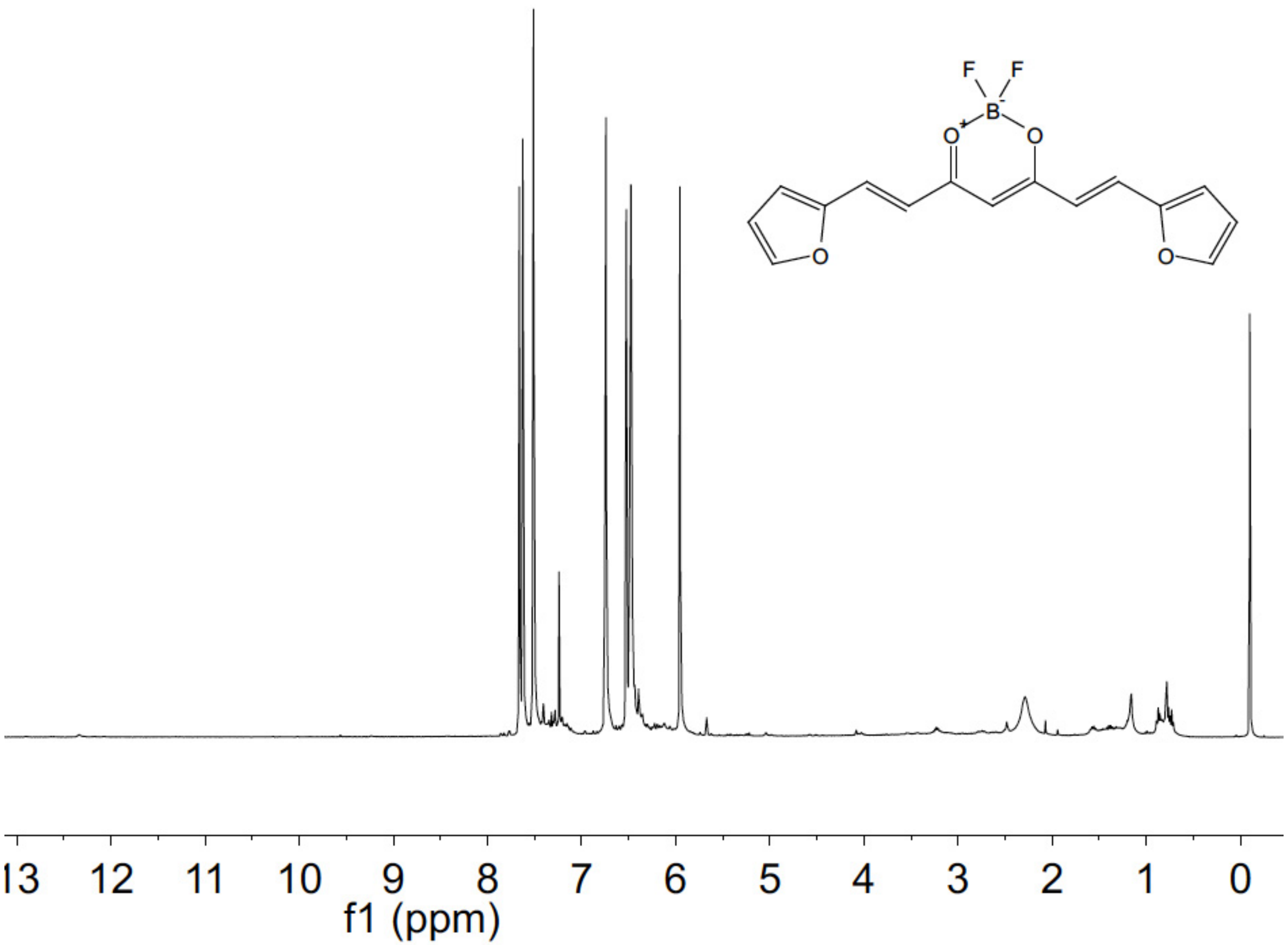

Figure S15. ^{1}H NMR spectrum of compound **3** ($CDCl_3$-400MHz).

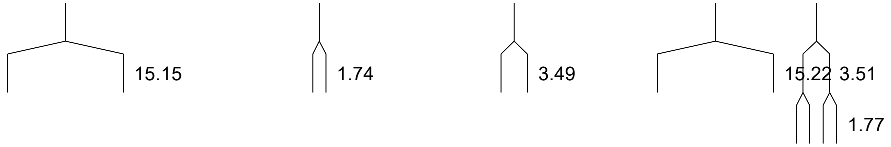

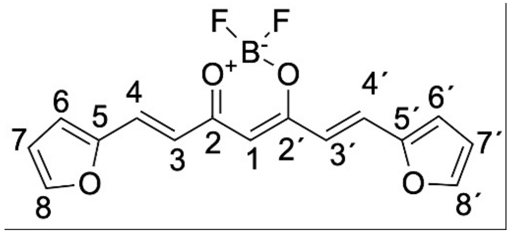

	^{13}C	^{1}H
1	102.34	5.96 (s)
2,2´	178.98	-
3,3´	117.95	6.51 (d, *J* = 15.22)
4,4´	131.91	7.64 (d, *J* = 15.15)
5,5´	151.01	-
6,6´	119.04	6.75 (d, *J* = 3.49)
7,7´	113.37	6.47 (dd, *J* = 3.51, 1.77)
8,8´	146.78	7.51 (d, *J* = 1.74)

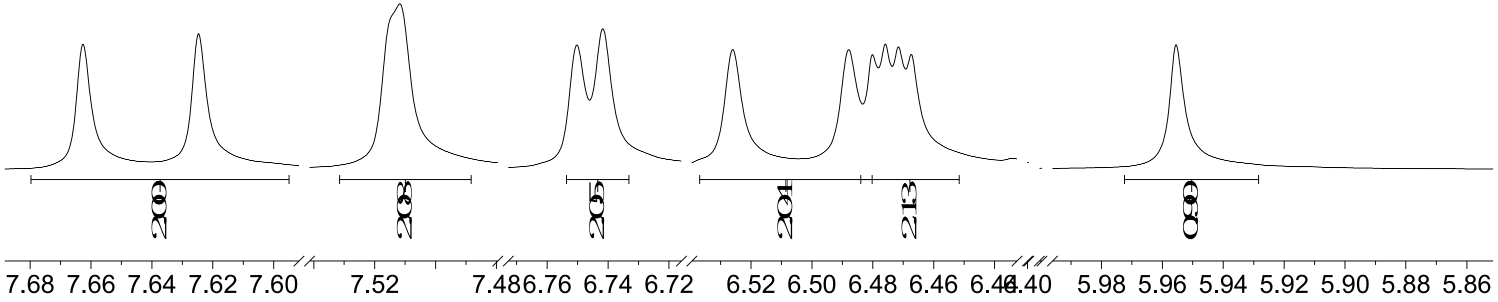

Figure S16. ^{1}H NMR spectrum of compound **3** ($CDCl_3$-400MHz, expansion).

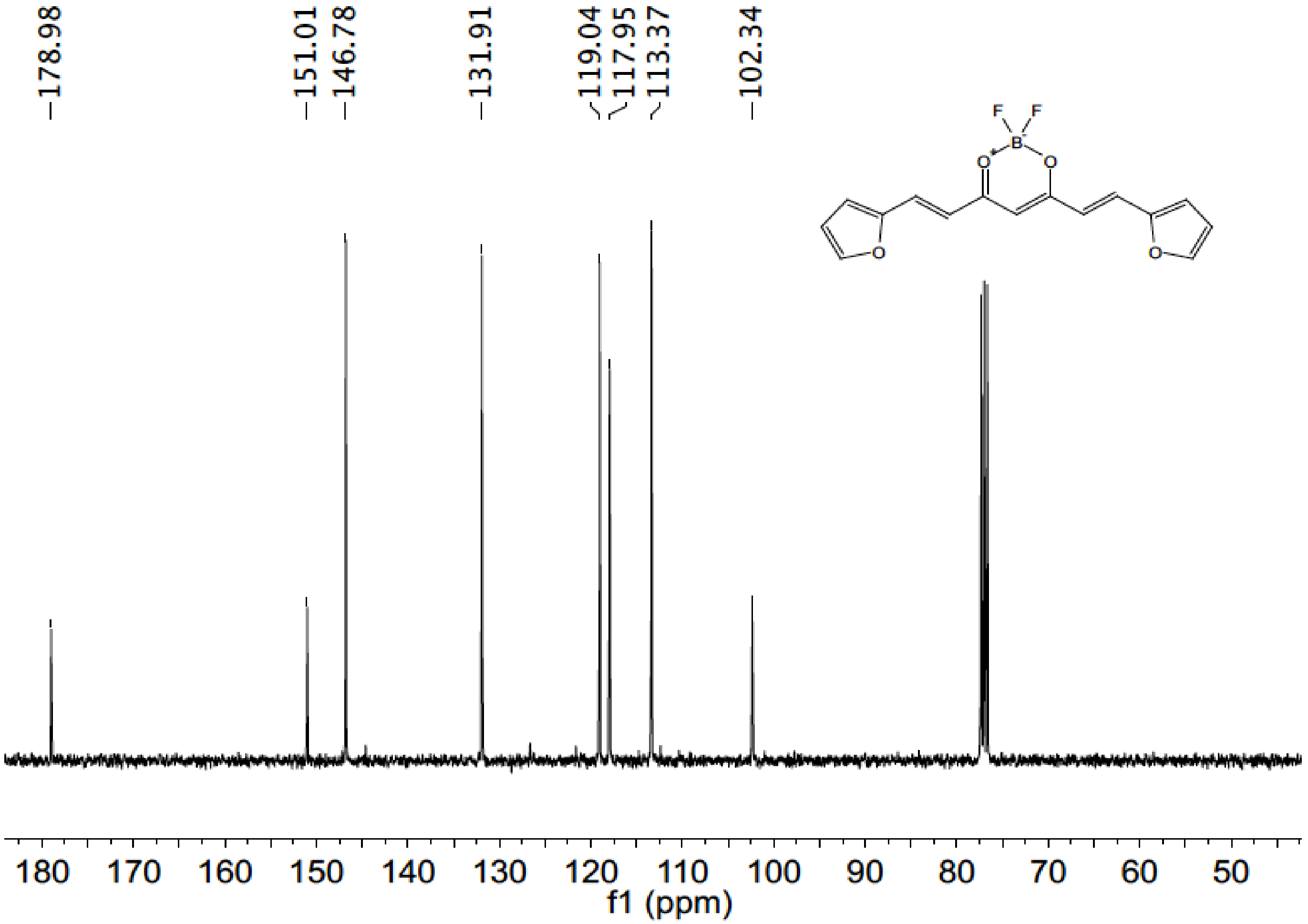

Figure S17. ^{13}C NMR spectrum of compound **3** ($CDCl_3$-100MHz).

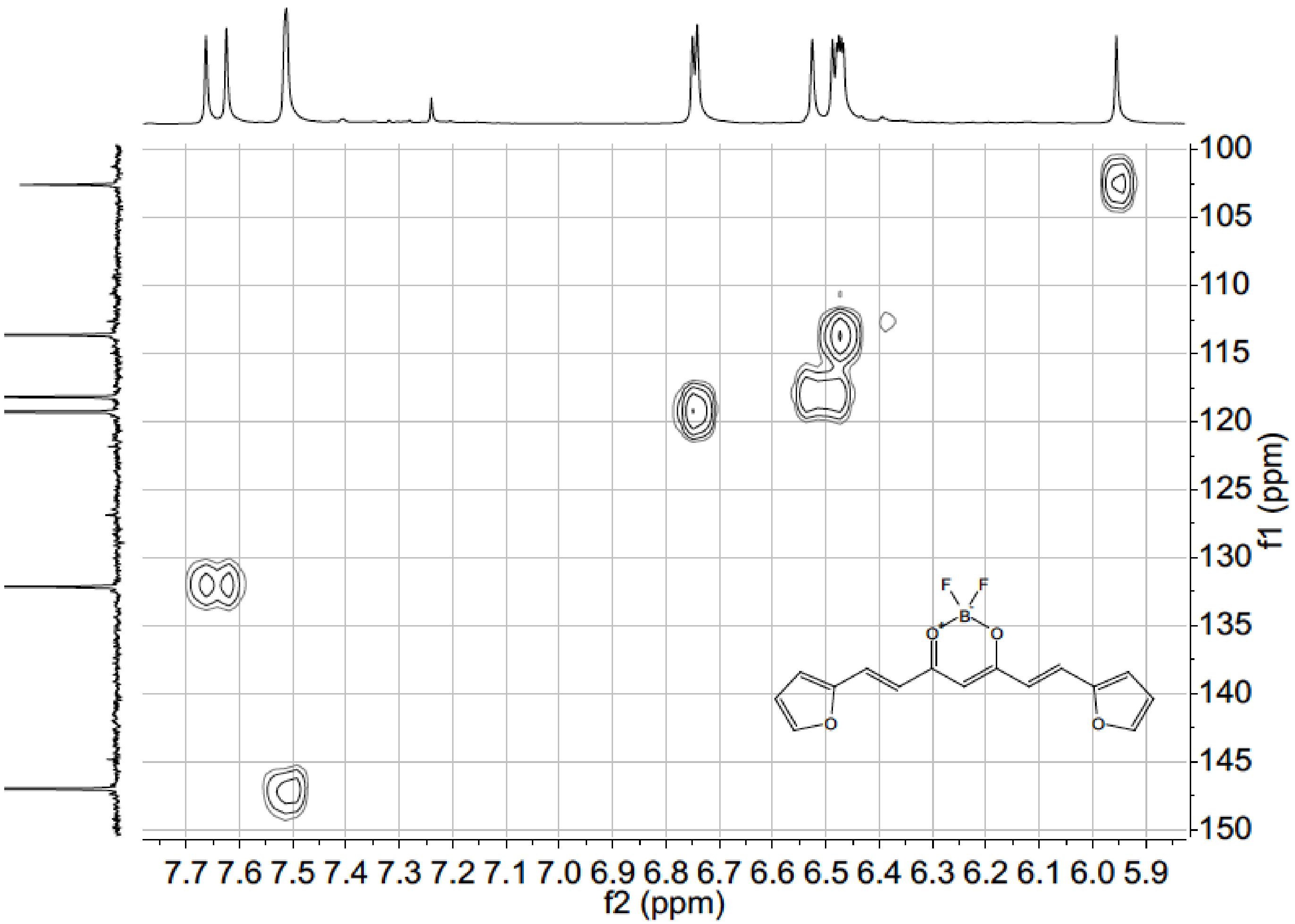

Figure S18. HSQC NMR spectrum of compound **3** ($CDCl_3$-400MHz).

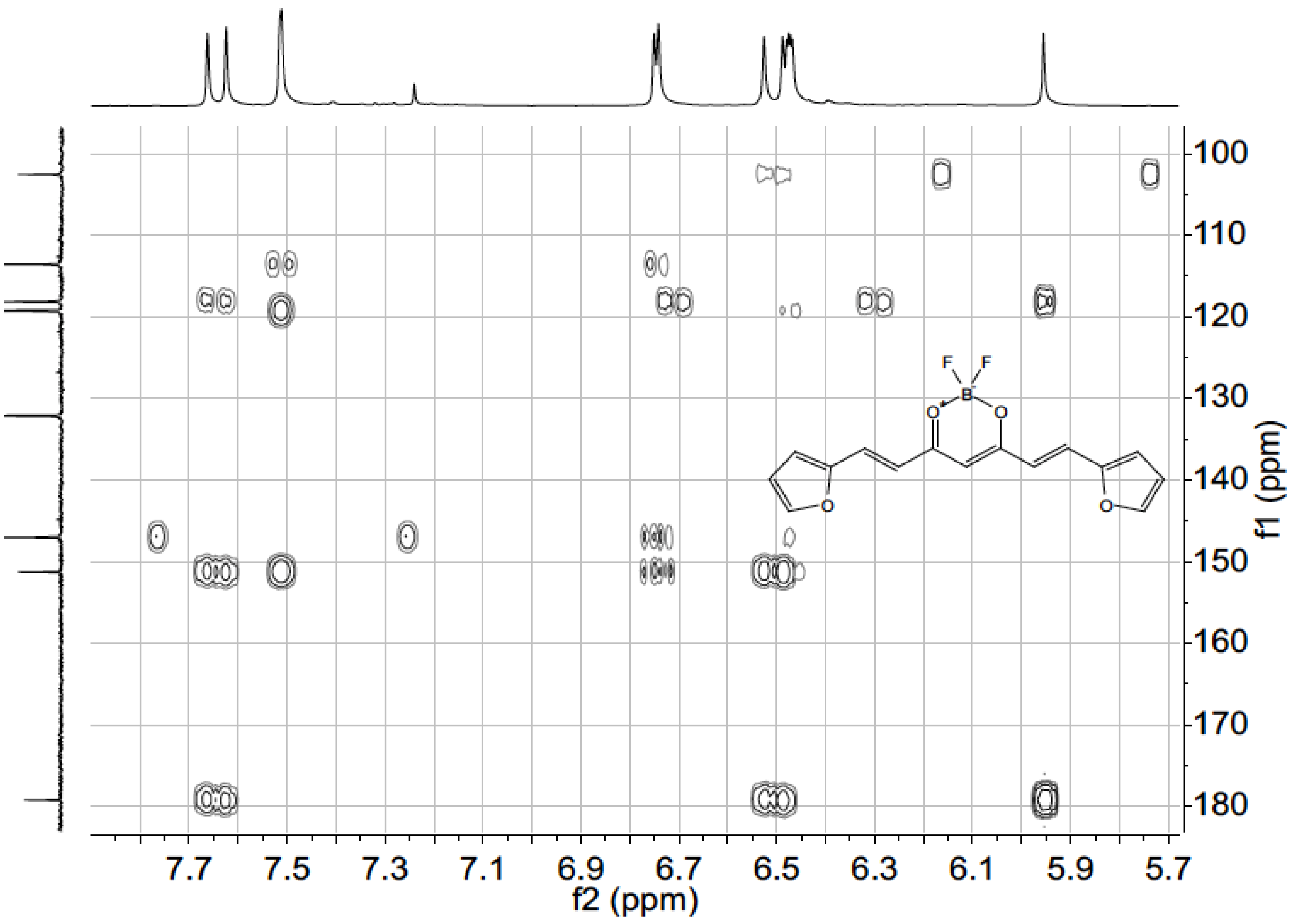

Figure S19. HMBC NMR spectrum of compound **3** ($CDCl_3$-400MHz).

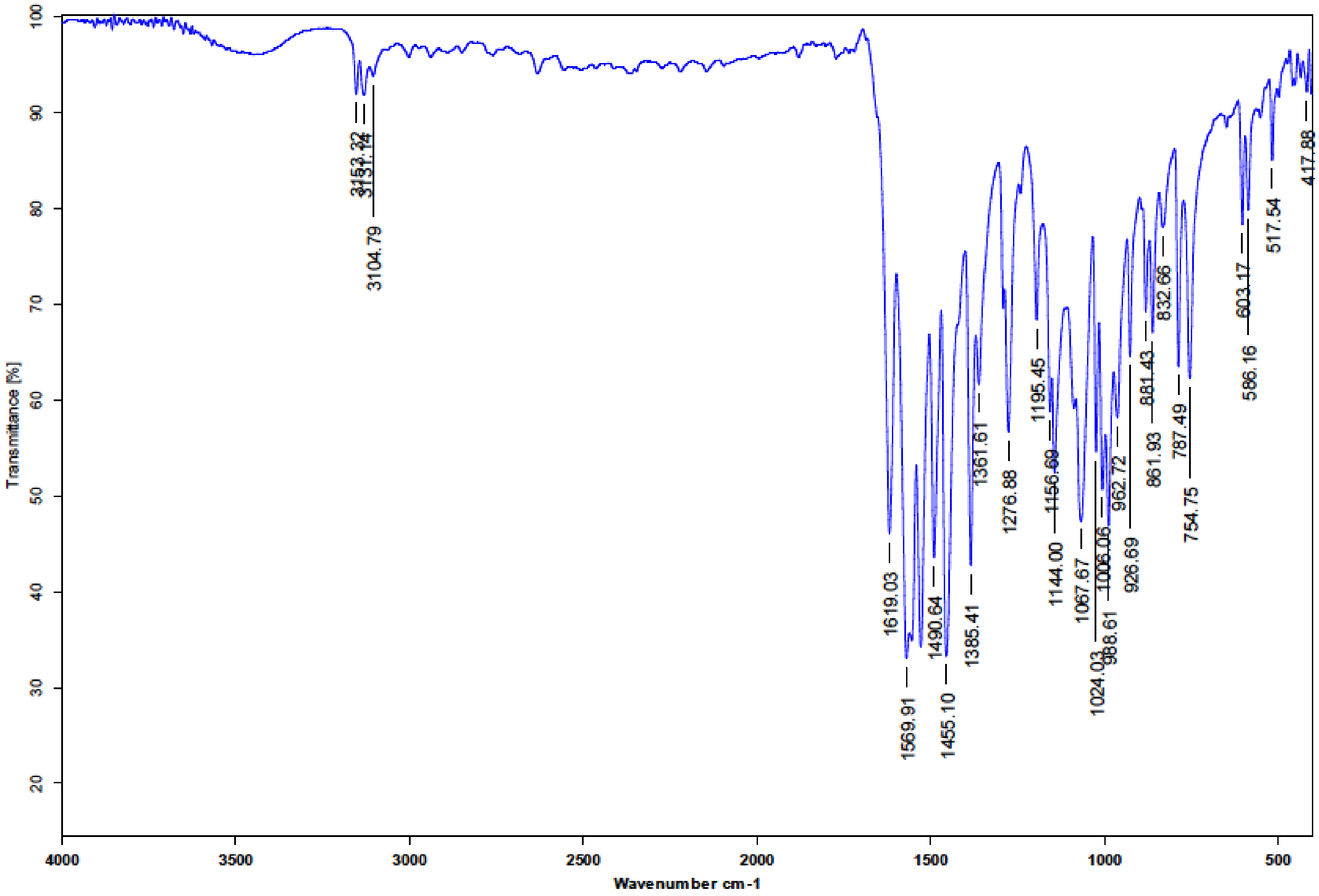

Figure S20. IR spectrum of compound **3**.

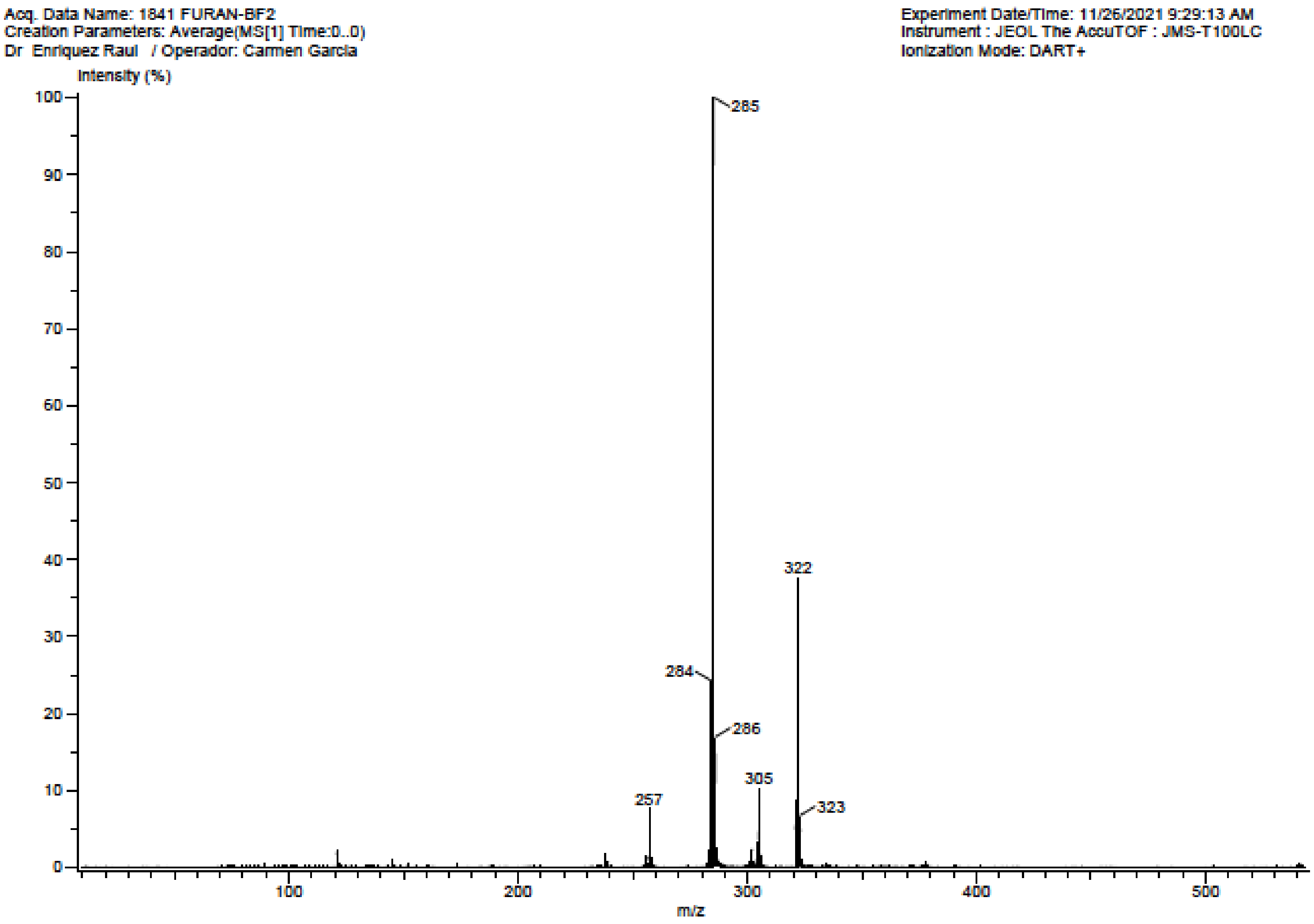

Figure S21. SM of compound **3**.

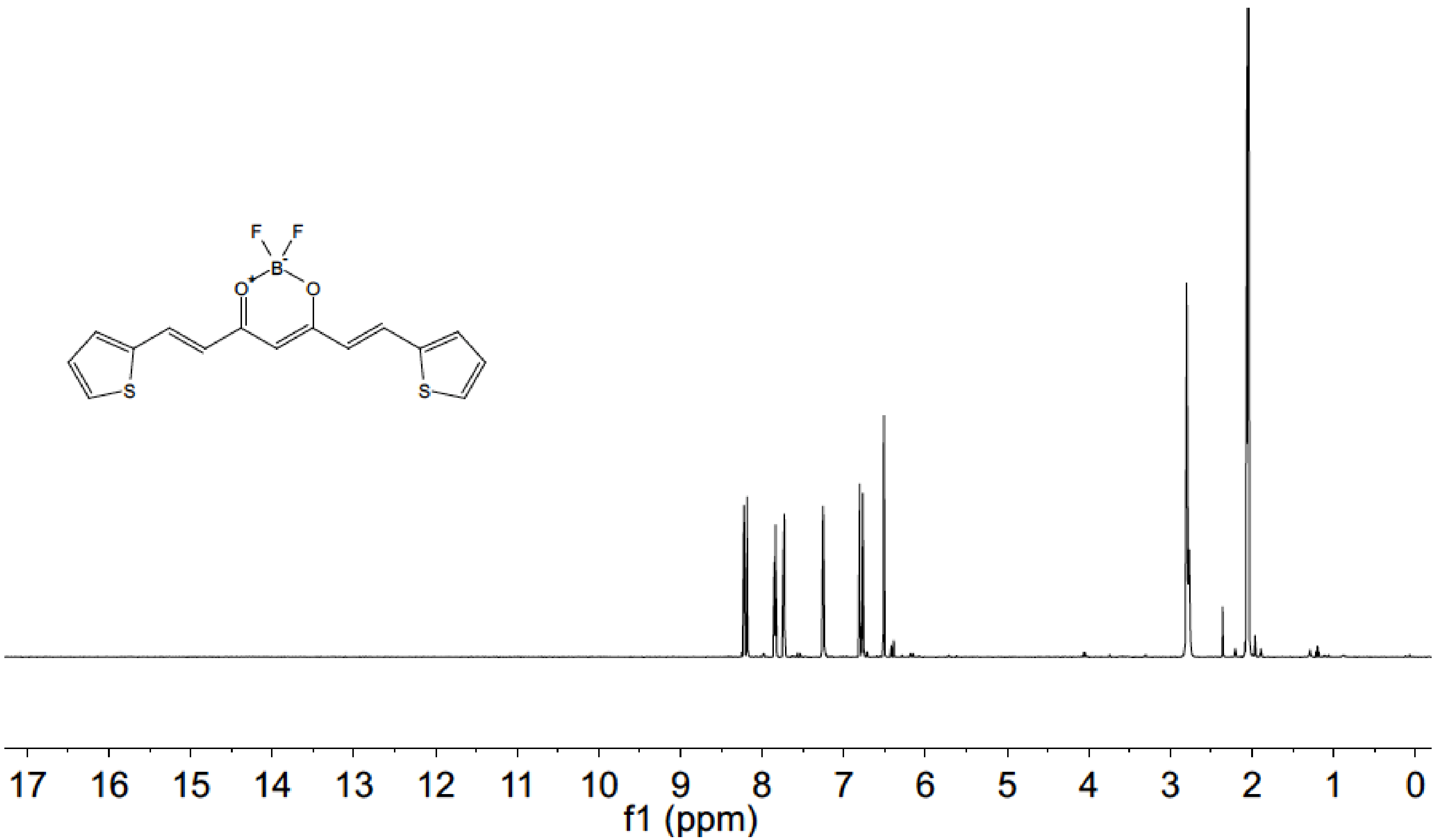

Figure S22. ^{1}H NMR spectrum of compound **4** (Acetone-*d6*-400MHz).

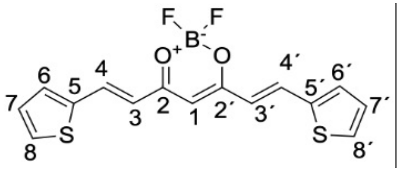

	^{13}C	^{1}H
1	102.81	6.51 (s)
2,2´	180.71	-
3,3´	120.67	6.79 (d, J = 15.38)
4,4´	139.92	8.20 (d, J = 15.38)
5,5´	140.92	-
6,6´	135.63	7.73 (d, J= 3.70)
7,7´	130.14	7.26 (dd, J = 5.05, 3.69)
8,8´	133.32	7.84 (d, J = 5.10)

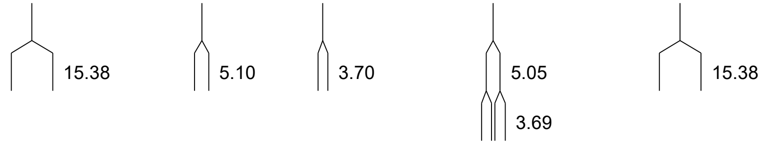

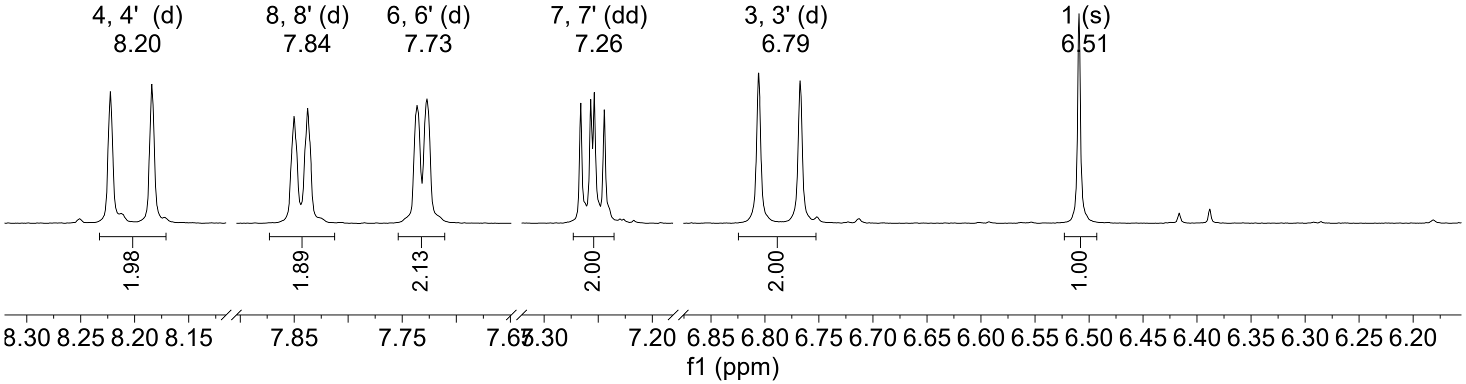

Figure S23. ^{1}H NMR spectrum of compound **4** (Acetone-*d6*-400MHz, expansion).

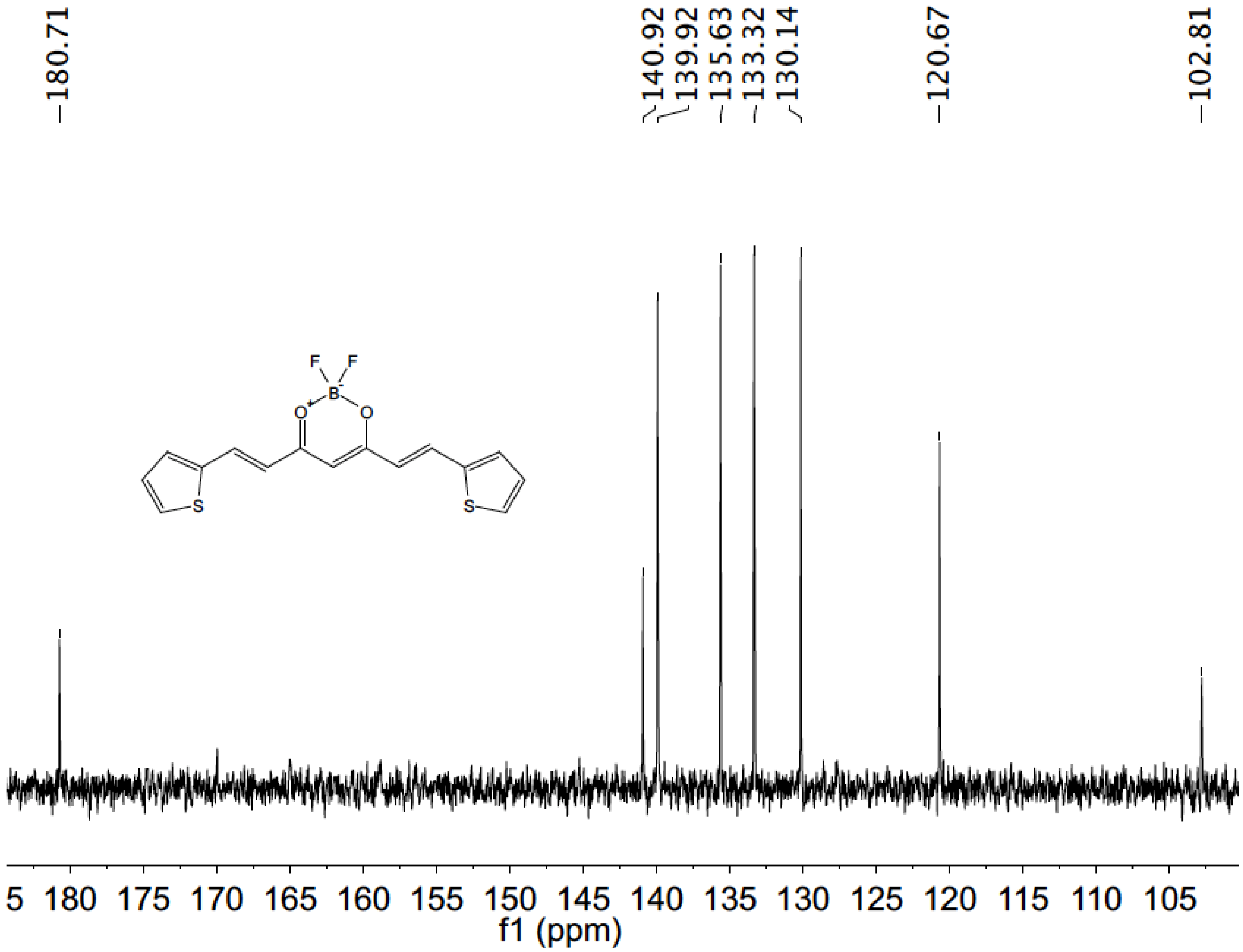

Figure S24. ^{13}C NMR spectrum of compound **4** (Acetone-*d6*-400MHz -100MHz).

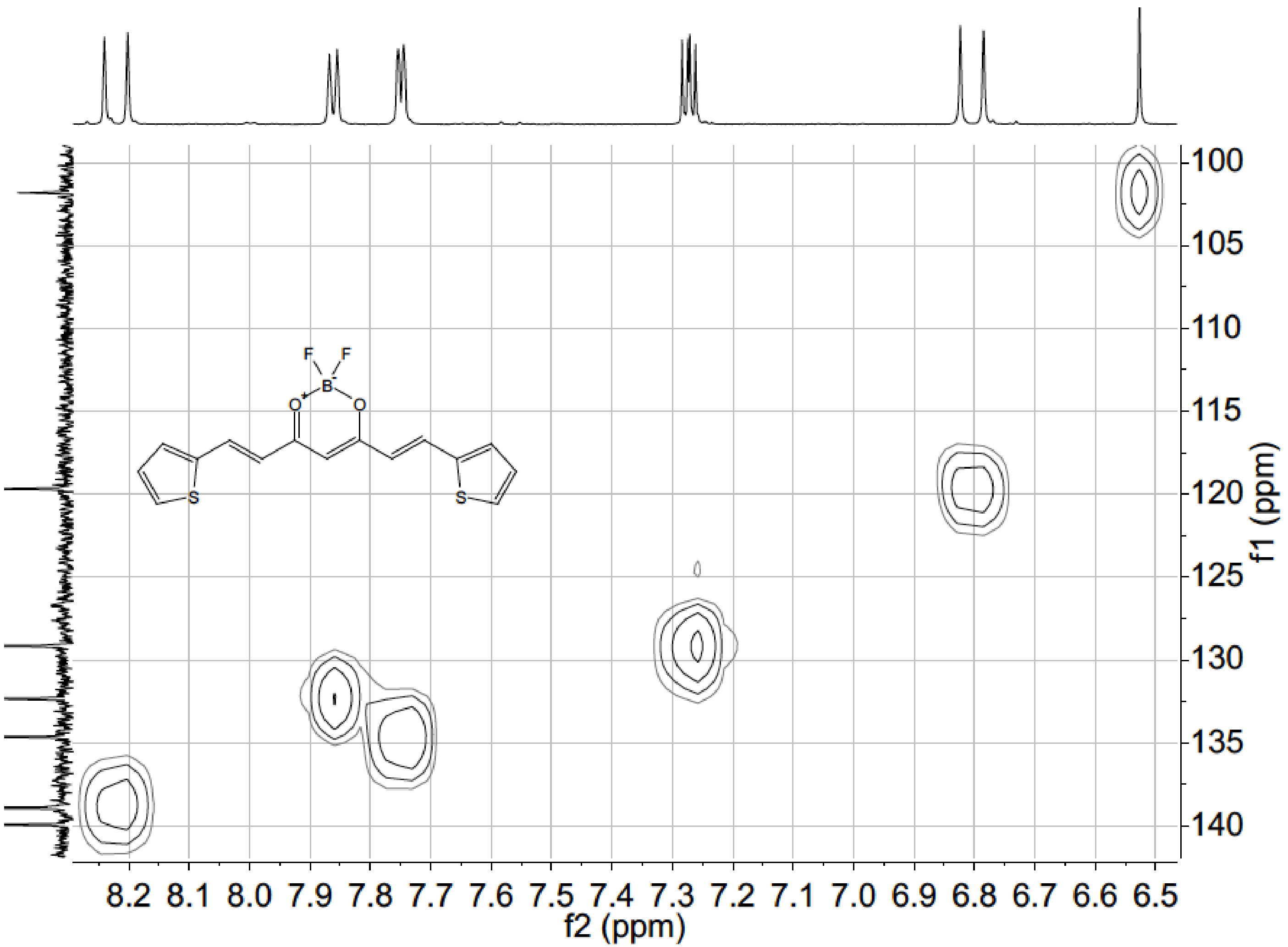

Figure S25. HSQC NMR spectrum of compound **4** (Acetone-*d6*-400MHz 400MHz).

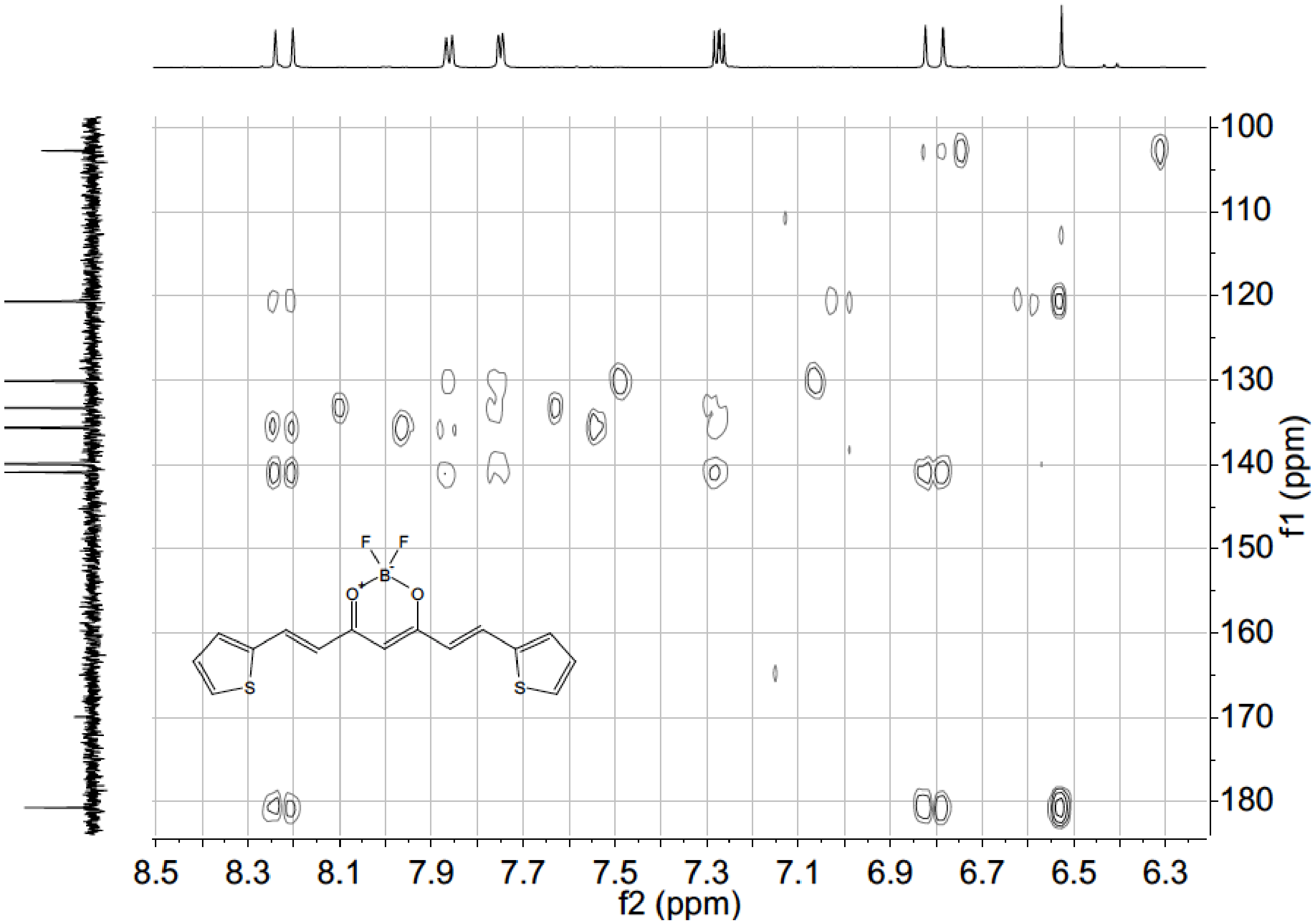

Figure S26. HMBC NMR spectrum of compound **4** (Acetone-*d6*-400MHz -400MHz).

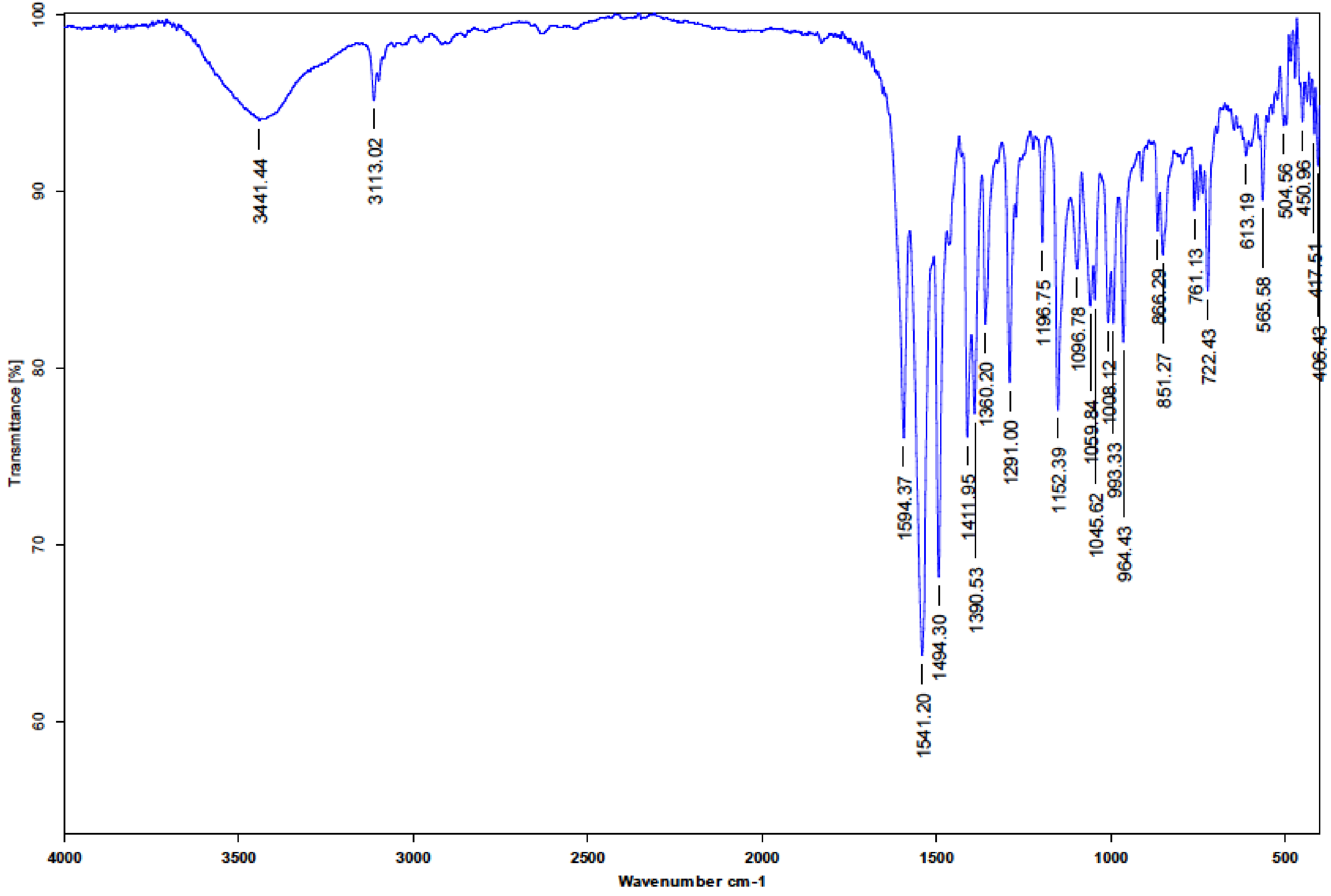

Figure S27. IR spectrum of compound **4.**

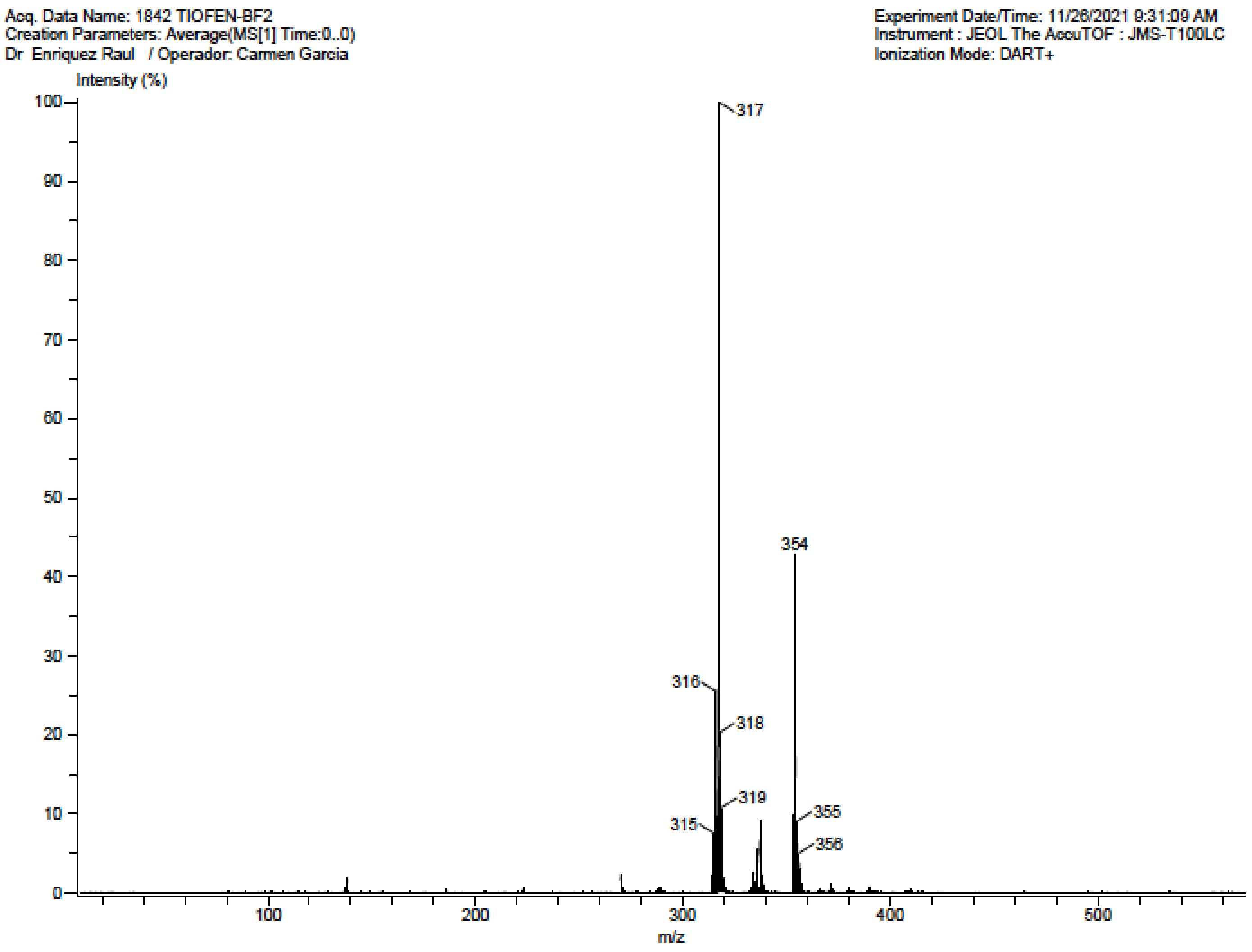

Figure S28. SM of compound **4.**

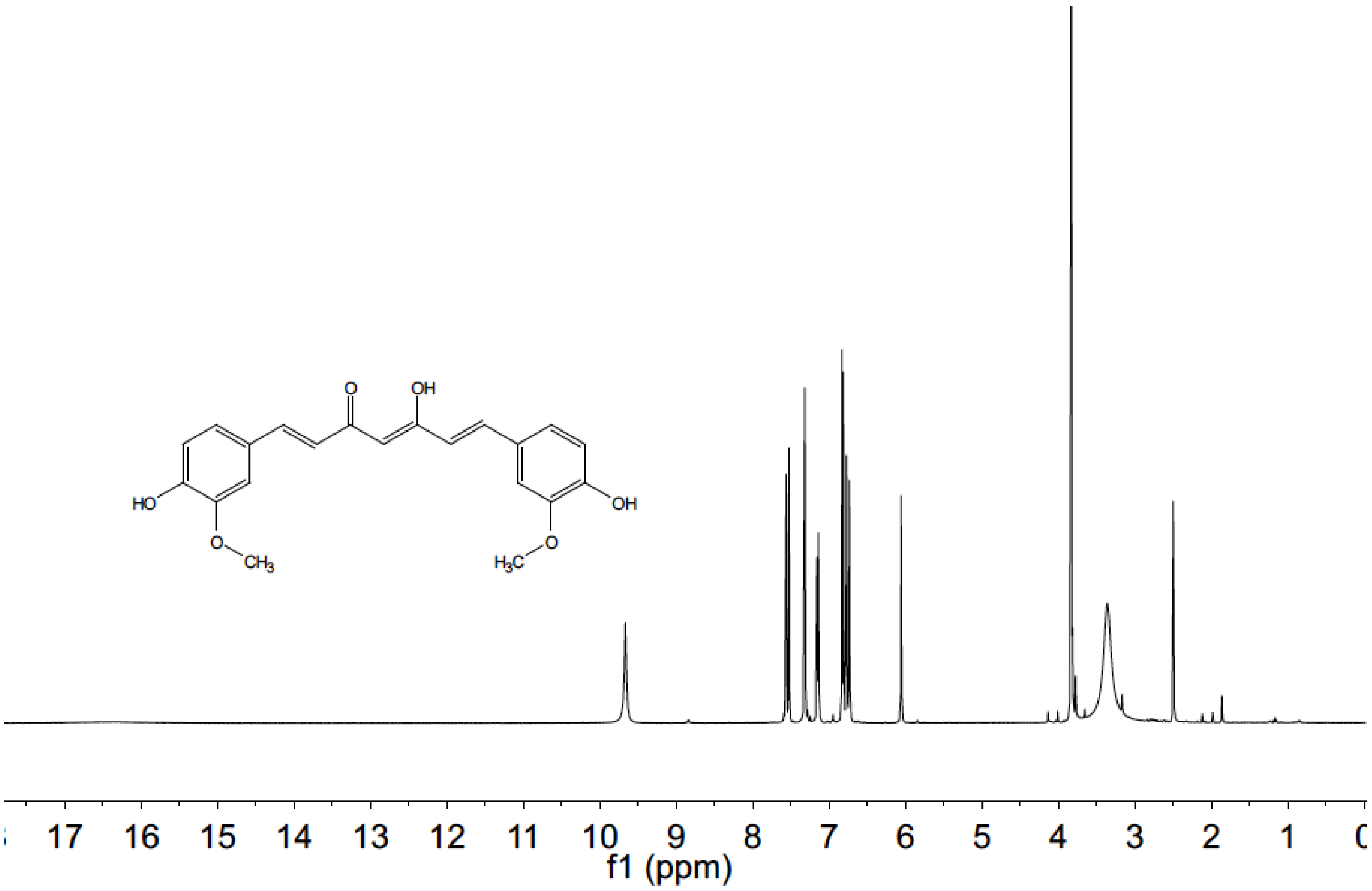

Figure 29. ^{1}H NMR spectrum of compound **5** (DMSO-*d6*-400MHz).

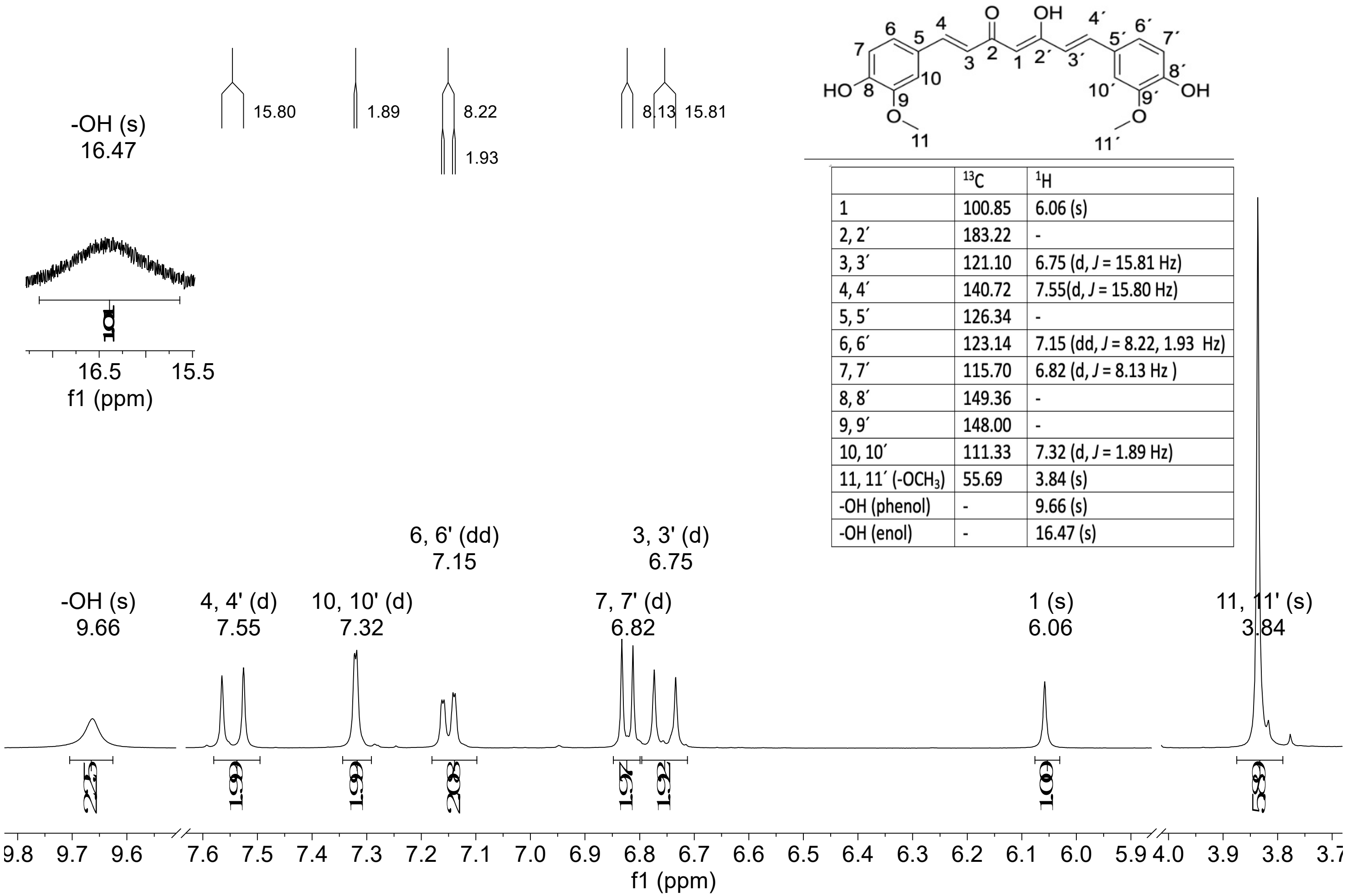

	^{13}C	^{1}H
1	100.85	6.06 (s)
2, 2´	183.22	-
3, 3´	121.10	6.75 (d, *J* = 15.81 Hz)
4, 4´	140.72	7.55(d, *J* = 15.80 Hz)
5, 5´	126.34	-
6, 6´	123.14	7.15 (dd, *J* = 8.22, 1.93 Hz)
7, 7´	115.70	6.82 (d, *J* = 8.13 Hz)
8, 8´	149.36	-
9, 9´	148.00	-
10, 10´	111.33	7.32 (d, *J* = 1.89 Hz)
11, 11´ (-OCH$_3$)	55.69	3.84 (s)
-OH (phenol)	-	9.66 (s)
-OH (enol)	-	16.47 (s)

Figure S30. ^{1}H NMR spectrum of compound **5** (DMSO-*d6*-400MHz, expansion).

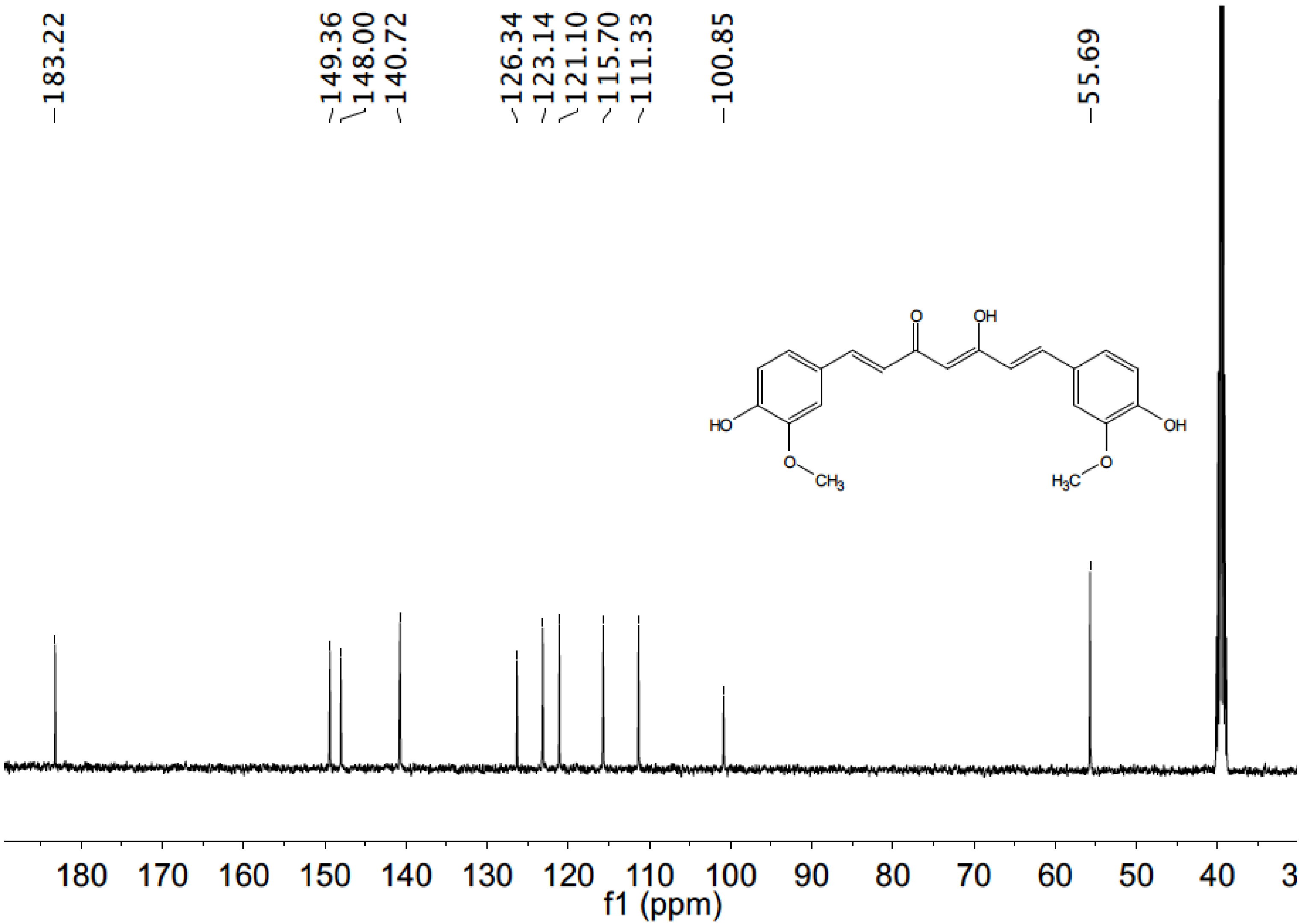

Figure S31. ^{13}C NMR spectrum of compound **5** (DMSO-*d6*-100MHz).

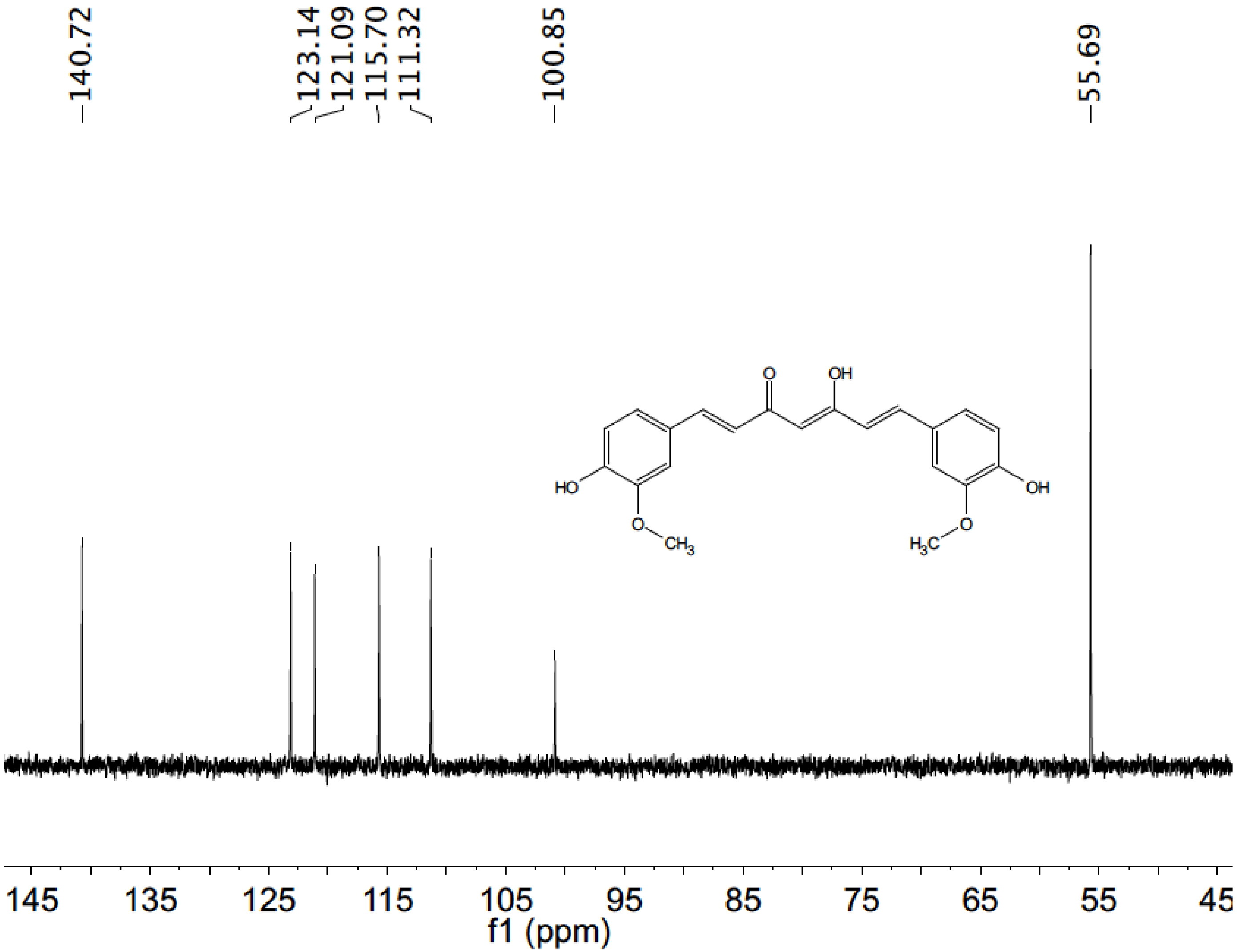

Figure S32. DEPT spectrum of compound **5** (DMSO-*d6*-100MHz).

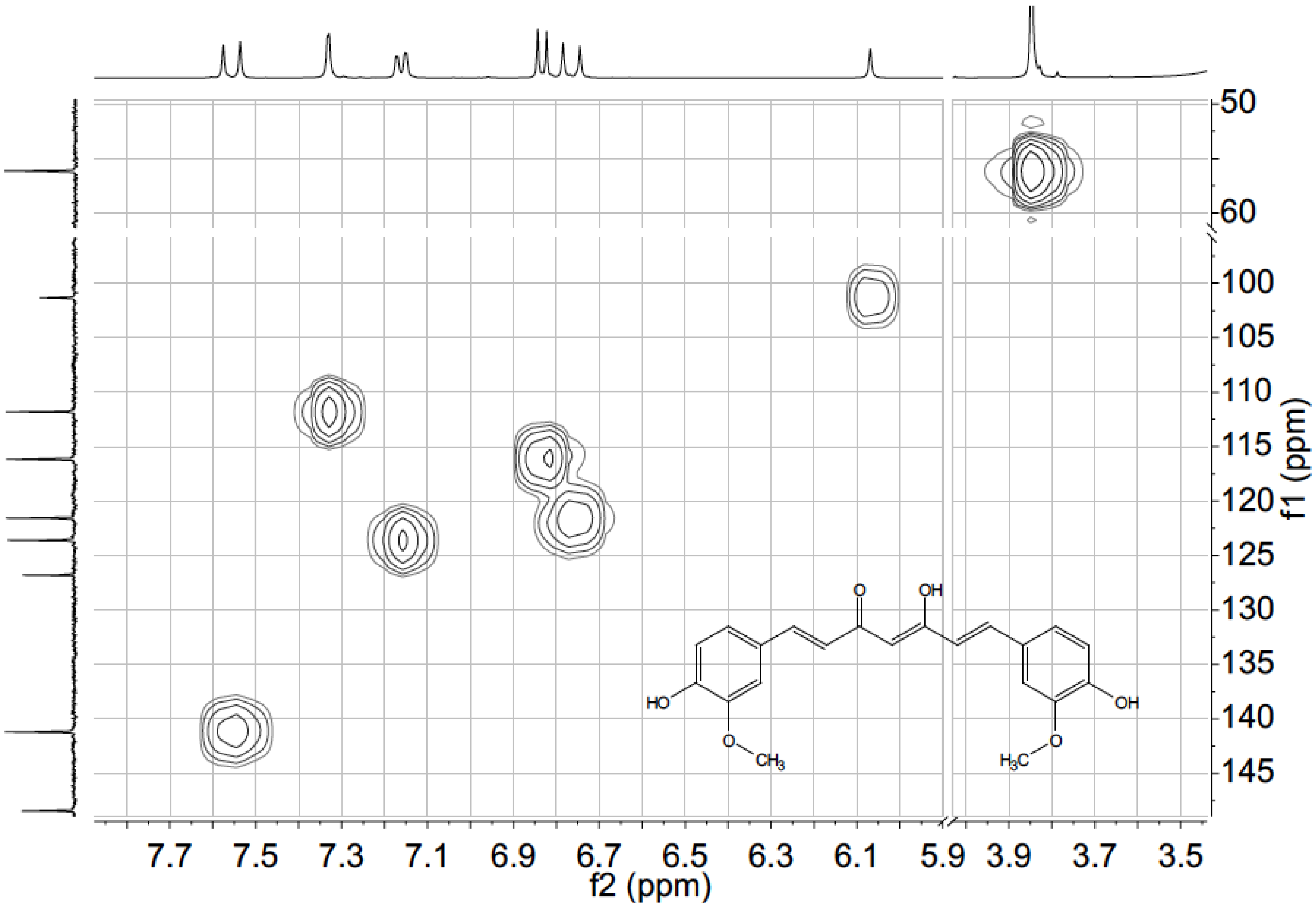

Figure S33. HSQC NMR spectrum of compound **5** (DMSO-*d6*-400MHz).

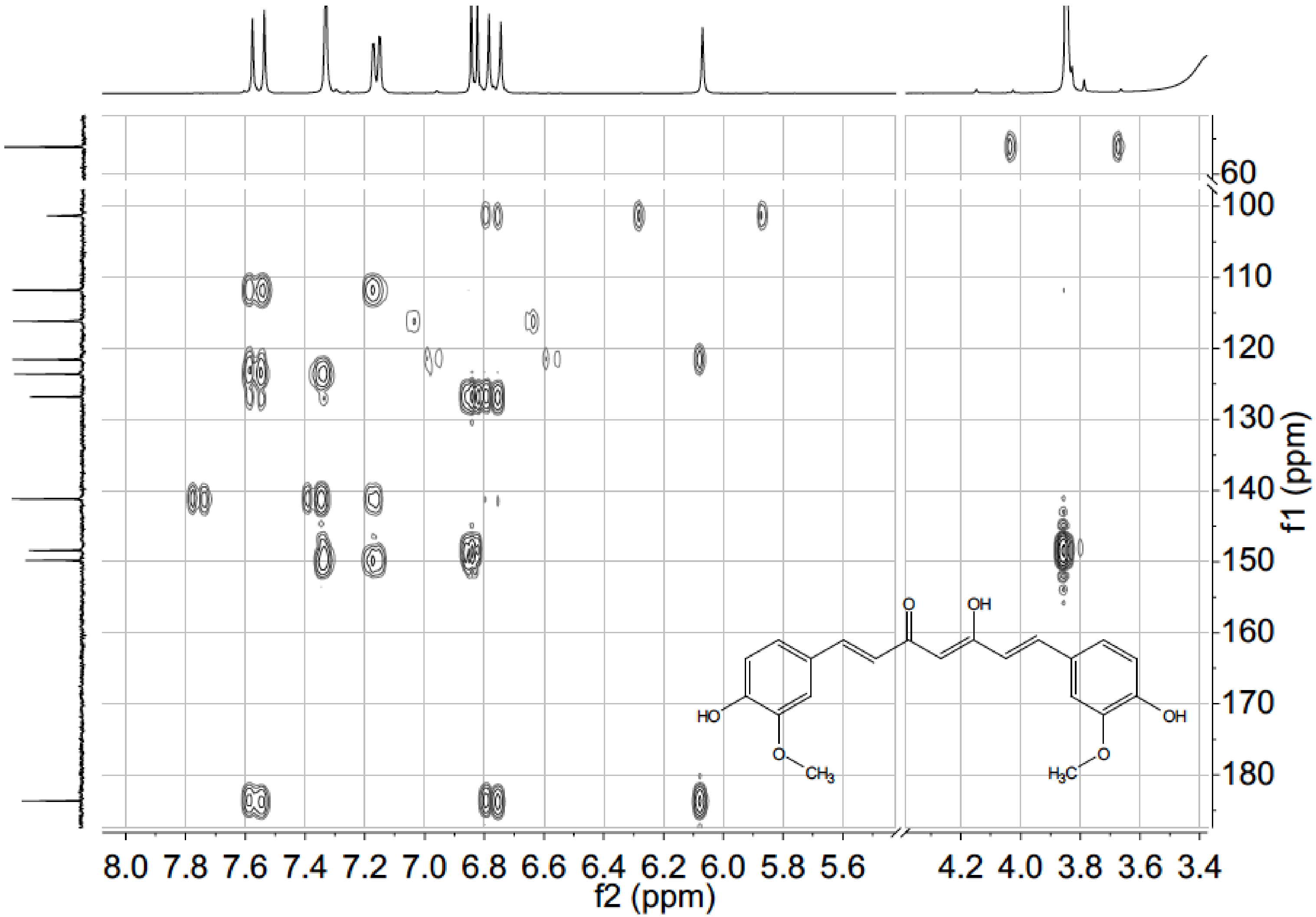

Figure S34. HMBC NMR spectrum of compound **5** (DMSO-*d6*-400MHz).

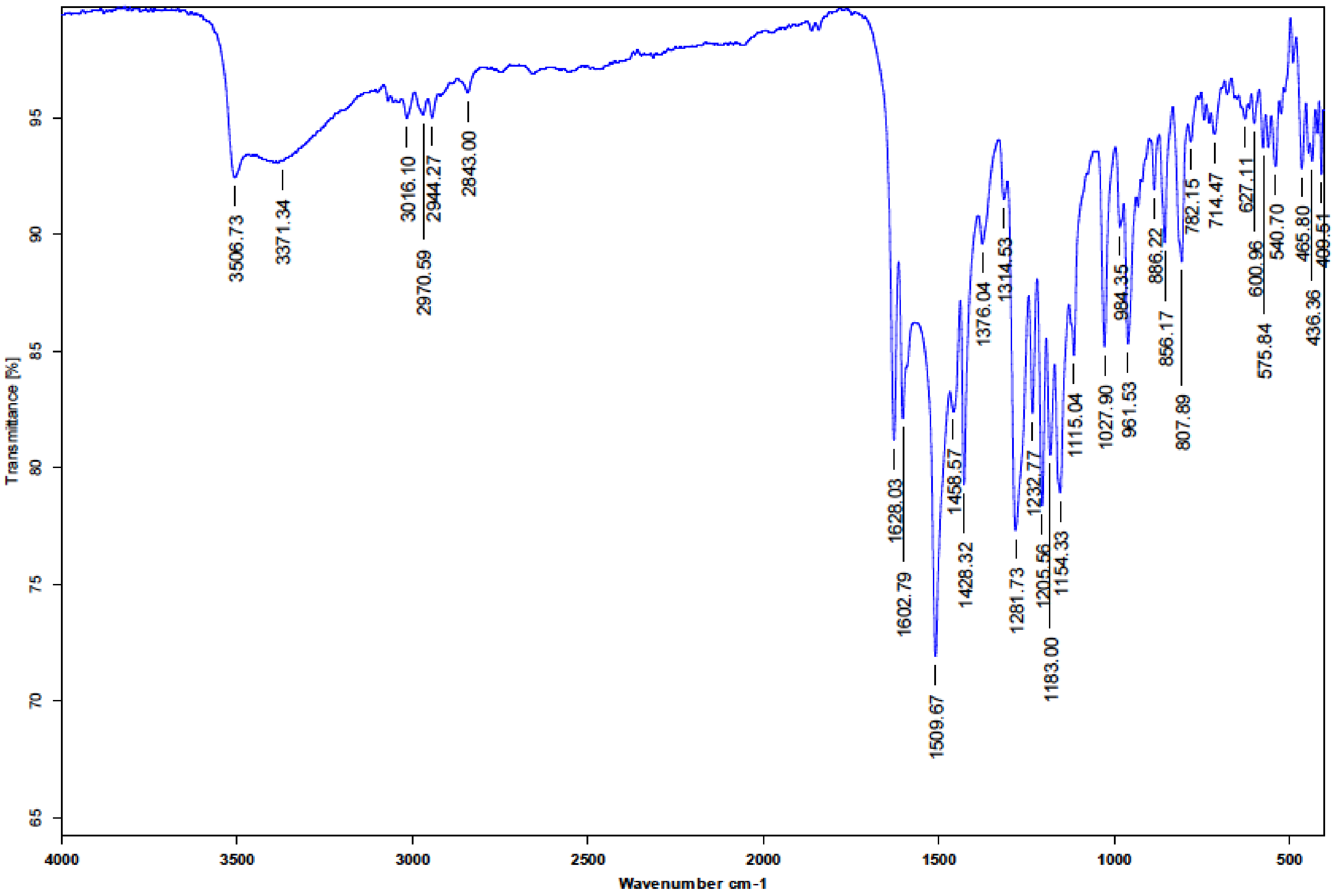

Figure S35. IR spectrum of compound **5**.

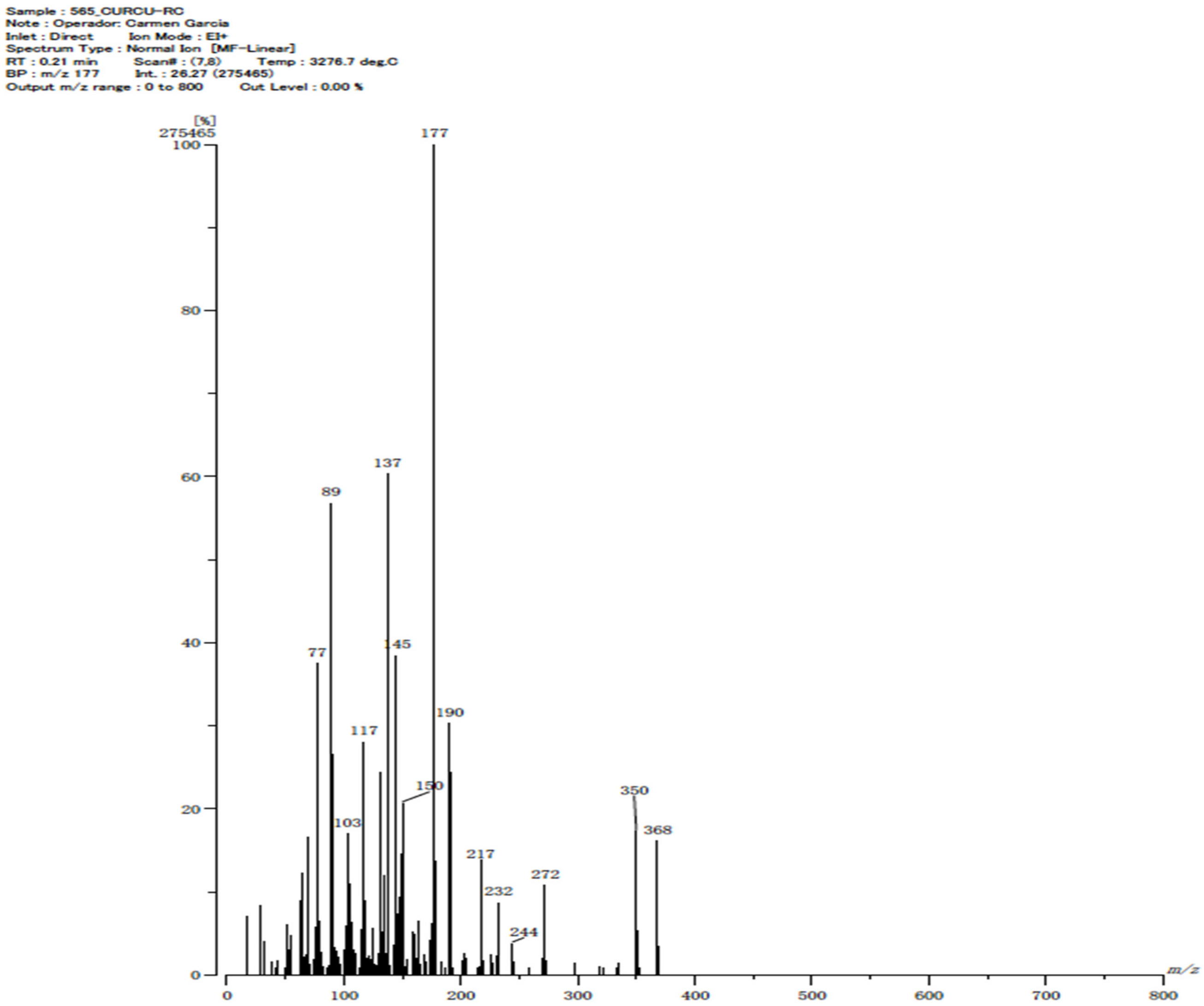

Figure S36. SM of compound **5**.

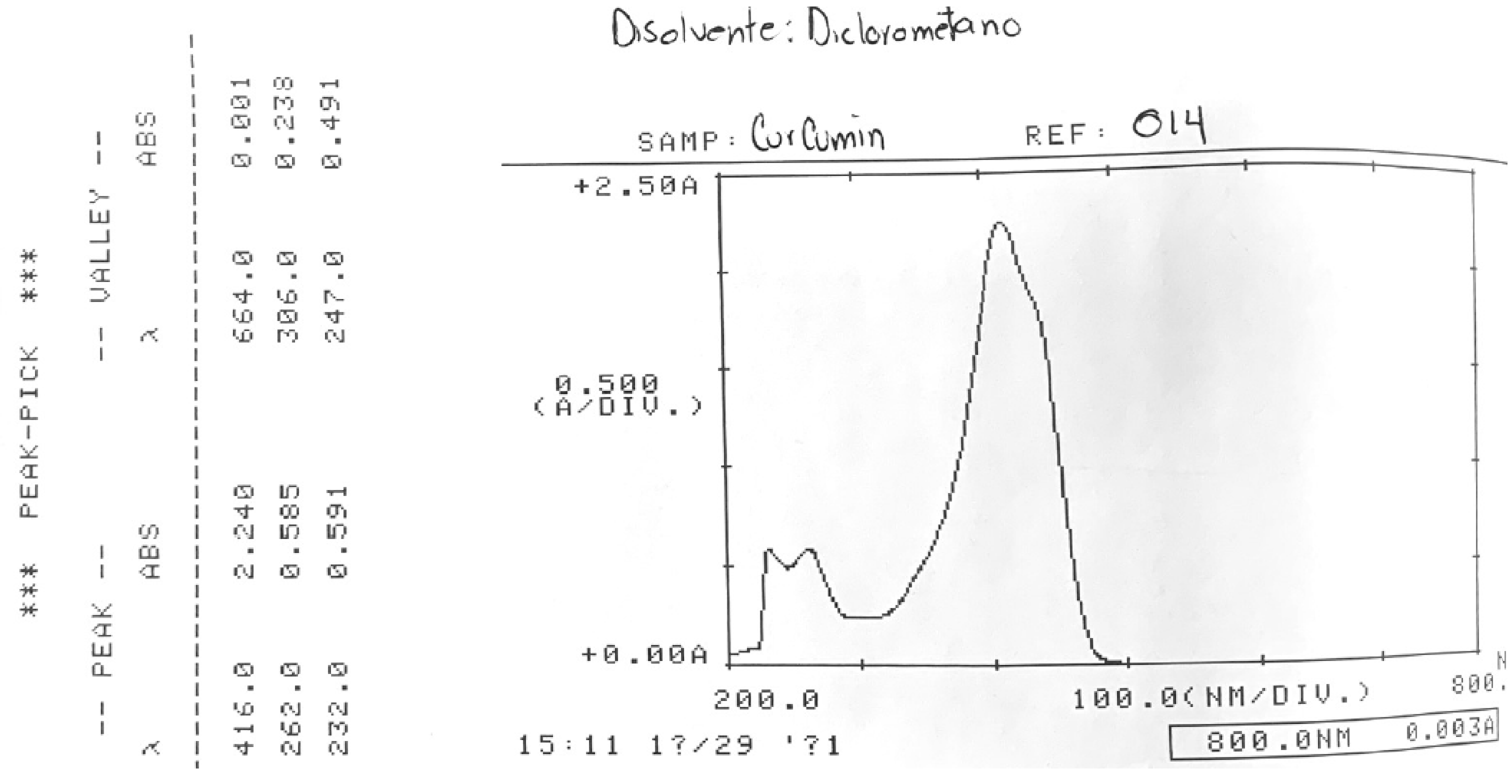

Figure S37. UV-spectrum of compound **5**.

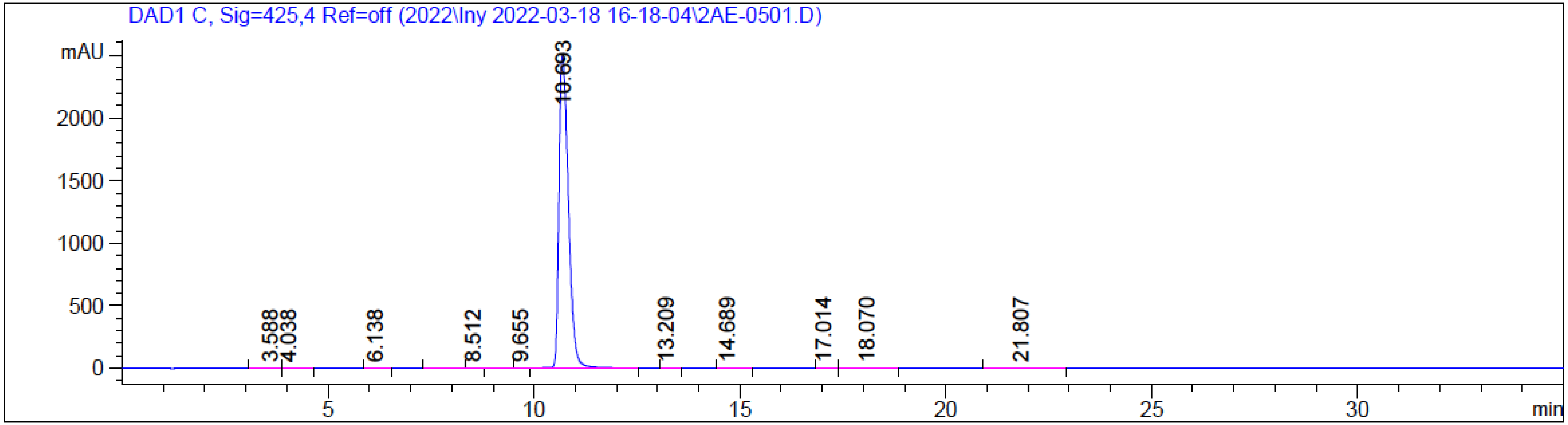

Peak #	RetTime [min]	Type	Width [min]	Area [mAU*s]	Height [mAU]	Area %
1	3.588	BV	0.2152	42.73438	3.01770	0.1064
2	4.038	VB	0.2323	21.86004	1.35418	0.0544
3	6.138	BB	0.2324	16.99524	1.11164	0.0423
4	8.512	BV E	0.2213	11.20550	7.72235e-1	0.0279
5	9.655	VV E	0.1915	11.97119	9.73339e-1	0.0298
6	10.693	VB R	0.2465	3.99483e4	2499.04443	99.4858
7	13.209	BB	0.1857	4.50077	3.33034e-1	0.0112
8	14.689	BB	0.2215	45.94841	3.09032	0.1144
9	17.014	BB	0.2013	7.72818	5.45482e-1	0.0192
10	18.070	VB R	0.2754	18.74504	9.61558e-1	0.0467
11	21.807	BB	0.4521	24.78504	6.98205e-1	0.0617
Totals :				4.01548e4	2511.90213	

Figure S38. HPLC of compound **5** (425nm).

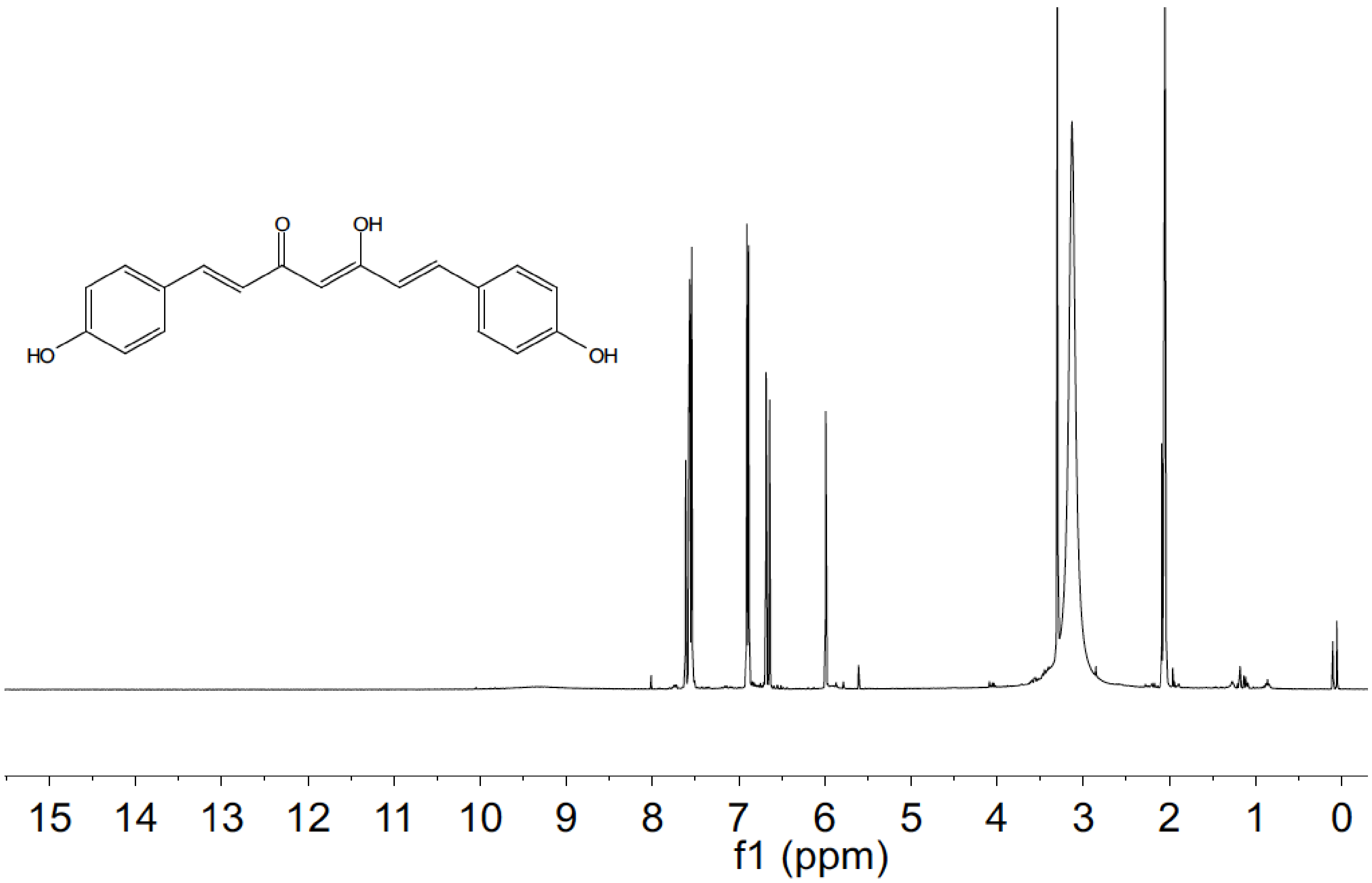

Figure 39. ^{1}H NMR spectrum of compound **6** (Acetone-*d6*-400MHz).

	^{13}C	^{1}H
1	101.78	5.99 (s)
2, 2′	184.57	-
3, 3′	121.98	6.66 (d, *J*= 15.82)
4, 4′	141.19	7.60 (d, *J*= 15.88)
5, 5′	127.58	-
6,6′, 10, 10′	131.03	7.55 (m)
7, 7′, 9, 9′	116.86	6.90 (m)
8, 8′	160.77	-
-OH	-	9.24 (s)

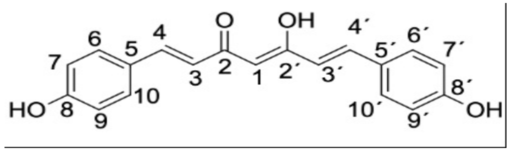

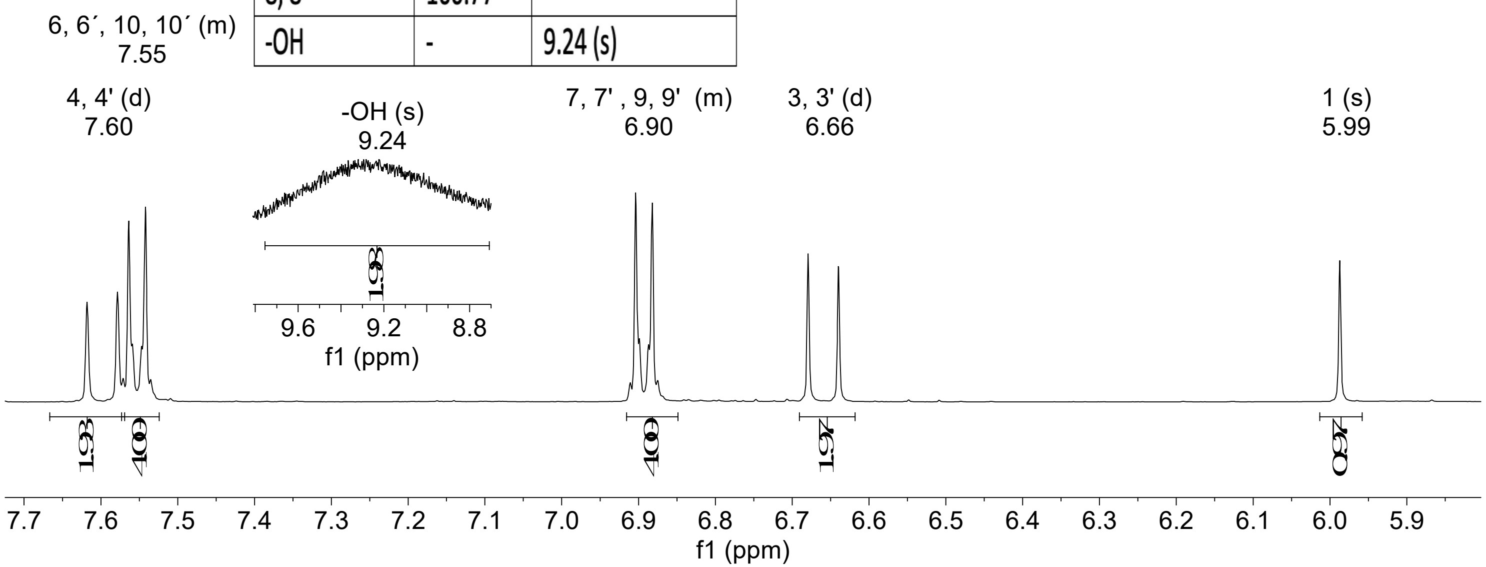

Figure S40. ^{1}H NMR spectrum of compound **6** (Acetone-*d6*-400MHz, expansion).

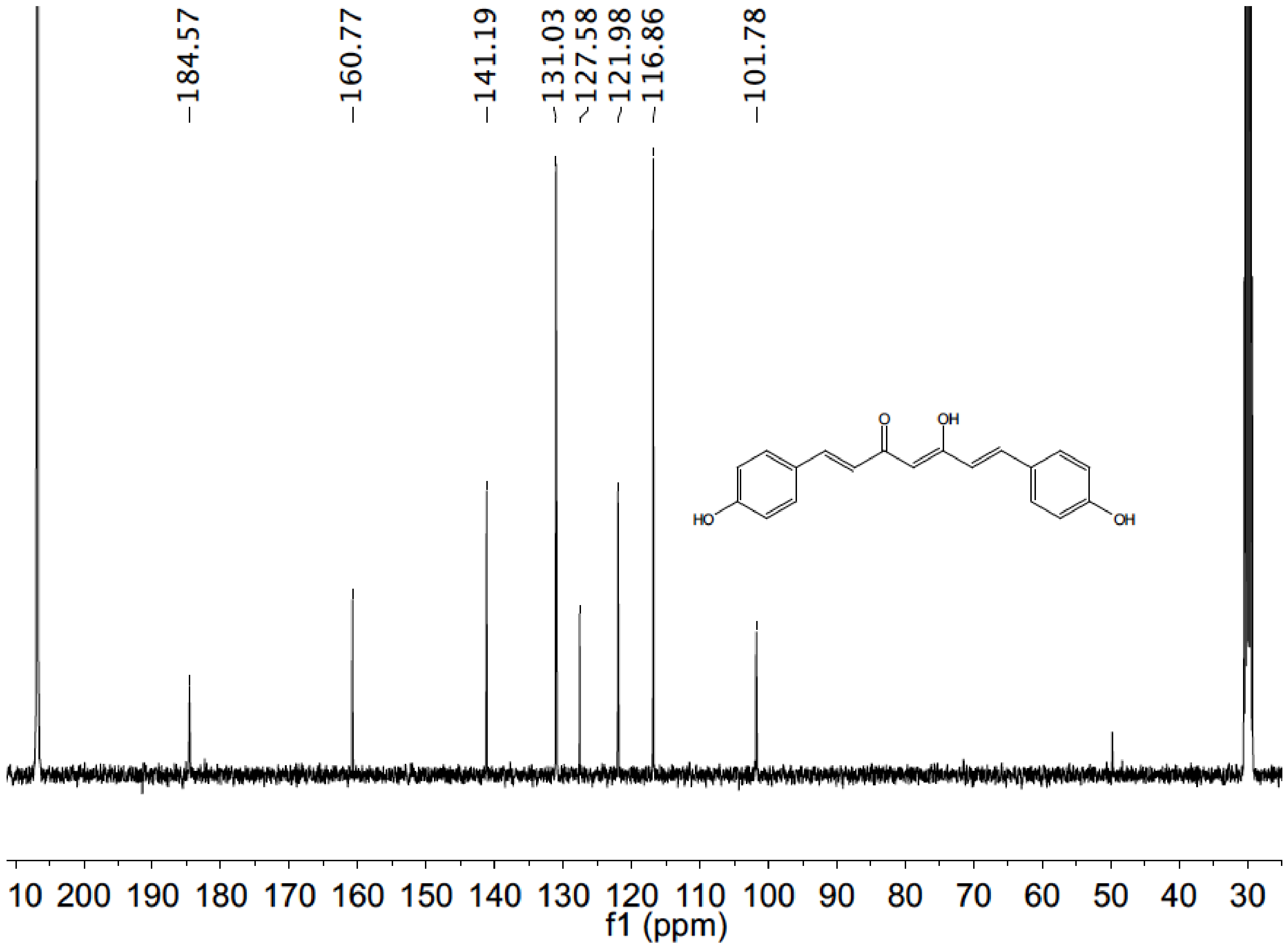

Figure S41. ^{13}C NMR spectrum of compound **6** (Acetone-*d6*-100MHz).

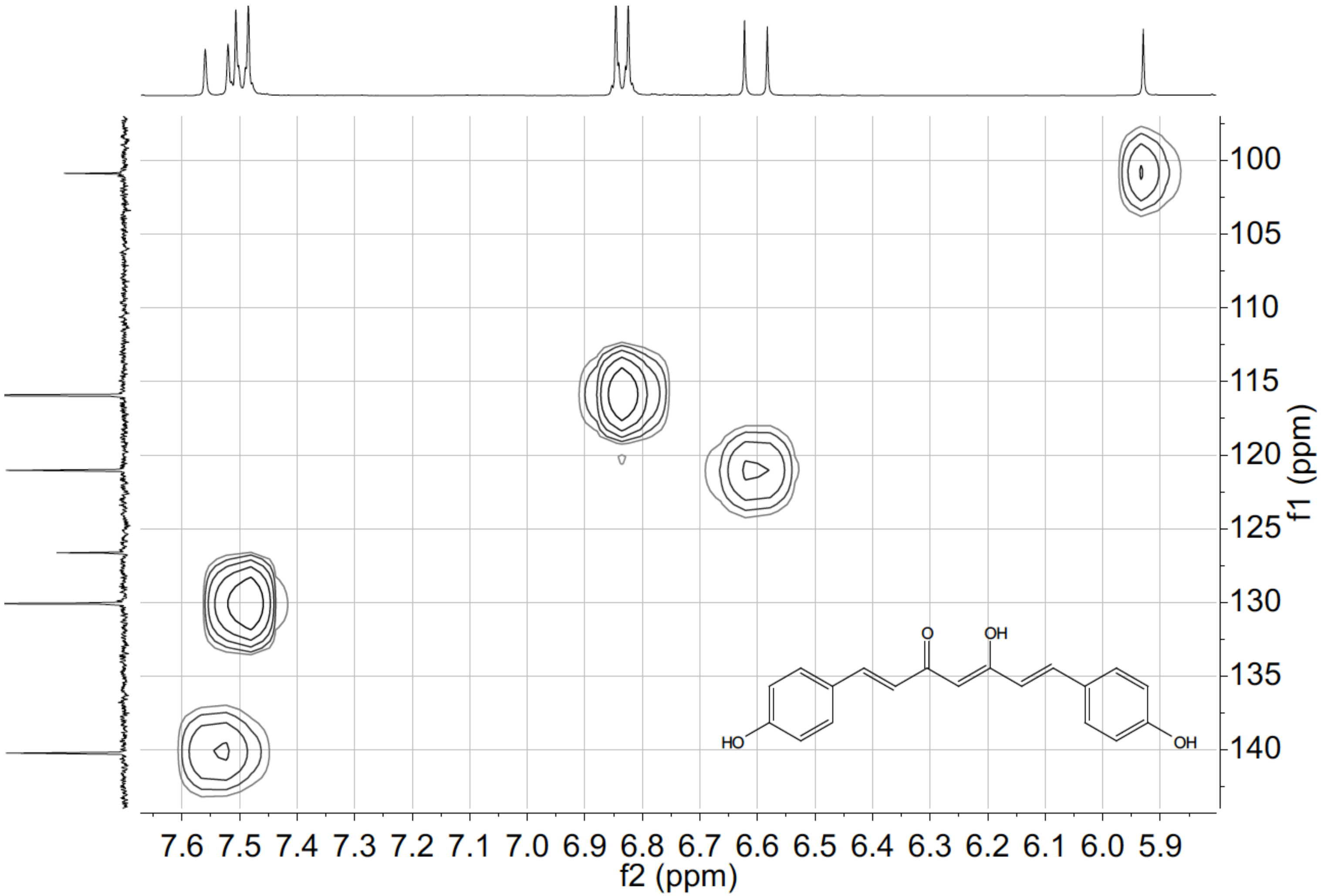

Figure S42. HSQC NMR spectrum of compound **6** (Acetone-*d6*-400MHz).

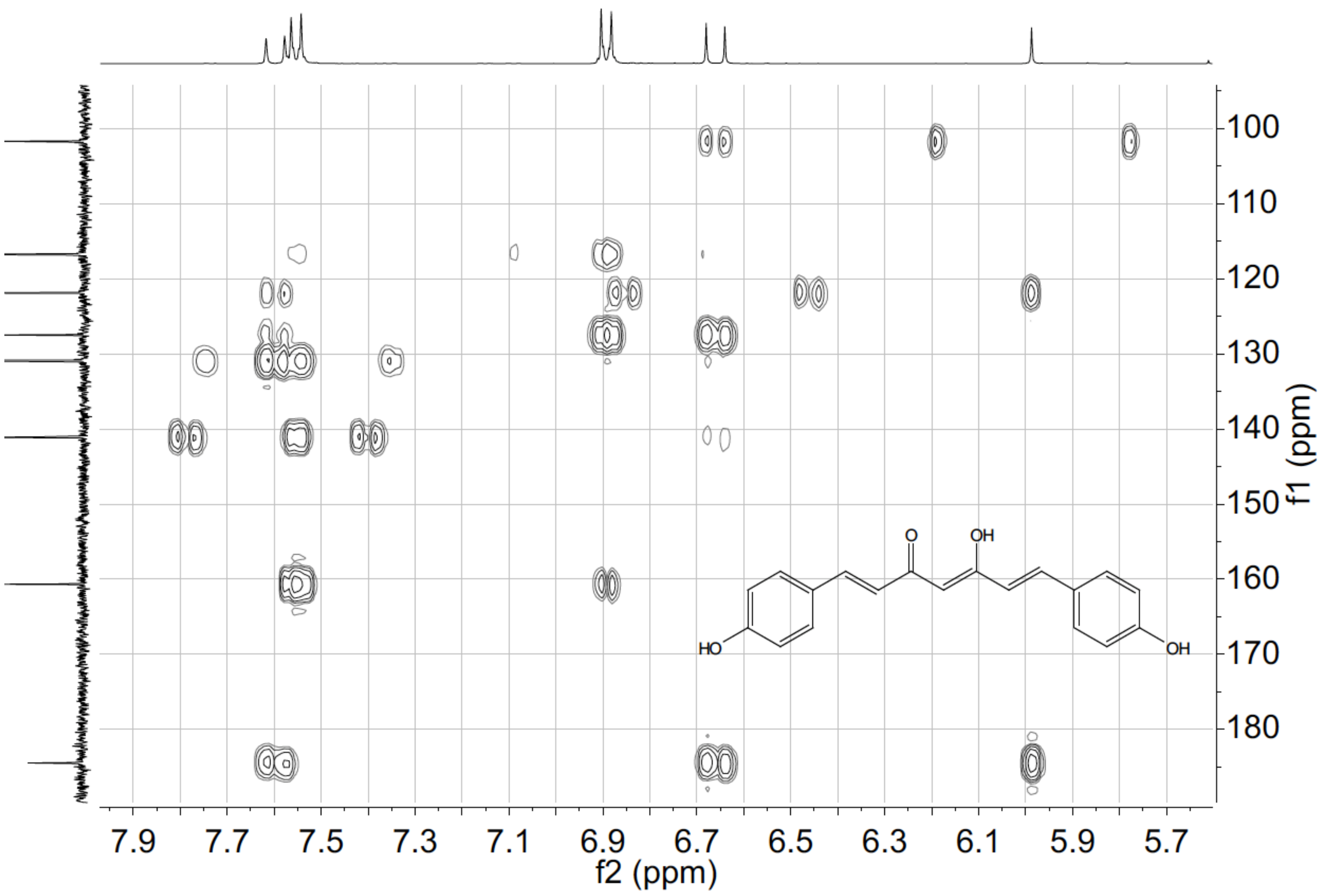

Figure S43. HMBC NMR spectrum of compound **6** (Acetone-*d6*-400MHz).

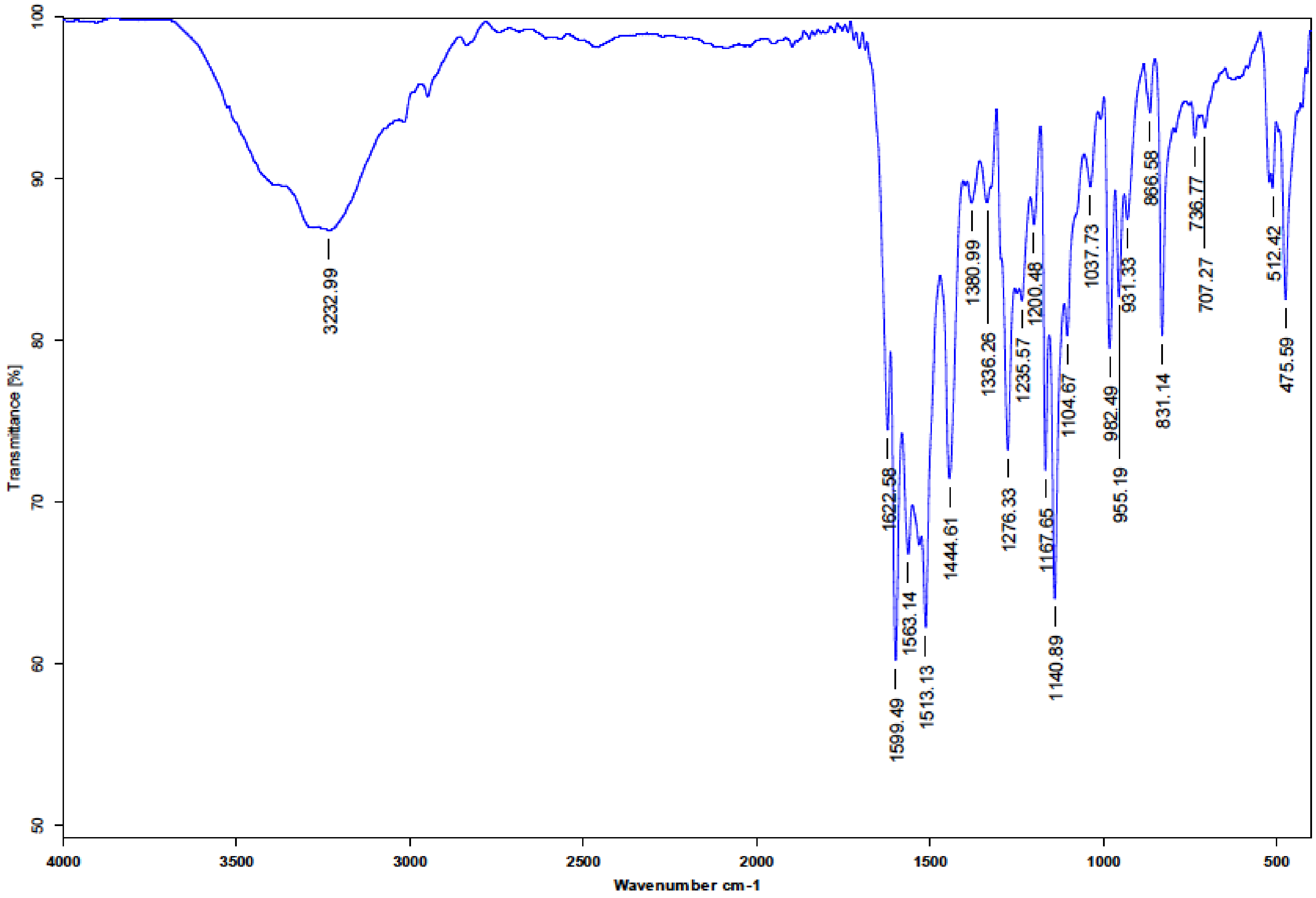

Figure S44. IR spectrum of compound **6**.

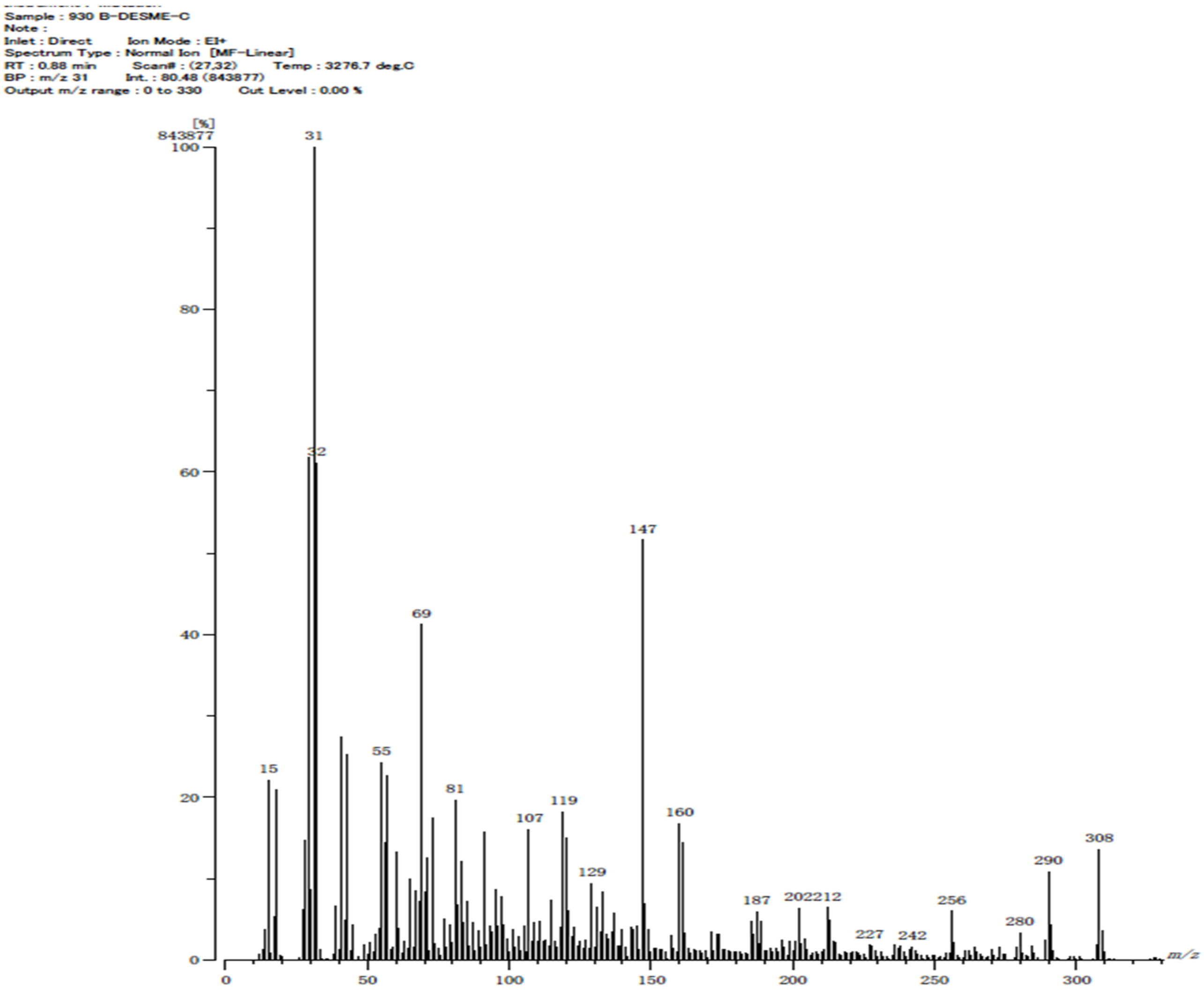

Figure S45. SM of compound **6**.

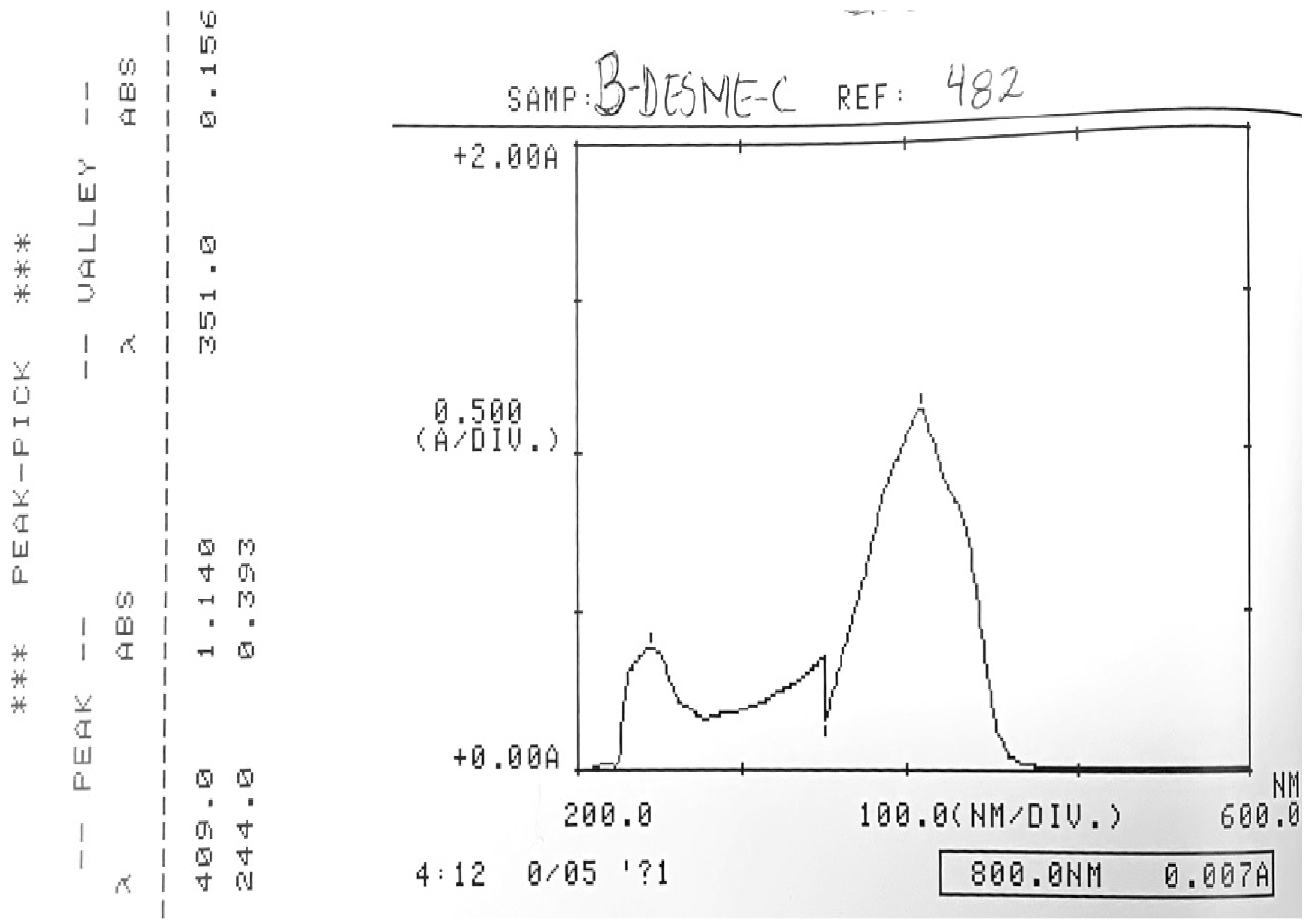

Figure S46. UV-spectrum of compound **6**.

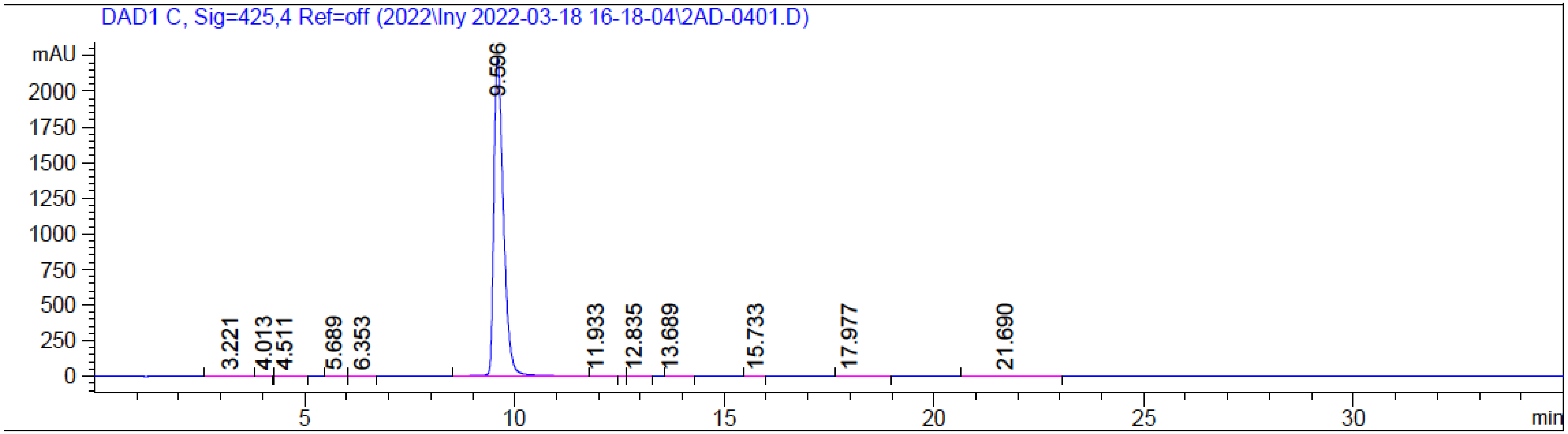

Signal 2: DAD1 C, Sig=425,4 Ref=off

Peak #	RetTime [min]	Type	Width [min]	Area [mAU*s]	Height [mAU]	Area %
1	3.221	VV R	0.2575	48.88575	2.62117	0.1351
2	4.013	VB E	0.1681	1.97942	1.53853e-1	5.472e-3
3	4.511	BV R	0.2440	9.65700	6.05775e-1	0.0267
4	5.689	BV	0.2274	11.49994	7.47740e-1	0.0318
5	6.353	VB	0.2731	8.18628	3.68578e-1	0.0226
6	9.596	BV R	0.2500	3.59731e4	2232.55737	99.4504
7	11.933	VB E	0.2525	3.43527	1.70917e-1	9.497e-3
8	12.835	BB	0.1994	6.59981	4.60051e-1	0.0182
9	13.689	BB	0.3211	1.85606	7.05078e-2	5.131e-3
10	15.733	BB	0.1821	5.40276	4.43434e-1	0.0149
11	17.977	BB	0.2585	19.91590	1.11436	0.0551
12	21.690	BB	0.5418	81.36864	1.91462	0.2250
Totals :				3.61718e4	2241.22838	

Figure S47. HPLC of compound **6** (425nm).

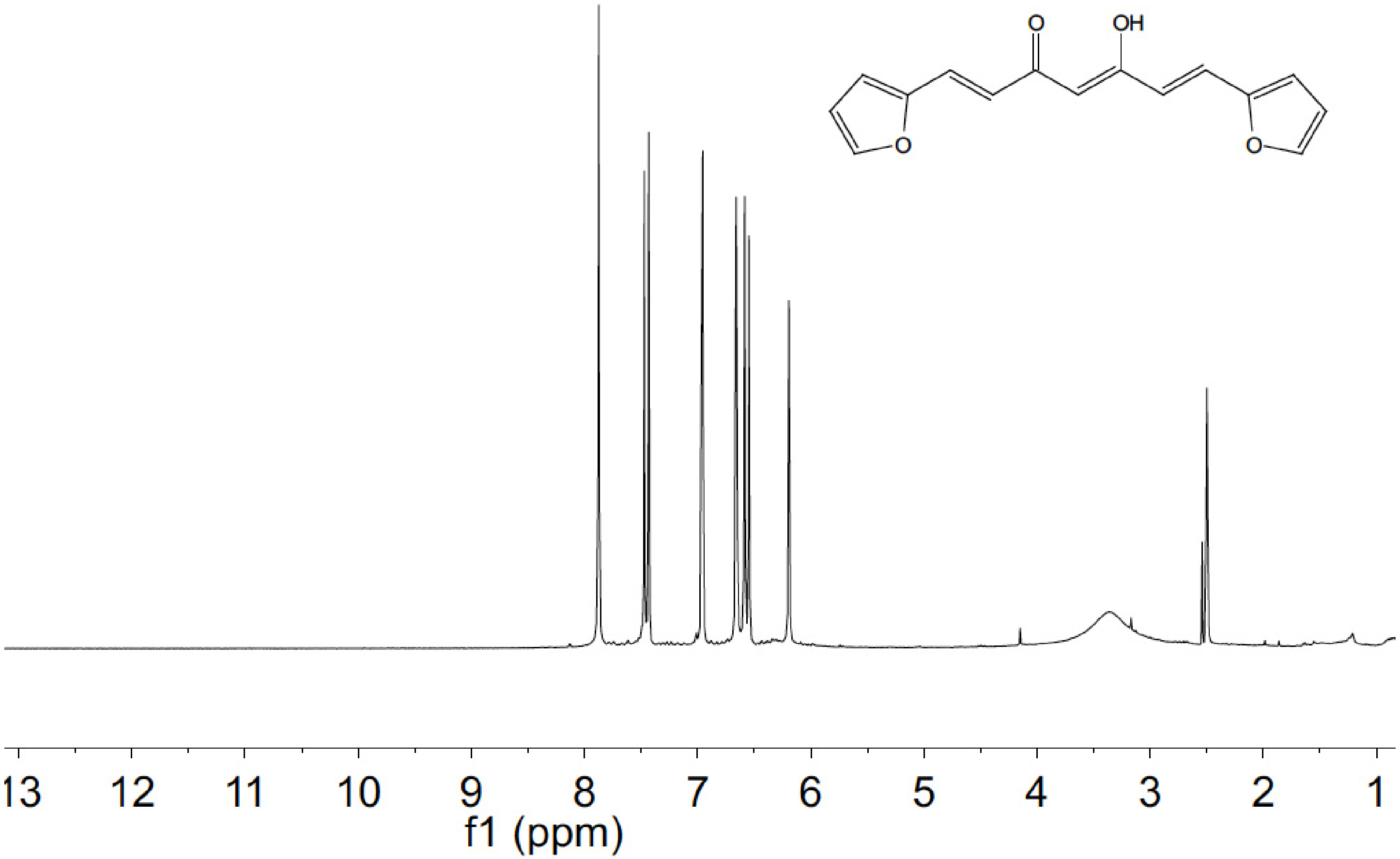

Figure 48. ^{1}H NMR spectrum of compound **7** (DMSO-*d6*-400MHz).

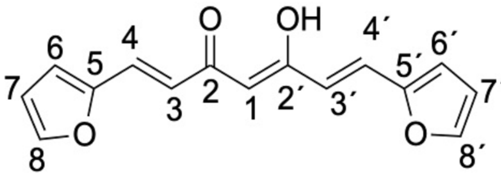

	^{13}C	^{1}H
1	101.98	6.19 (s)
2,2´	182.48	-
3,3´	121.18	6.57 (d, *J*= 15.71)
4,4´	126.94	7.45 (d, *J*= 15.71)
5,5´	151.02	-
6,6´	116.15	6.96 (d, *J*= 3.37)
7,7´	113.06	6.66 (dd, *J*= 3.43, 1.68)
8,8´	146.12	7.87 (d, *J*= 1.70)

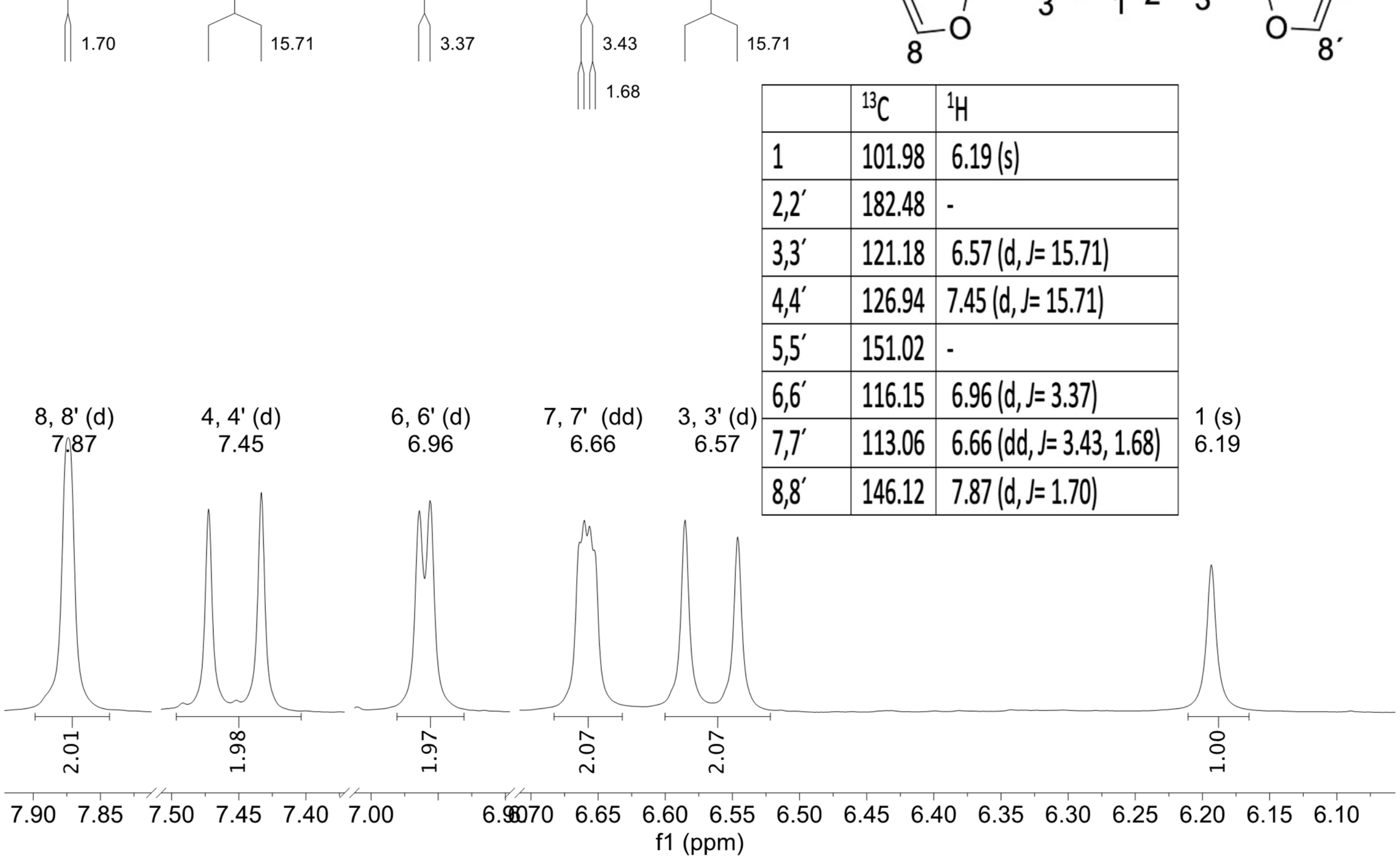

Figure S49. ^{1}H NMR spectrum of compound **7** (DMSO-*d6*-400MHz, expansion).

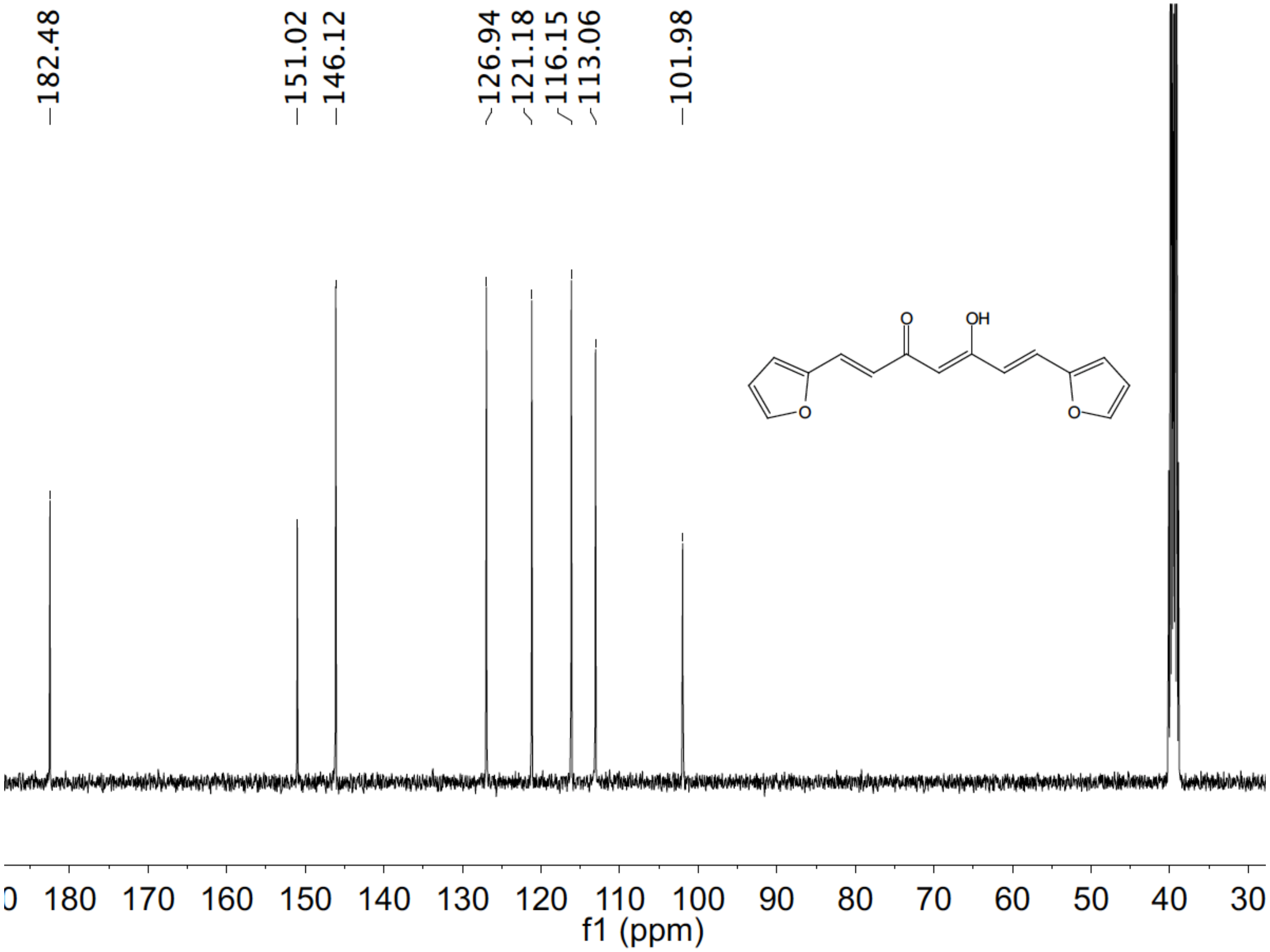

Figure S50. ^{13}C NMR spectrum of compound **7** (DMSO-*d6*-100MHz).

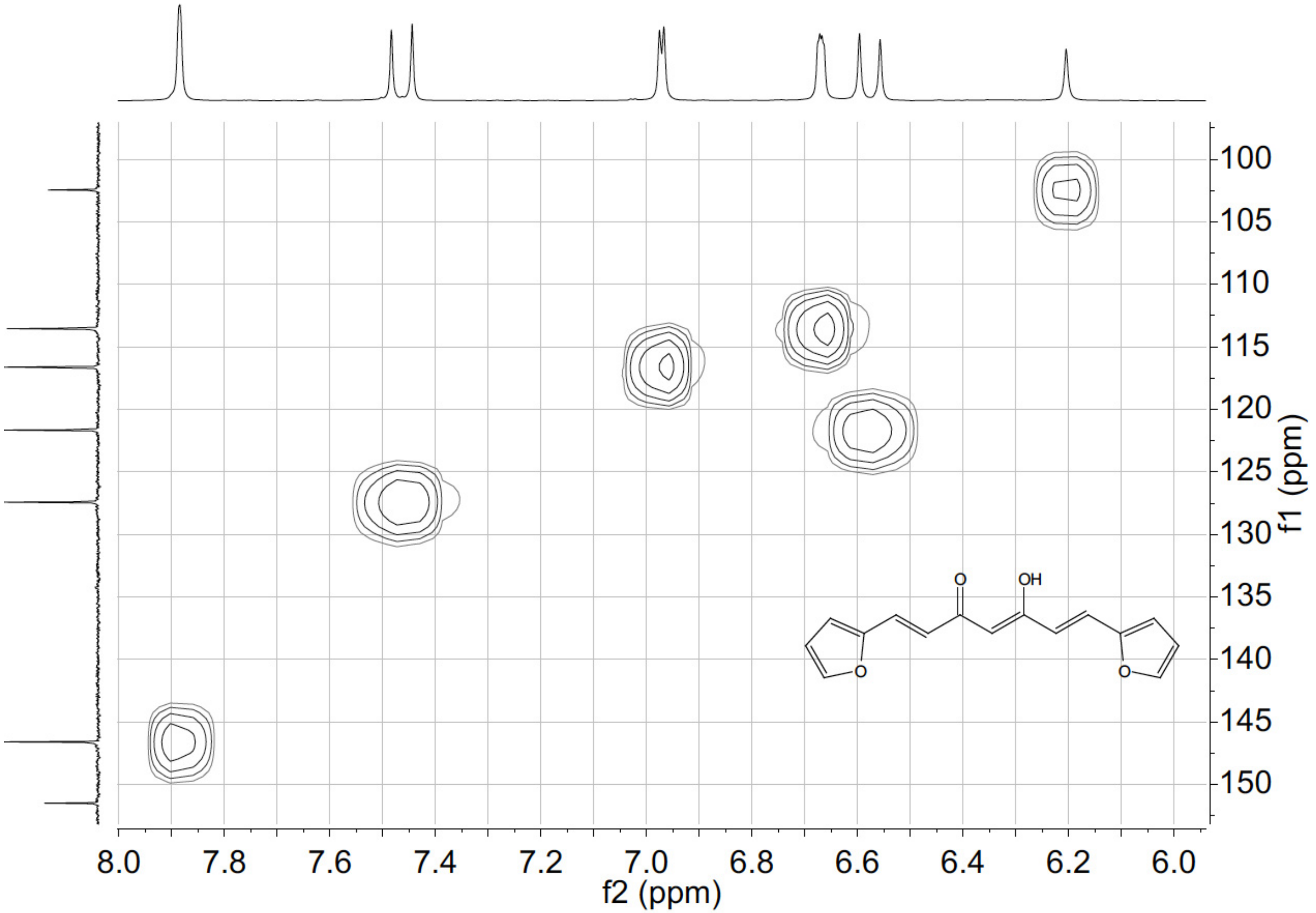

Figure S51. HSQC NMR spectrum of compound **7** (DMSO-*d6*-400MHz).

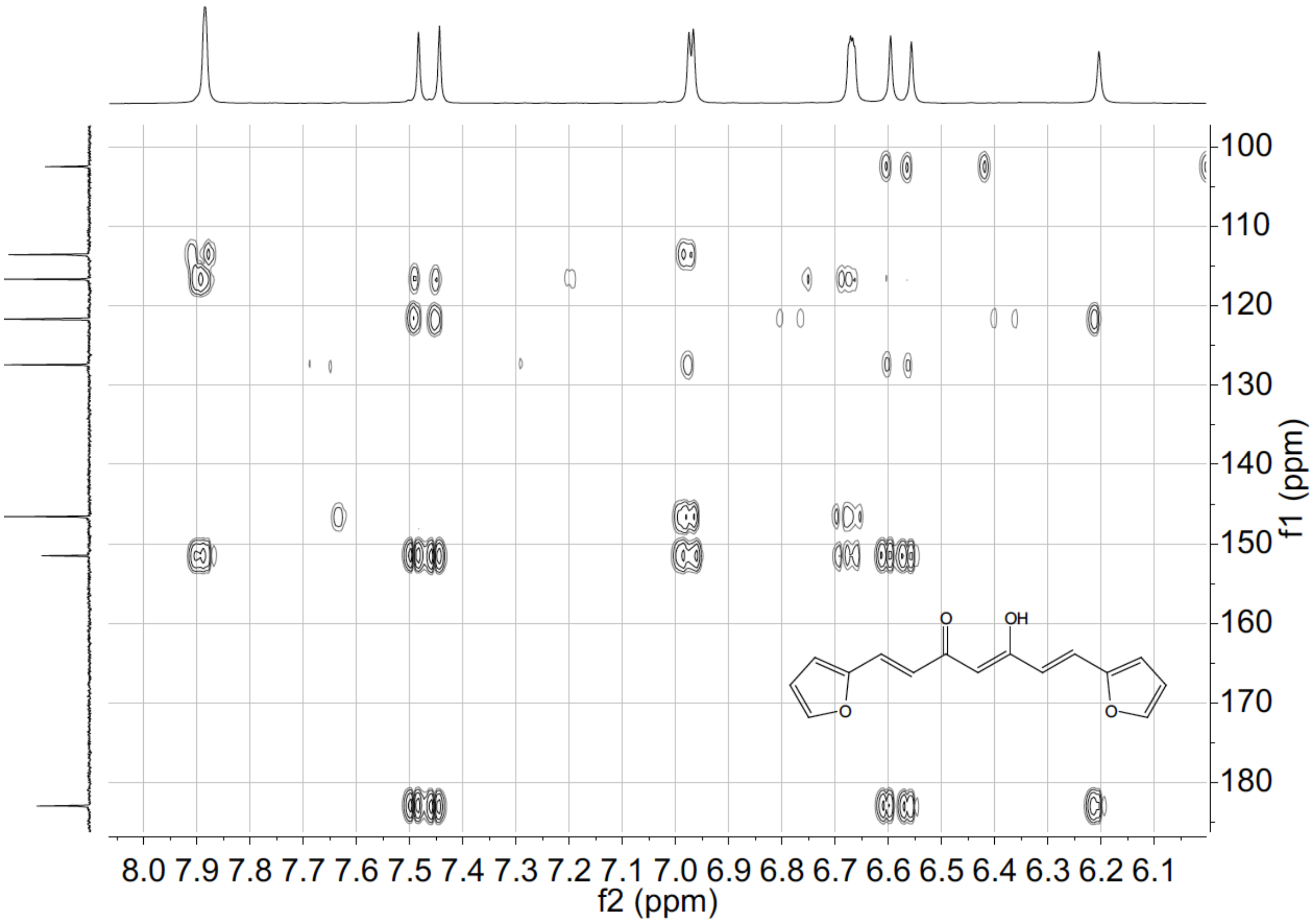

Figure S52. HMBC NMR spectrum of compound **7** (DMSO-*d6*-400MHz).

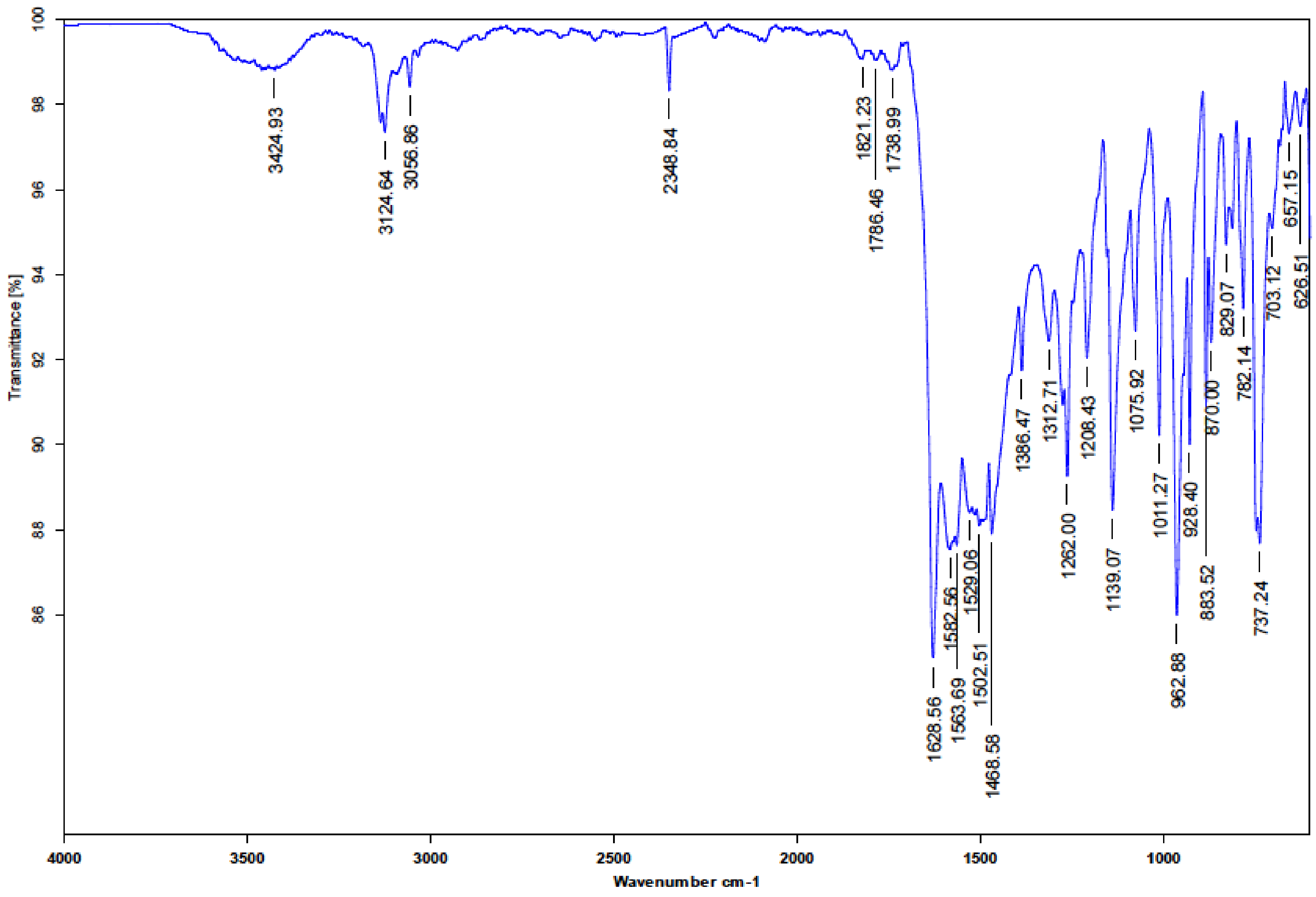

Figure S53. IR spectrum of compound **7**.

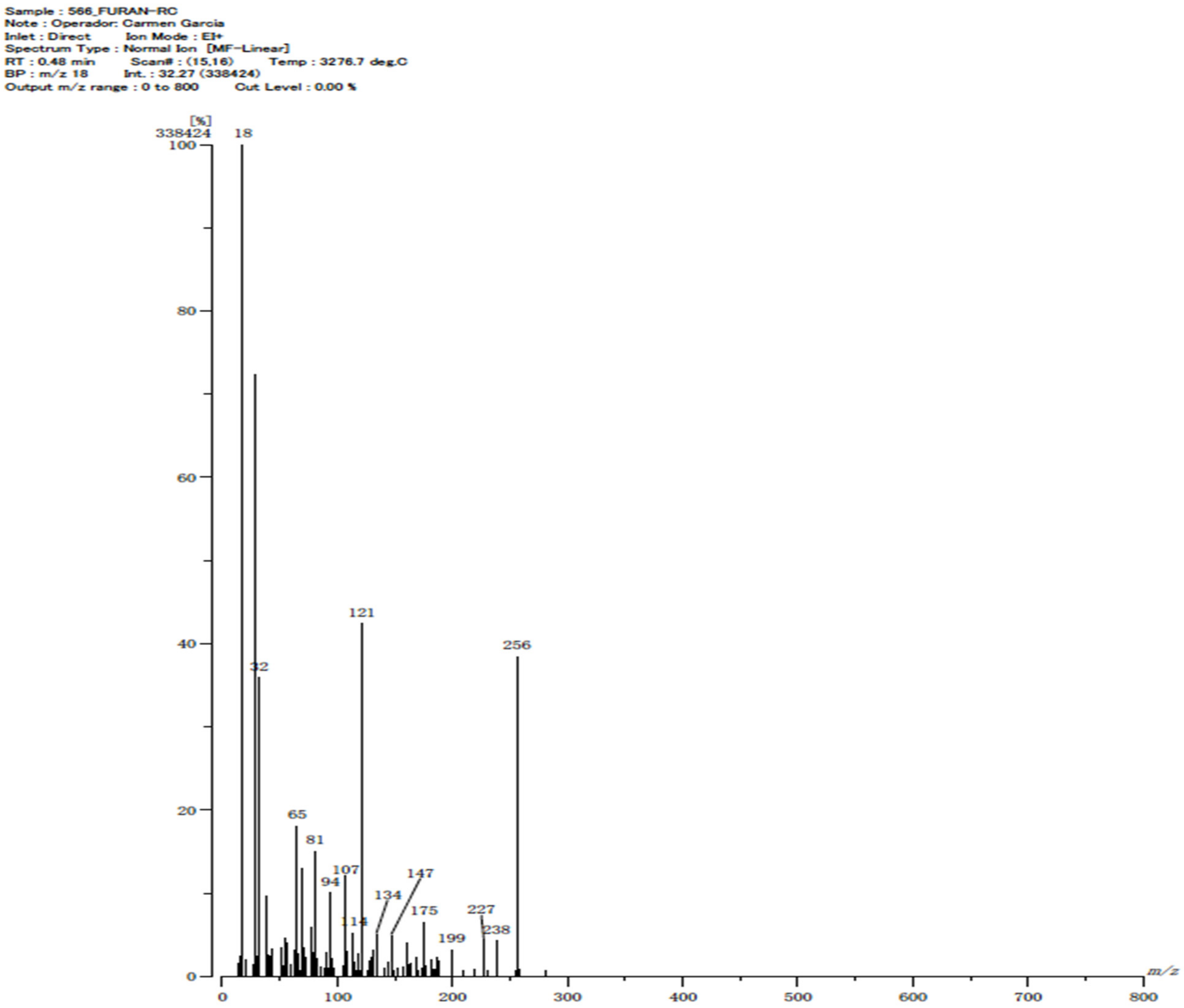

Figure S54. SM of compound **7**.

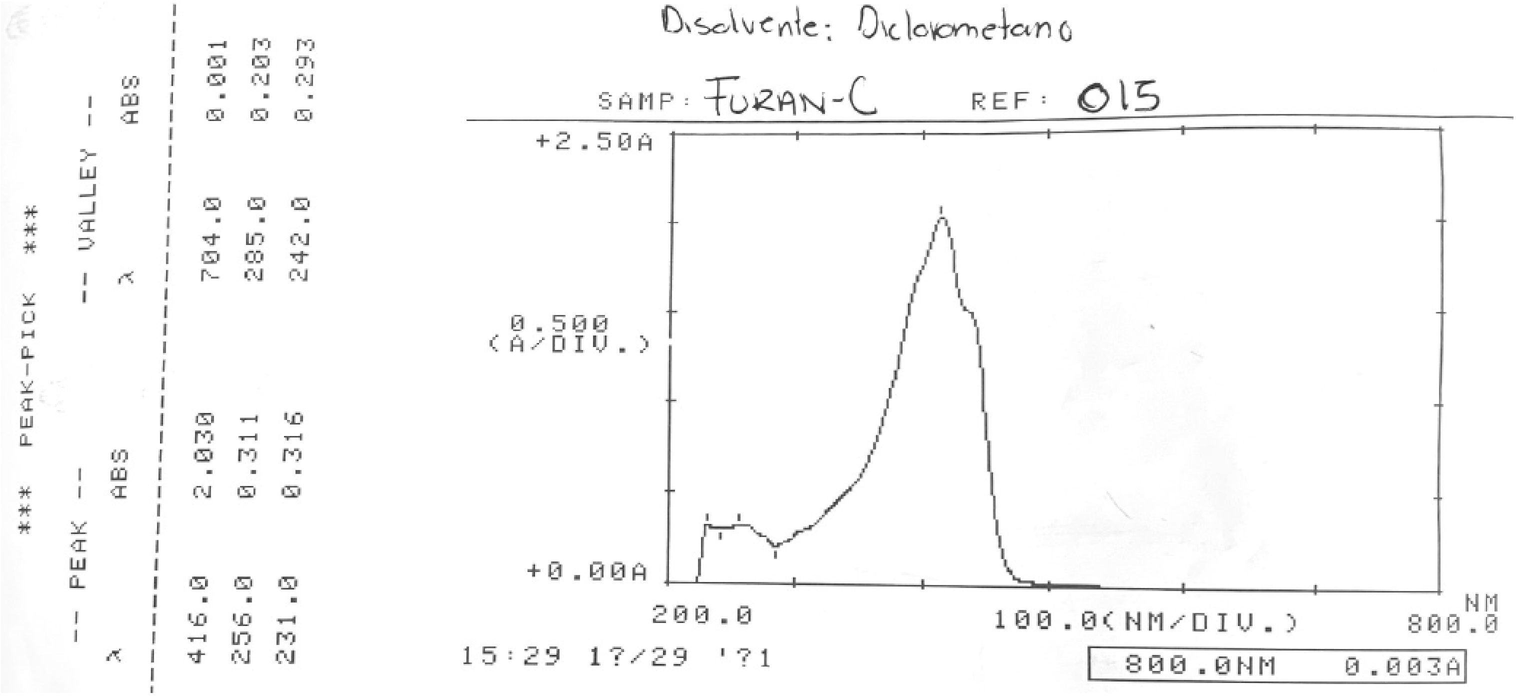

Figure S55. UV-spectrum of compound **7**.

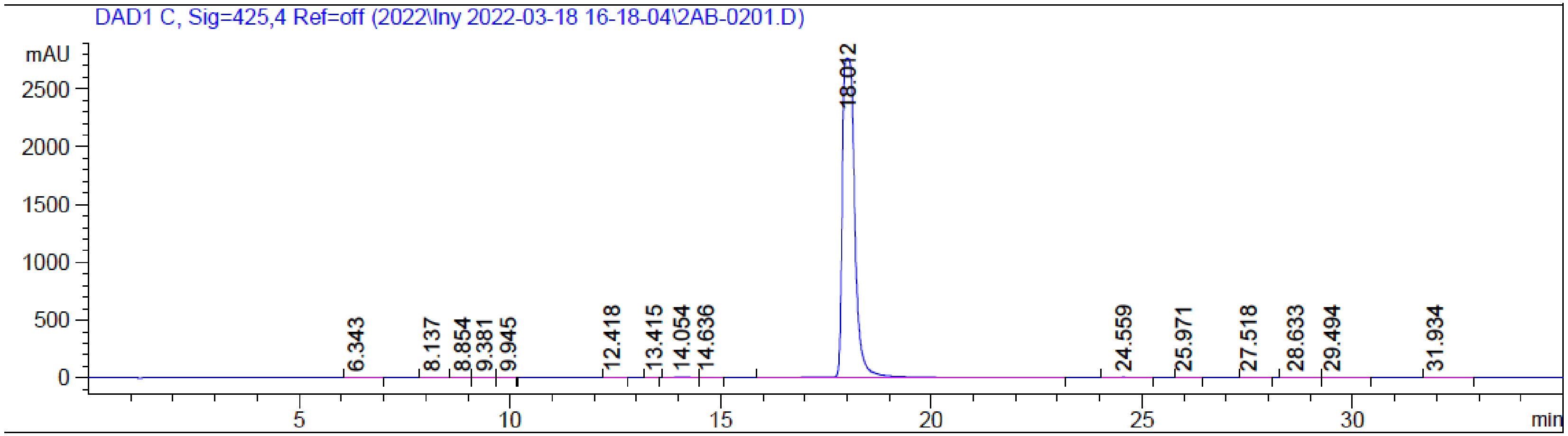

Peak #	RetTime [min]	Type	Width [min]	Area [mAU*s]	Height [mAU]	Area %
1	6.343	BB	0.2932	12.61454	6.10135e-1	0.0219
2	8.137	BV R	0.2770	63.40073	3.34631	0.1103
3	8.854	VV E	0.2257	2.36512	1.25611e-1	4.114e-3
4	9.381	VV E	0.2726	5.15727	2.25721e-1	8.972e-3
5	9.945	VB E	0.2618	2.34206	1.07646e-1	4.074e-3
6	12.418	BV R	0.1774	4.07692	2.98304e-1	7.092e-3
7	13.415	BB	0.1595	1.87429	1.50328e-1	3.261e-3
8	14.054	BV	0.3764	139.91582	5.47125	0.2434
9	14.636	VB	0.2270	40.73635	2.62541	0.0709
10	18.012	BV R	0.3212	5.71032e4	2761.48315	99.3367
11	24.559	VB R	0.2489	68.03589	4.04835	0.1184
12	25.971	BB	0.1819	5.99880	4.79577e-1	0.0104
13	27.518	BB	0.2614	3.39260	1.62647e-1	5.902e-3
14	28.633	BV	0.2917	8.84350	3.97265e-1	0.0154
15	29.494	VV R	0.2347	17.05420	1.03259	0.0297
16	31.934	BV R	0.2554	5.46527	2.90593e-1	9.507e-3
Totals :				5.74845e4	2780.85491	

Figure S56. HPLC of compound **7** (425nm).

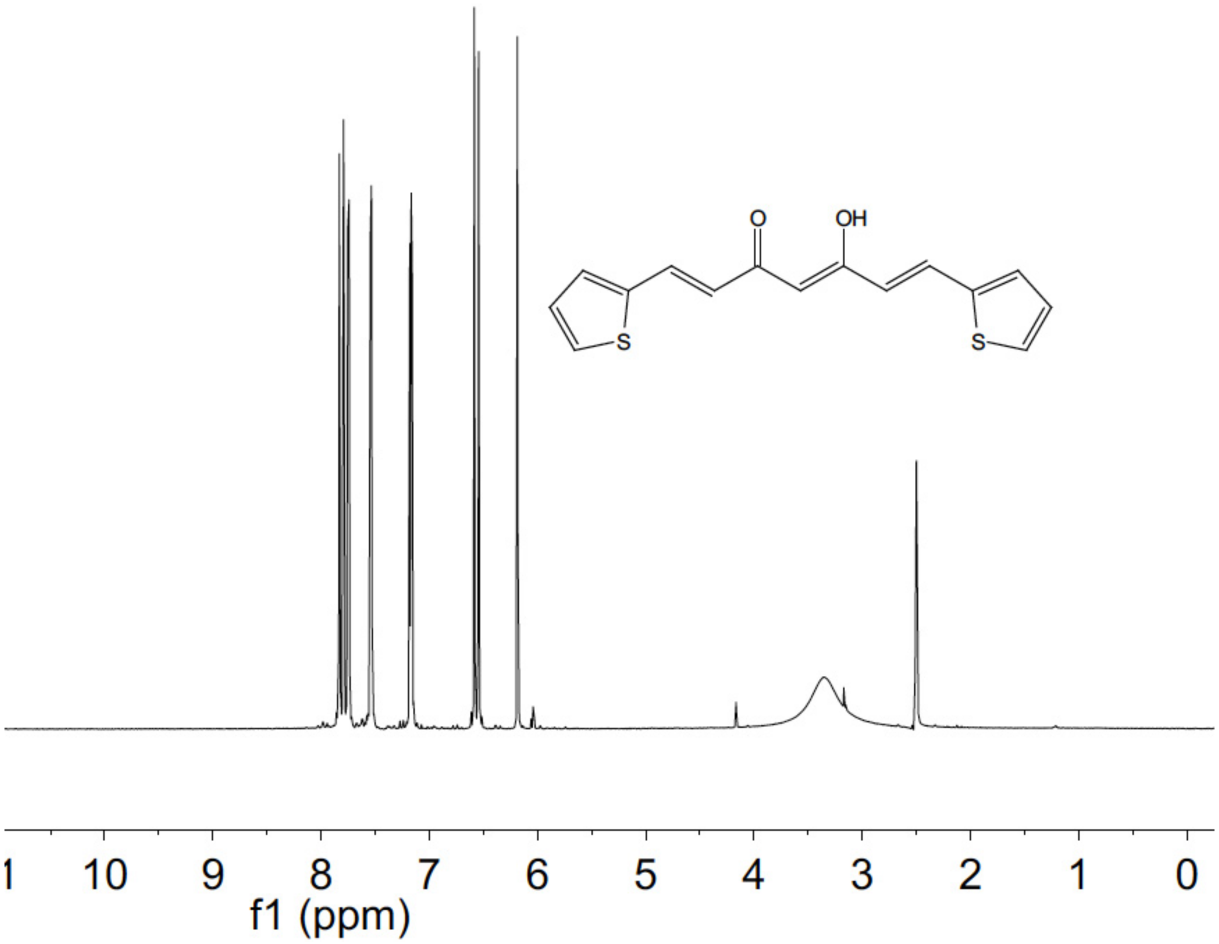

Figure 57. ^{1}H NMR spectrum of compound **8** (DMSO-*d6*-400MHz).

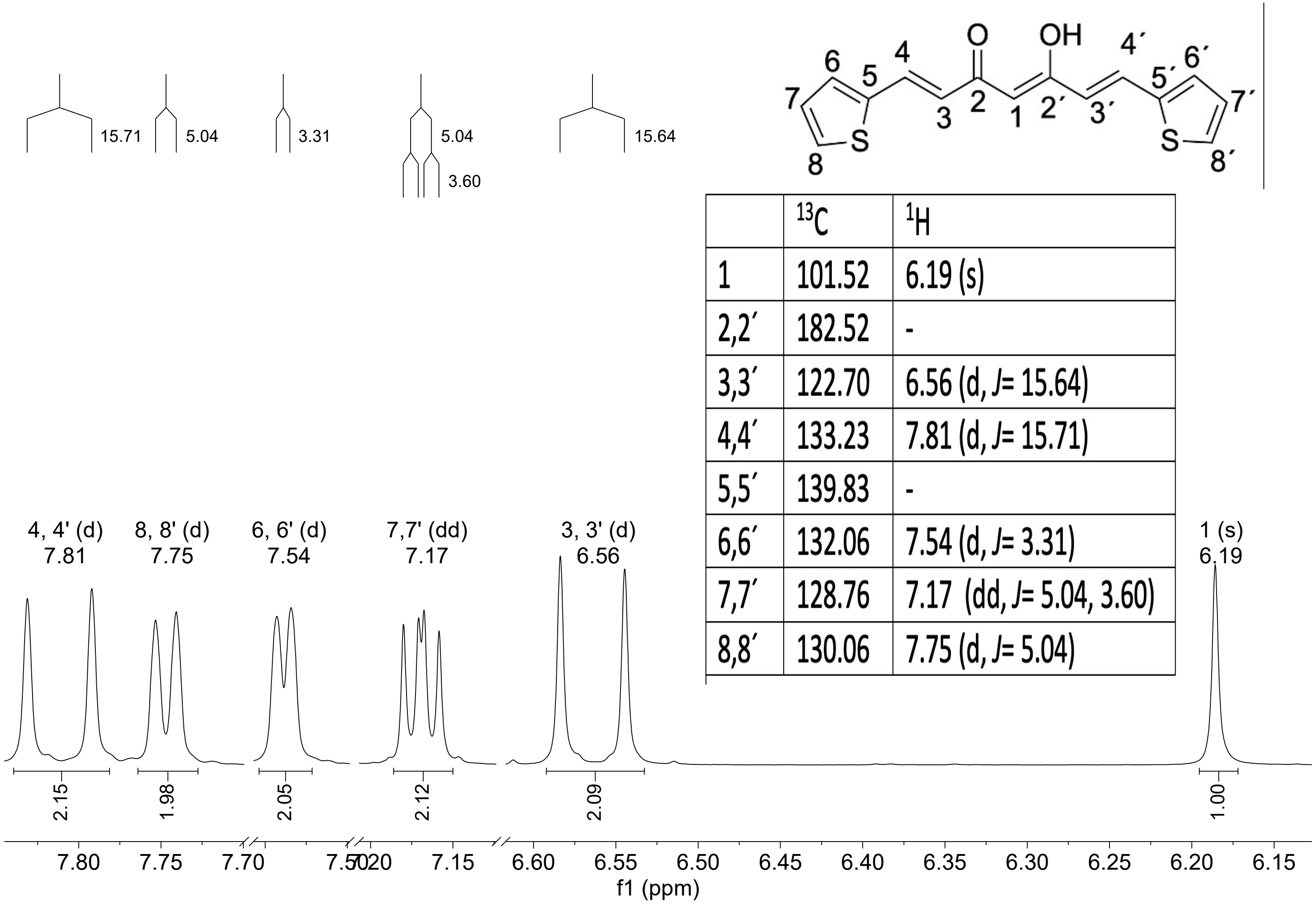

	^{13}C	^{1}H
1	101.52	6.19 (s)
2,2′	182.52	-
3,3′	122.70	6.56 (d, *J*= 15.64)
4,4′	133.23	7.81 (d, *J*= 15.71)
5,5′	139.83	-
6,6′	132.06	7.54 (d, *J*= 3.31)
7,7′	128.76	7.17 (dd, *J*= 5.04, 3.60)
8,8′	130.06	7.75 (d, *J*= 5.04)

Figure S58. ^{1}H NMR spectrum of compound **8** (DMSO-*d6*-400MHz, expansion).

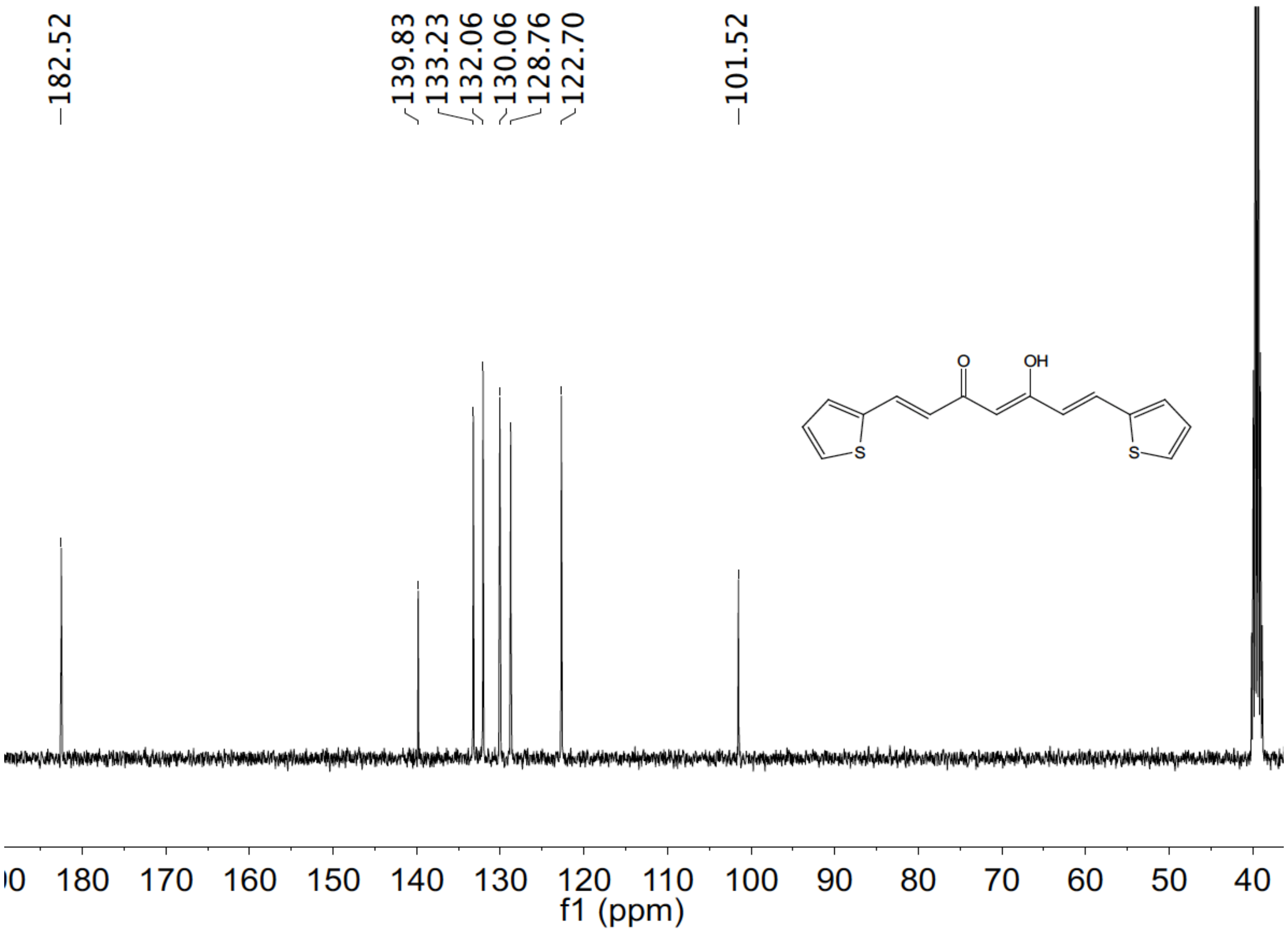

Figure S59. ^{13}C NMR spectrum of compound **8** (DMSO-*d6*-100MHz).

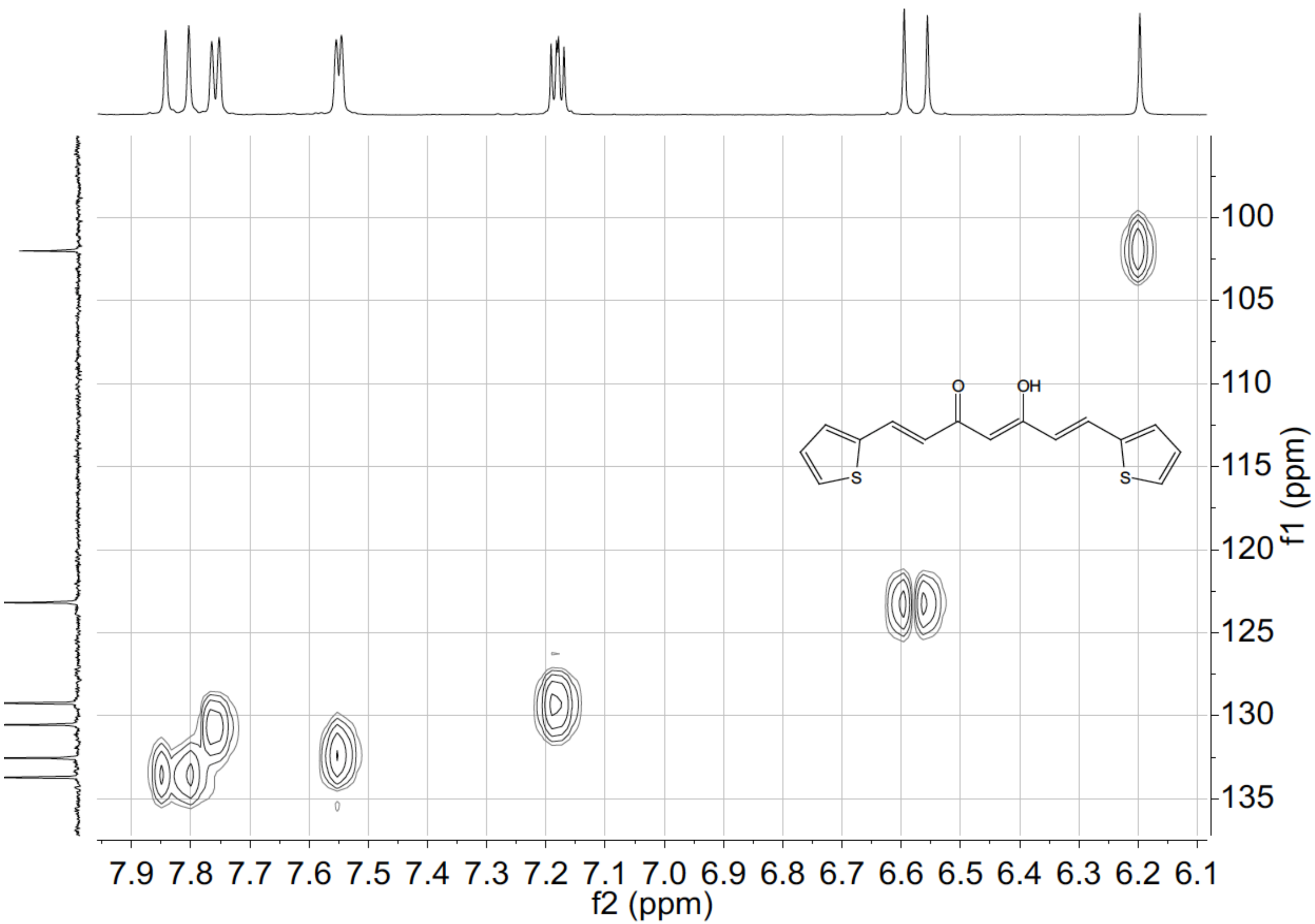

Figure S60. HSQC NMR spectrum of compound **8** (DMSO-*d6*-400MHz).

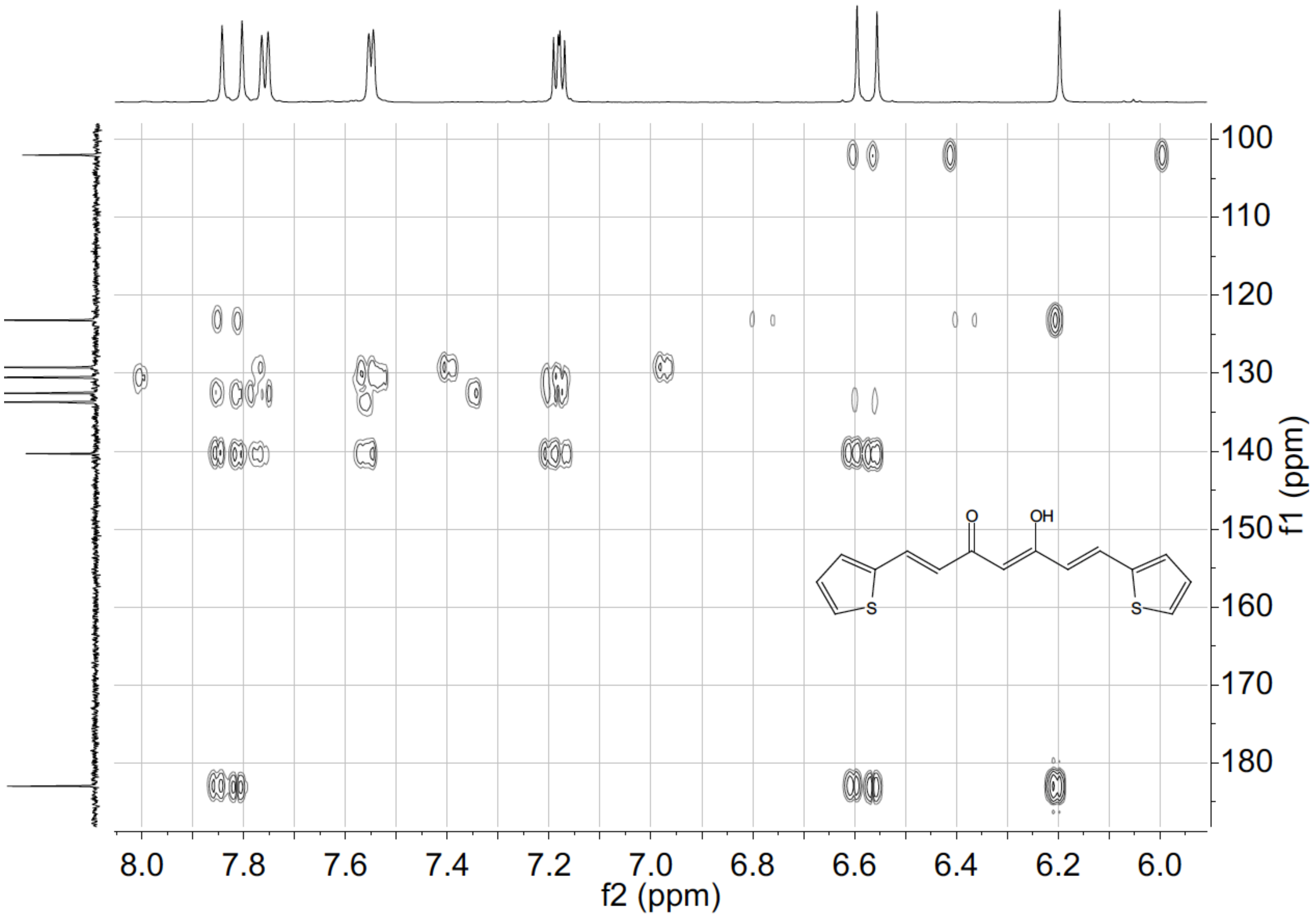

Figure S61. HMBC NMR spectrum of compound **8** (DMSO-*d6*-400MHz).

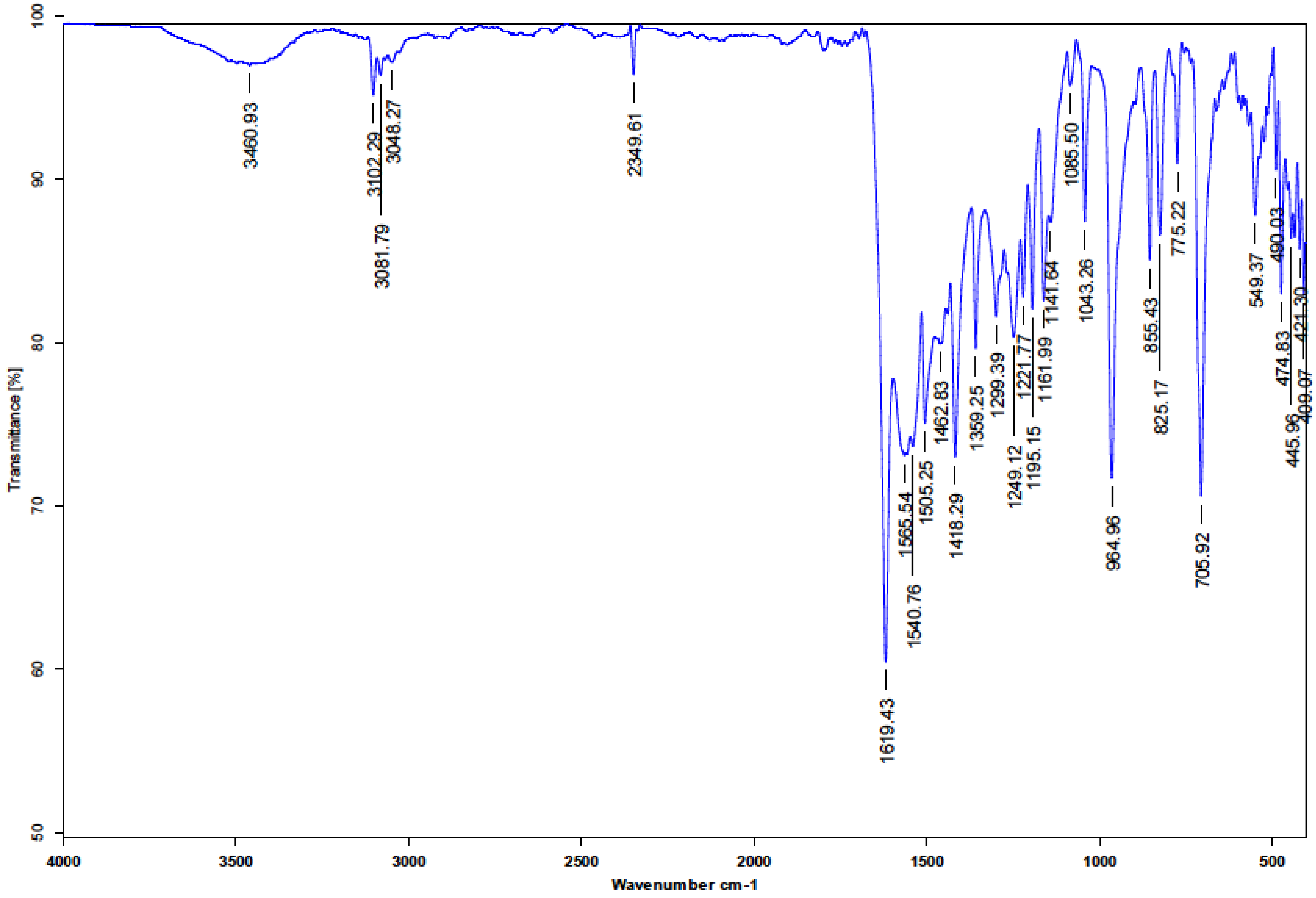

Figure S62. IR spectrum of compound **8**.

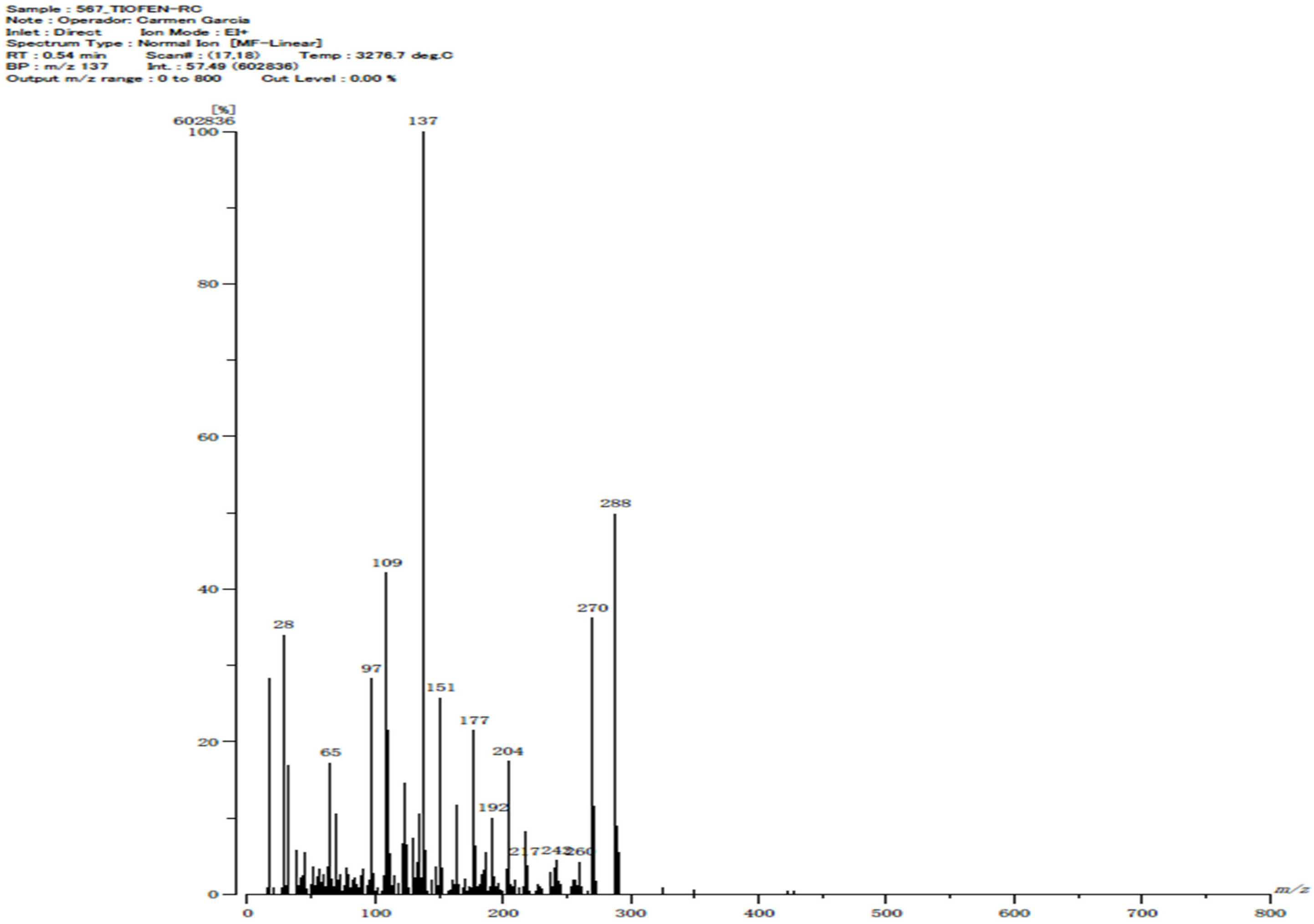

Figure S63. SM of compound **8**.

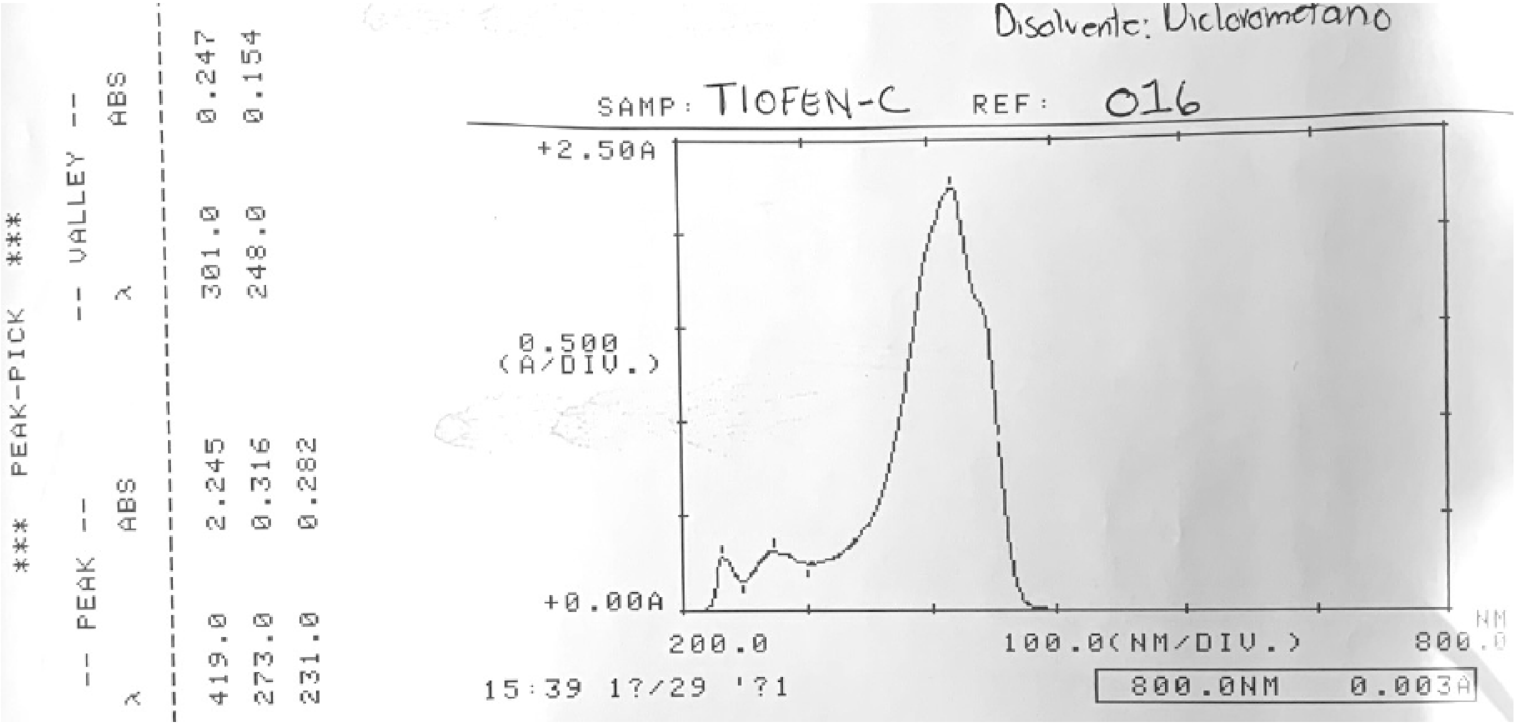

Figure S64. UV-spectrum of compound **8**.

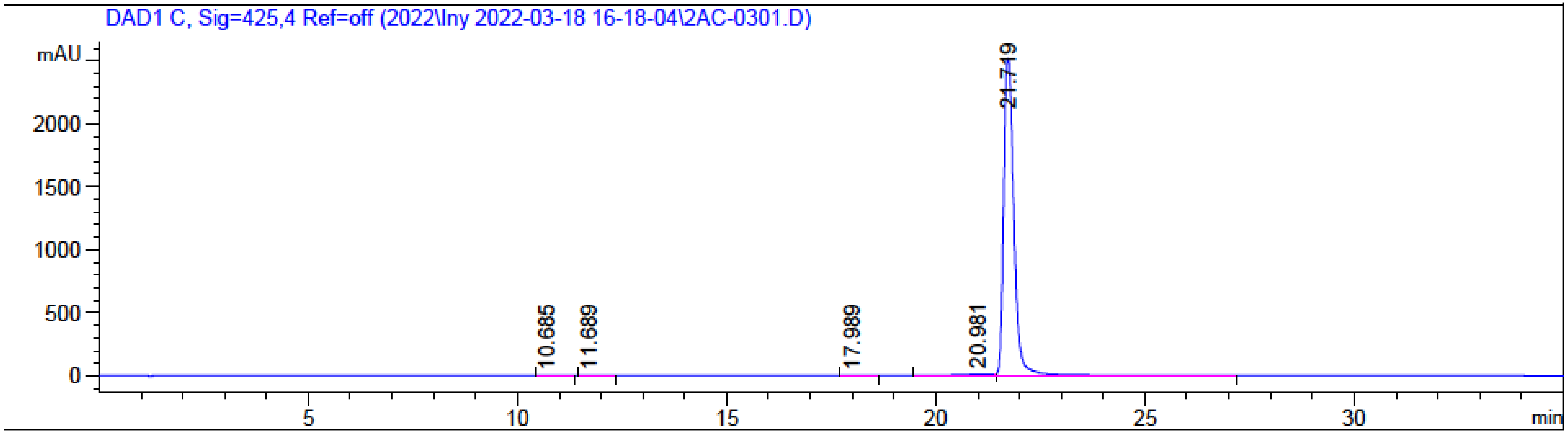

Signal 2: DAD1 C, Sig=425,4 Ref=off

Peak #	RetTime [min]	Type	Width [min]	Area [mAU*s]	Height [mAU]	Area %
1	10.685	BV R	0.2321	11.45039	6.74358e-1	0.0263
2	11.689	BB	0.2776	32.45408	1.70894	0.0745
3	17.989	BB	0.2627	28.49121	1.65650	0.0654
4	20.981	BV E	0.5686	502.61679	11.52888	1.1535
5	21.719	VB R	0.2651	4.29988e4	2520.15991	98.6804

Totals : 4.35738e4 2535.72859

Figure S65. HPLC of compound **8** (425nm).

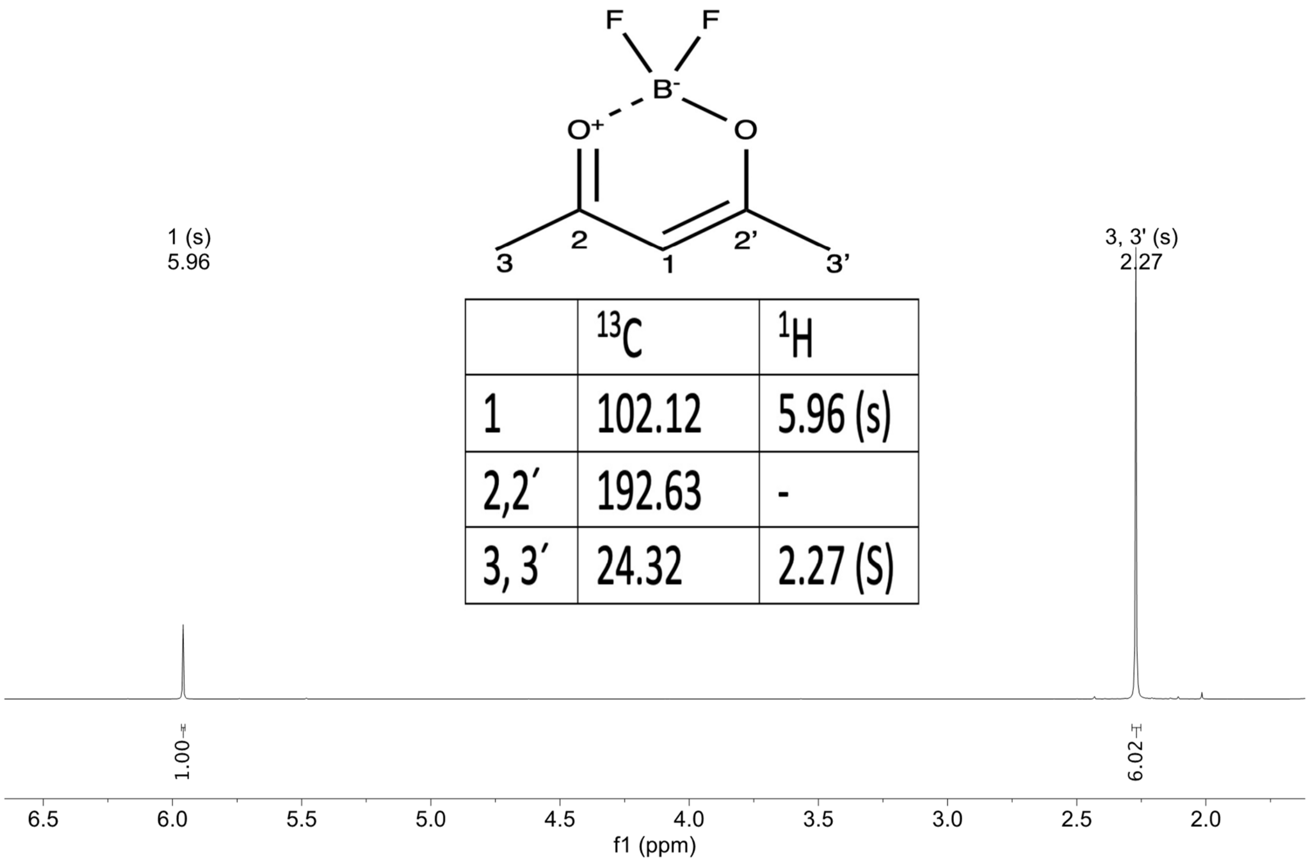

	^{13}C	^{1}H
1	102.12	5.96 (s)
2,2′	192.63	-
3, 3′	24.32	2.27 (S)

Figure 66. ^{1}H NMR spectrum of *synthon I* ($CDCl_3$-400MHz).

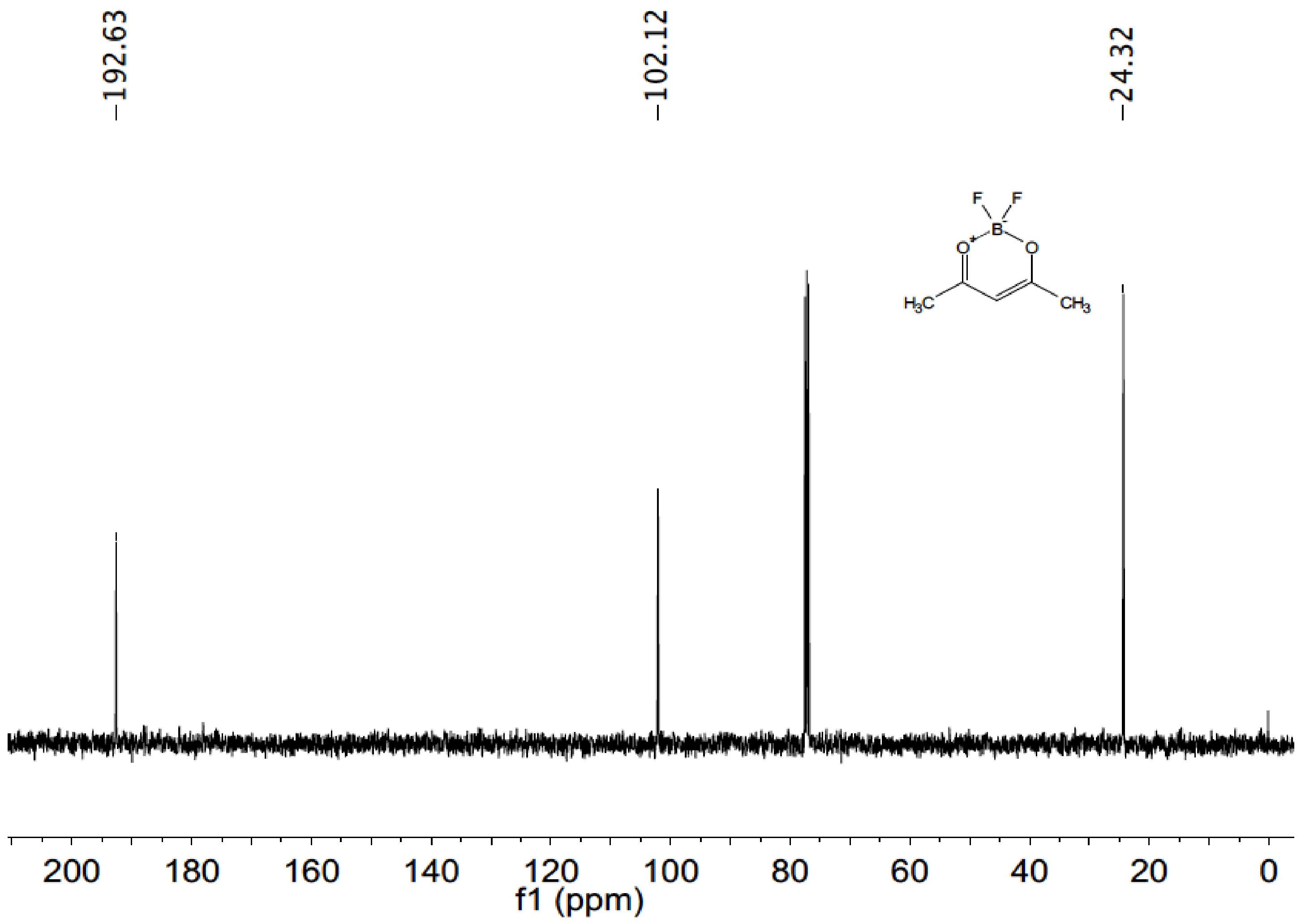

Figure S67. ^{13}C NMR spectrum of *synthon I* ($CDCl_3$-100MHz).

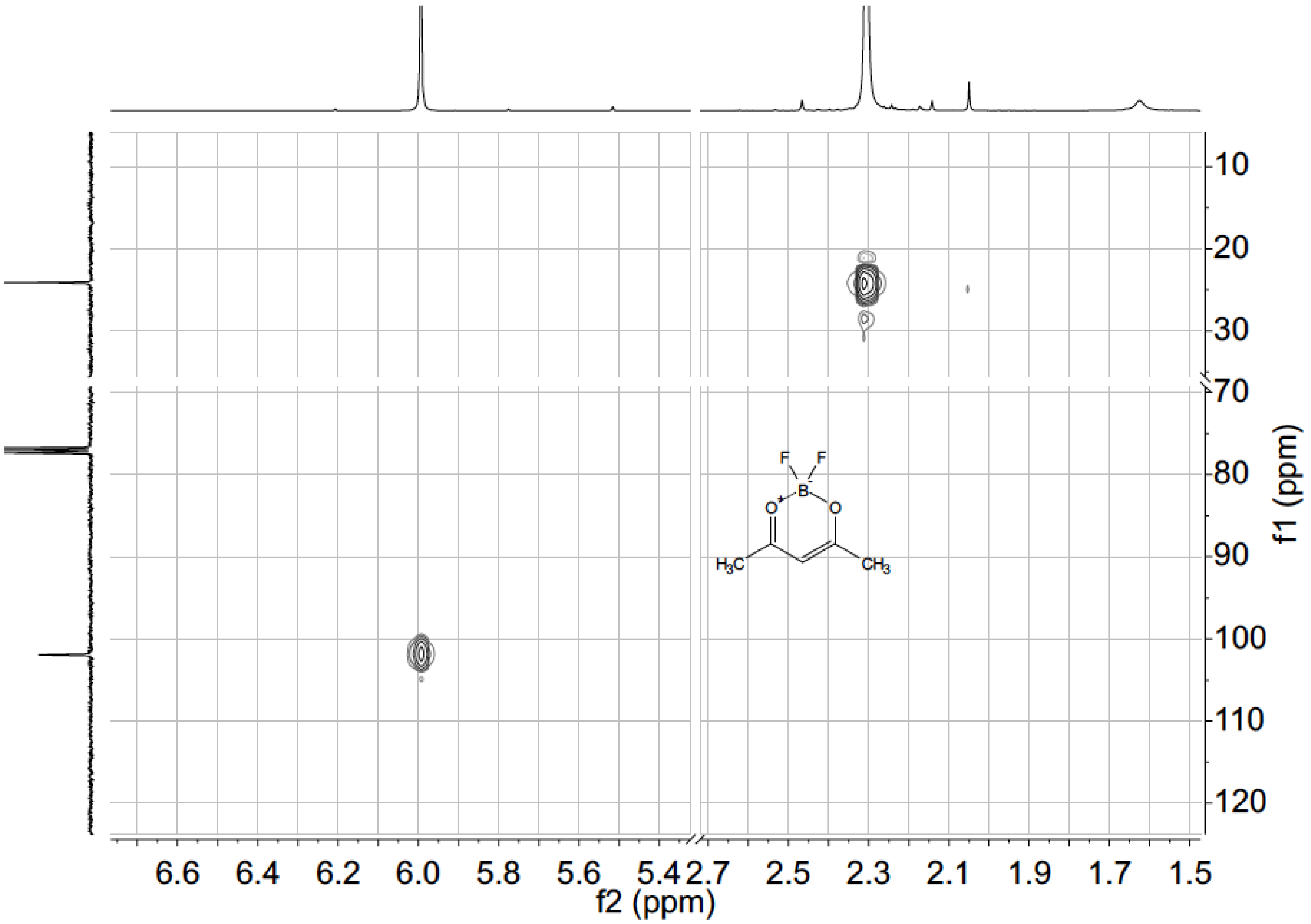

Figure S68. HSQC NMR spectrum of *synthon I* ($CDCl_3$-400MHz).

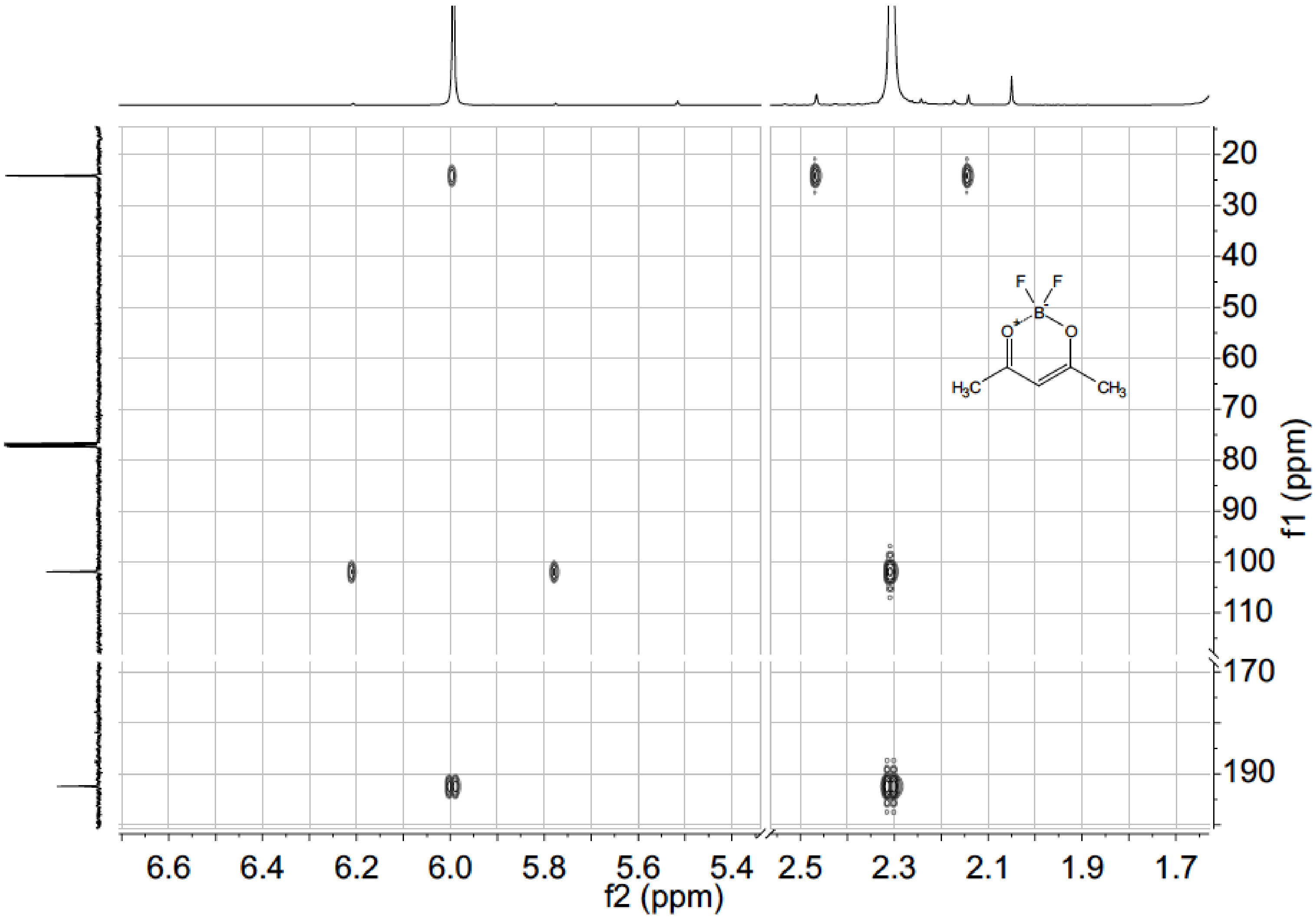

Figure S69. HMBC NMR spectrum of *synthon I* ($CDCl_3$-400MHz).

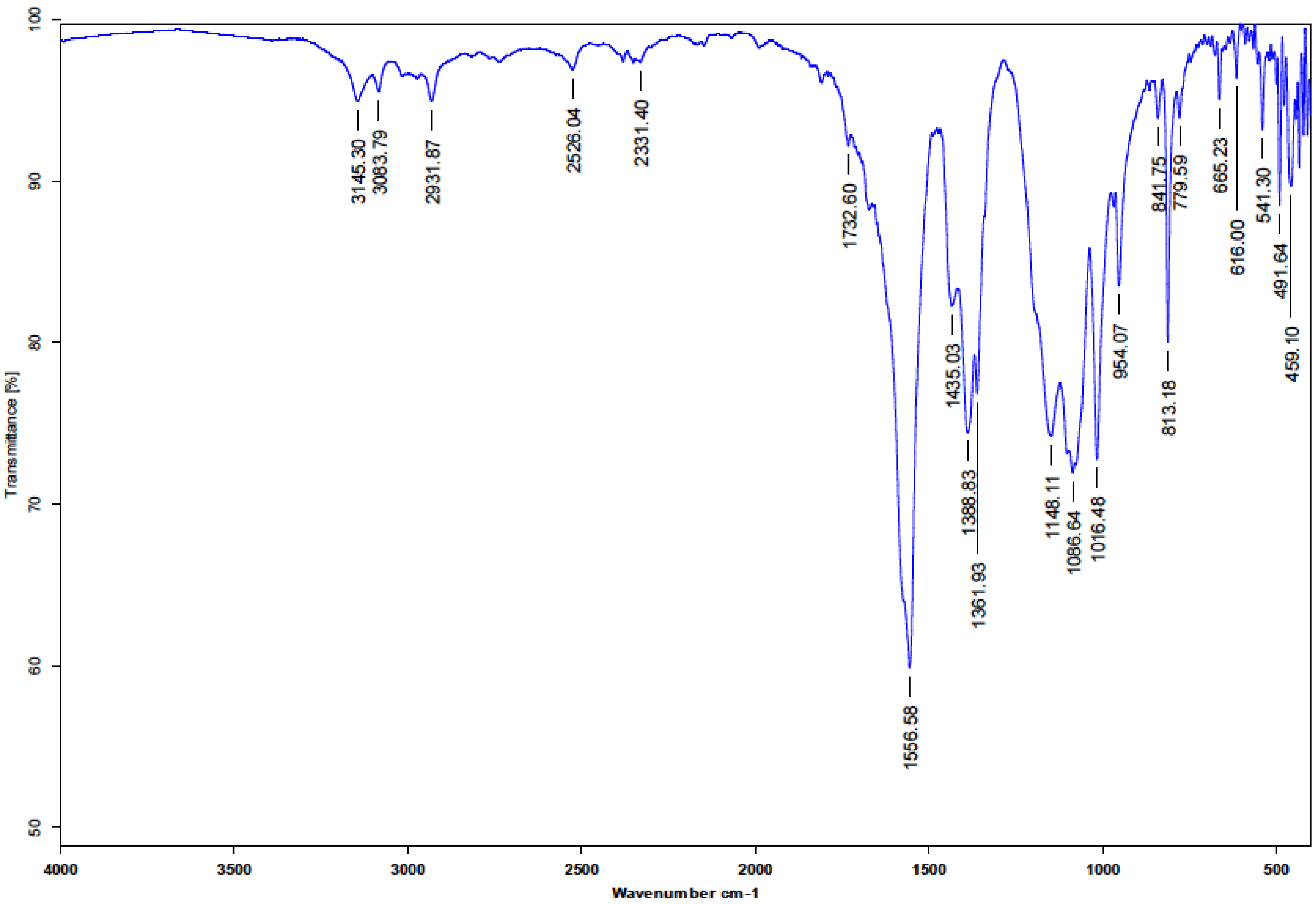

Figure S70. IR spectrum of *synthon I*.

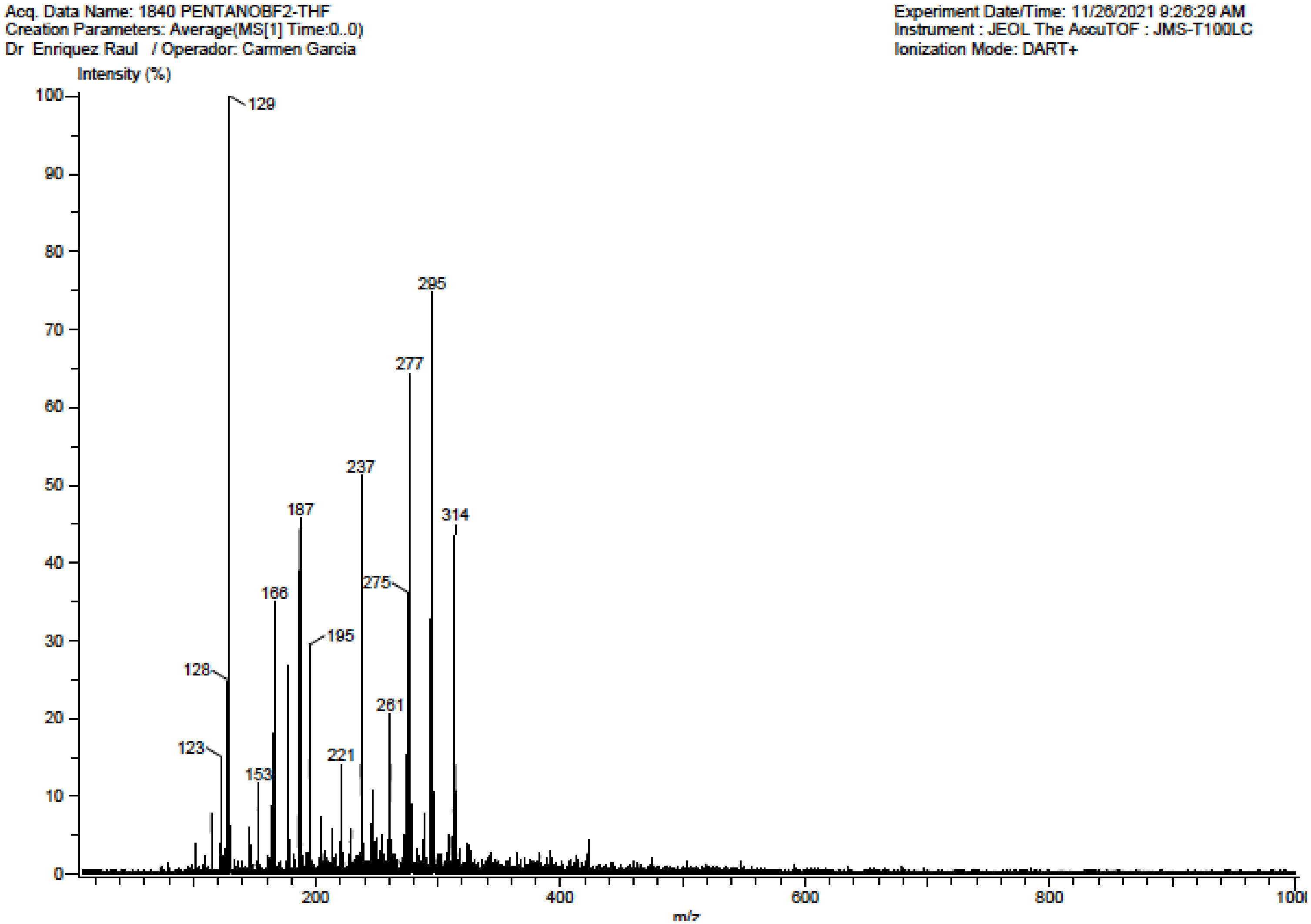

Figure S71. SM of *synthon I*.
